# Polar Codes for Covert Communications over Asynchronous Discrete Memoryless Channels

**DOI:** 10.3390/e20010003

**Published:** 2017-12-22

**Authors:** Guillaume Frèche, Matthieu R. Bloch, Michel Barret

**Affiliations:** 1 UMI 2958 Georgia Tech-CNRS, 57070 Metz, France; 2 School of Electrical and Computer Engineering, Georgia Institute of Technology, Atlanta, GA 30332, USA; 3 CentraleSupélec, 57070 Metz, France

**Keywords:** physical-layer security, covert communication, polar codes

## Abstract

This paper introduces an explicit covert communication code for binary-input asynchronous discrete memoryless channels based on binary polar codes, in which legitimate parties exploit uncertainty created by both the channel noise and the time of transmission to avoid detection by an adversary. The proposed code jointly ensures reliable communication for a legitimate receiver and low probability of detection with respect to the adversary, both observing noisy versions of the codewords. Binary polar codes are used to shape the weight distribution of codewords and ensure that the average weight decays as the block length grows. The performance of the proposed code is severely limited by the speed of polarization, which in turn controls the decay of the average codeword weight with the block length. Although the proposed construction falls largely short of achieving the performance of random codes, it inherits the low-complexity properties of polar codes.

## 1. Introduction

Following the proof of existence of a “square root law” [1] for covert communication, several works have revisited the problem of communicating while ensuring a low probability of detection by an adversary. The square root law essentially states that, under mild conditions about the channel, covert communication is possible if and only if the number of message bits scales as the square root of the block length. The exact information-theoretic limits of covert communication over point-to-point Discrete Memoryless Channels (DMCs) and Gaussian channels are now known [2,3,4], and for models relaxing assumptions regarding channel knowledge and synchronicity, the square root law can be circumvented [5,6]. Despite recent results showing, through a random coding argument, the existence of low-complexity covert codes using a concatenated scheme [7], i.e., codes ensuring covert communication with an encoding and decoding complexity that only scale *linearly* with the block length, no explicit low-complexity constructions are known to date.

As highlighted in [3], the coding mechanism behind covert communication may be linked to the concept of *channel output approximation* [8], which allows us to leverage recent error control coding approaches to secrecy exploiting similar ideas [9]. However, the main challenge faced when designing explicit instantiations of covert codes is the control of the codeword weight distribution, whose average weight should scale *sub-linearly* with the block-length [2,3,4].

In this paper, we develop a polar-code based covert code for the asynchronous covert communication model of [6]. The choice of polar codes is motivated by their low-complexity and capacity-achieving properties [10], which have already proved useful in the context of channel resolvability [11]. Existing results, however, do not directly apply to covert communications since the average codeword weight must decay with the block length. We address this issue by first adapting the finite-length analysis of channel polarization [12,13] to source polarization and then analyzing the tension between the speed of polarization and the decay of the average codeword weight.

The remainder of the paper is organized as follows. Section 2 formally introduces the model of covert communication and presents our main contributions. Section 3 establishes several preliminary technical lemmas concerning the polarization of sources with vanishing entropy. Section 4 describes the proposed polar-coding scheme for covert communications and analyzes its performance. Section 5 concludes the paper with a discussion of extensions and possible improvements.

## 2. Asynchronous Covert Communication Model and Results

### 2.1. Notation

Before describing the asynchronous covert communication model, we briefly introduce the notation used throughout the paper. Random variables are denoted by upper case letters, e.g., *X*, and their realizations by lower case letters, e.g., *x*. Sets are denoted with calligraphic fonts, e.g., X. Vectors of length *n* are denoted as $X^{1:n}=\left(X_{1},\cdots ,X_{n}\right)$ and $x^{1:n}=\left(x_{1},\cdots ,x_{n}\right)$ when the length needs to be explicit, and by boldface fonts, e.g., X and x, when the length can be inferred from the context without ambiguity. When multiple blocks of length *n* are used, we denote the block index as a subscript, e.g, $X_{1:b}^{1:n}$ denotes a sequence of *b* blocks of length *n*. The function log is understood in the base 2, while ln denotes the logarithm to the base *e*. For two distributions P,Q on some countable set X, we write the Kullback–Leibler divergence and the total variation distance as

$$\begin{array}{c} D\left(P∥Q\right)≜\sum_{x\in X}P\left(x\right)\log\frac{P(x)}{Q(x)}\;\mathrm{and}\;V\left(P,Q\right)≜\frac{1}{2}\sum_{x\in X}\left|P\left(x\right)-Q\left(x\right)\right|, \end{array}$$

respectively. We also denote $P^{⊗n}\left(x\right)$ as the product distribution $\prod_{i=1}^{n}P\left(x_{i}\right)$ for $x\in X^{n}$.

We make repeated use of the Landau notation. In particular, for two real-valued functions f(n) and g(n) of n∈N, we write $f\left(n\right)=o\left(g(n)\right)$ if ∀α>0 $\exists n_{0}\in N^{*}$ such that $\forall n\geq n_{0}$ $\left|f(n)\right|\leq \alpha \left|g(n)\right|$; $f\left(n\right)=O\left(g(n)\right)$ if ∃α>0 $\exists n_{0}\in N^{*}$ such that $\forall n\geq n_{0}$ $\left|f(n)\right|\leq \alpha \left|g(n)\right|$; $f\left(n\right)=\omega \left(g(n)\right)$ if ∀α>0 $\exists n_{0}\in N^{*}$ such that $\forall n\geq n_{0}$ $\left|f(n)\right|\geq \alpha \left|g(n)\right|$.

The polarization kernel matrix $G_{2}=\left[\begin{array}{cc} 1 & 1\\ 0 & 1 \end{array}\right]$ will be merely denoted *G*. We denote $G^{⊗\nu }$ the matrix representing the recursive transformation over ν levels of polarization. Thus, the corresponding polar code is of length $n=2^{\nu }$. Since the length of binary polar codes is a power of two, we restrict our attention to block lengths $n\in D≜\{2^{\nu }:\nu \in N^{*}\}$.

### 2.2. Channel Model

**Figure 1:**
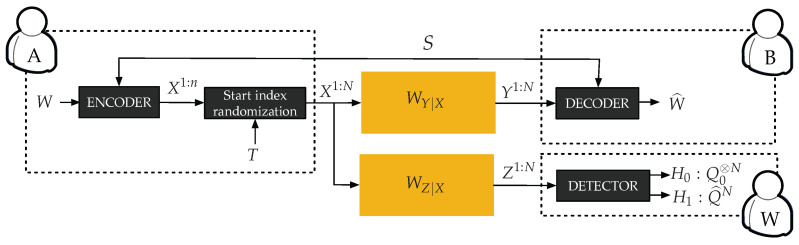
Model of covert communication.

**Figure 2:**
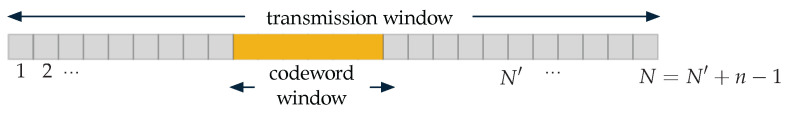
Asynchronous covert communication.

The channel model for covert communication is illustrated in Figure 1 and Figure 2. A legitimate transmitter (Alice) attempts to reliably communicate to a legitimate receiver (Bob) over a DMC $\left(X,W_{Y|X},Y\right)$, while avoiding detection from an adversary (Willie) who observes signals through another DMC $\left(X,W_{Z|X},Z\right)$. In the remainder of the paper, we restrict our attention to a binary input alphabet X={0,1}, with 0 representing the innocent input symbol in the absence of communication. We denote $P_{0}≜W_{Y|X=0}$ and $Q_{0}≜W_{Z|X=0}$ as the output distributions induced by the innocent symbol 0. Similarly, we denote $P_{1}≜W_{Y|X=1}$ and $Q_{1}≜W_{Z|X=1}$ as the output distributions induced by symbol 1. We assume that both $P_{1}$ and $Q_{1}$ are absolutely continuous with respect to (w.r.t.) $P_{0}$ and $Q_{0}$, respectively, to avoid the special situations discussed in Appendix V of [3].

Formally, a message $W\in 〚1,M_{n}〛$ with uniform distribution is encoded into a codeword of length *n*, possibly with the help of secret key $S\in 〚1,K_{n}〛$ only known to Alice and Bob but using a public codebook known to all parties; the codeword is hidden within a larger *transmission window* of size N>n, with *N* a function of *n*, by choosing the starting index *T* of the codeword uniformly at random between 1 and $N^{\prime }≜N-n+1$. The set of indices corresponding to the codeword forms the *codeword window*. The sequence transmitted during the transmission window is denoted $X^{1:N}$, and the corresponding observations of Bob and Willie are denoted $Y^{1:N}$ and $Z^{1:N}$, respectively. It is convenient to introduce the following distributions. The distribution induced at the output of the adversary’s channel in a codeword window is denoted ${\hat{Q}}^{n}$. When the codeword is embedded in a transmission window starting at a known index *t*, the distribution induced at the output of the adversary’s channel in the transmission window is

(1) $$\begin{array}{c} {\hat{Q}}_{t}^{N}\left(z\right)=\prod_{k=1}^{t-1}Q_{0}\left(z_{k}\right)\prod_{k=t}^{t+n-1}{\hat{Q}}^{n}\left(z_{k}\right)\prod_{k=t+n}^{N}Q_{0}\left(z_{k}\right). \end{array}$$

Finally, the distribution induced at the output of the adversary’s channel in the transmission window when *randomizing* the start index *T* is ${\hat{Q}}^{N}≜E_{T}\left({\hat{Q}}_{T}^{N}\right)$.

Given the secret key *S* and the observation $Y^{1:N}$, Bob forms an estimate $\hat{W}$ of the original message *W*, whose performance is measured by the probability of error $P_{e}^{\left(n\right)}≜E_{S}\left(P\left(\hat{W}\neq W|S\right)\right)$. Given the observation $Z^{1:N}$ and the knowledge of Alice’s codebook, Willie performs a hypothesis test to determine if communication took place. Hypothesis $H_{0}$ corresponds to the absence of communication, in which case the distribution of $Z^{1:N}$ is $Q_{0}^{⊗N}$; Hypothesis $H_{1}$ corresponds to communication, in which case the distribution induced by the code is ${\hat{Q}}^{N}$ over the transmission window. Note that ${\hat{Q}}^{N}$ can be computed using knowledge of the codebook and the distribution of *T*. The covertness of the transmission is measured by the total variation $V^{\left(n\right)}≜V\;\left({\hat{Q}}^{N},Q_{0}^{⊗N}\right)$. A small value of $V^{\left(n\right)}$ ensures that the best binary hypothesis test is not significantly better than a “blind” test that would ignore the observation $Z^{1:N}$ [3].

Our objective is to construct sequences of codes such that $\lim_{n\to \infty }P_{e}^{\left(n\right)}=0$ and $\lim_{n\to \infty }V^{\left(n\right)}=0$.

### 2.3. Main Results

We start by recalling a known result established with a random coding argument, which serves as a benchmark for our code construction.

**Proposition** **1.** (adapted from [6]). Consider sequences of positive numbers ${\left\{\alpha_{n}\right\}}_{n\in N^{*}}$, ${\left\{\beta_{n}\right\}}_{n\in N^{*}}$ such that $\alpha_{n}\in \omega \left(\frac{1}{\sqrt{n}}\right)\cap o\left(1\right)$, $\beta_{n}=\omega \left(2^{-\frac{n\alpha_{n}}{\log n}}\right)\cap o\left(1\right)$ as n goes to infinity. Let $N=\frac{2^{n\alpha_{n}^{2}}}{\beta_{n}\alpha_{n}^{2}}$. There exist codes of increasing block length n hidden in transmission windows of size N such that

$$\begin{array}{cc} \lim_{n\to \infty }\frac{\log M_{n}}{n\alpha_{n}} & \geq D\;\left(P_{1}∥P_{0}\right)\\ \lim_{n\to \infty }\frac{\log K_{n}}{n\alpha_{n}} & \leq {\left[D\;\left(Q_{1}∥Q_{0}\right)-D\;\left(P_{1}∥P_{0}\right)\right]}^{+}\\ \lim_{n\to \infty }P_{e}^{\left(n\right)} & =0\\ \lim_{n\to \infty }V^{\left(n\right)} & =0. \end{array}$$

Proposition 1 states that the number of bits $\log M_{n}$ scales as $n\alpha_{n}$ with a constant pre-factor at least equal to $D\;\left(P_{1}∥P_{0}\right)$ for all admissible choices of $\alpha_{n}$. As $\alpha_{n}$ increases, so does the scaling of $\log M_{n}$, but at the expense of increasingly larger monitoring windows. Proposition 1 captures the correct scaling for the transmission window size, the number of message bits, and the number of key bits with the block length *n*, as shown by the converse proof in [5]. While this result has been obtained with a random coding argument, in which codewords are sampled independently according to product distributions, the main contribution of the present paper is to establish a similar result using polar codes in place of random codes.

In the following, we allow ourself a slight modification of the coding scheme defined in Section 2.2 to consider $b_{n}$ consecutive transmission windows of size *N*, where $b_{n}$ will be specified later. The messages and keys used in the transmission windows might be dependent, but the codeword in each of them is otherwise created as defined earlier. The probability of error $P_{e}^{\left(n\right)}$ is appropriately modified to consider the set of messages ${\left\{W_{i}\right\}}_{i=1}^{b_{n}}$ as

(2) $$\begin{array}{c} P_{e}^{\left(n\right)}=P\left({\left\{{\hat{W}}_{i}\right\}}_{i=1}^{b_{n}}\neq {\left\{W_{i}\right\}}_{i=1}^{b_{n}}\right), \end{array}$$

and $V^{\left(n\right)}$ considers the distribution induced over the $b_{n}$ consecutive transmission windows

(3) $$\begin{array}{c} V^{\left(n\right)}=V\;\left({\hat{Q}}^{b_{n}N},Q_{0}^{⊗b_{n}N}\right). \end{array}$$

Our results also depend on a constant κ, whose value results from the analysis of finite length polarization and is further discussed in Section 3.

**Proposition** **2.** *There exists a constant $\kappa \in ]0,\frac{1}{2}[$, such that for all sequences of positive numbers ${\left\{\alpha_{n}\right\}}_{n\in D}\in \omega \left(\frac{1}{n^{\kappa }}\right)\cap o\left(1\right)$, ${\left\{\beta_{n}\right\}}_{n\in D}\in \omega \left(2^{-\frac{n\alpha_{n}}{\log n}}\right)\cap o\left(\frac{1}{\log n}\right)$, and sequence of integers ${\left\{b_{n}\right\}}_{n\in D}\in \omega \left(\log n\right)\cap o\left(\frac{1}{\beta_{n}}\right)\cap o\left(n\right)$ as n goes to infinity, there exist low-complexity polar-code based schemes operating over $b_{n}$ transmission windows of size $N=\frac{2^{n\alpha_{n}^{2}}}{\beta_{n}\alpha_{n}^{2}}$, each embedding a codeword window of length n, with*

$$\begin{array}{cc} \lim_{n\in D\to \infty }\frac{\log M_{n}}{nb_{n}\alpha_{n}} & \geq D\;\left(P_{1}∥P_{0}\right),\\ \lim_{n\in D\to \infty }\frac{\log K_{n}}{nb_{n}\alpha_{n}} & \leq {\left[D\;\left(Q_{1}∥Q_{0}\right)-D\;\left(P_{1}∥P_{0}\right)\right]}^{+},\\ \lim_{n\in D\to \infty }P_{e}^{\left(n\right)} & =0,\\ \lim_{n\in D\to \infty }V^{\left(n\right)} & =0. \end{array}$$

**Proof.**  See Section 4. ☐

The constant κ in the statement of Proposition 2 is more precisely identified in Proposition 3. The precise code construction behind the statement is provided in Section 4, and the exact encoding and decoding algorithms are given in Algorithms 1 and 2 in Section 4.2. The complexity of both algorithms scales linearly with the number of transmission windows $b_{n}$ and as nlogn with the codeword length *n*. Note that Proposition 2 differs from Proposition 1 on two accounts. First, the polar-code based scheme only holds for a limited range of scalings for $\alpha_{n}$. A numerical investigation suggests that κ is on the order of $10^{-3}$, which completely precludes our codes from operating in the square-root law regime and requires absurdly large code length; however, if one backs away from the optimal scalings identified above, our approach does provide a low-complexity construction with provable guarantees. As further discussed in Section 3, this results from our inability to establish a faster polarization speed. In particular, as will be clear from our analysis, we rely on a fine polarization result from [12] to show that covertness holds, and the value of κ is therefore much more constrained than what would be expected by only looking at the inverse scaling exponent [14,15]. Our results might be improved by considering a “moderate deviation regime” in the same spirit as [14], but this would require a non-trivial extension of existing results, which we defer to future work. Second, the proposed scheme requires a chaining over $b_{n}$ transmission windows; we shall see in Section 4 that the chaining allows us to “realign” polarization sets. Although this chaining does not fall into the exact situation of Section 2.2 in which a single block is considered, covertness is guaranteed over the *entire* chain of blocks; in addition, a mild scaling such as $b_{n}=\omega \left(\log n\right)$ is valid so that the number of blocks may be much smaller than the block-length. Finally, the proposed code construction is non-trivial, but its performance is still far from that of the random codes in Proposition 1. Section 5 discusses several ongoing efforts to improve performance.

**Algorithm 1** Alice’s encoder**Require:**Vector *C* of $\left|V_{C}\right|$ uniformly distributed key bits;$b_{n}$ vectors ${\left\{W_{i}\right\}}_{i=1,b_{n}}$ of $\left|V_{W}\right|$ uniformly distributed message bits;$b_{n}$ vectors ${\left\{W_{i}^{\prime }\right\}}_{i=1,b_{n}}$ of $\left|V_{W^{\prime }}\right|$ uniformly distributed message bits;Vector $S_{1}$ of $\left|V_{S}\right|+\left|V_{S^{\prime }}\right|$ uniformly distributed key bits;$b_{n}-1$ vectors ${\left\{S_{i}\right\}}_{i=2,b_{n}}$ of $\left|V_{S}\right|$ uniformly distributed key bits;$b_{n}$ vectors ${\left\{S_{i}^{\prime }\right\}}_{i=1,b_{n}}$ of $\left|V_{C^{\prime }}\right|$ uniformly distributed key bits;$b_{n}$ vectors ${\left\{S_{i}^{\prime \prime }\right\}}_{i=1,b_{n}}$ of logN uniformly distributed key bits;
1:**for** block i=1 to $b_{n}$ **do**2:  ${\tilde{U}}_{i}^{1:n}\left[V_{C}\right]\leftarrow C$3:  ${\tilde{U}}_{i}^{1:n}\left[V_{W}\right]\leftarrow W_{i}$4:  ${\tilde{U}}_{i}^{1:n}\left[V_{W^{\prime }}\right]\leftarrow W_{i}^{\prime }$5:  **if** i=1 **then**6:    ${\tilde{U}}_{i}^{1:n}\left[V_{S}\right]\leftarrow S_{1}$7:  **else**8:    ${\tilde{U}}_{i}^{1:n}\left[V_{S^{\prime }}\right]\leftarrow W_{i-1}^{\prime }$9:    ${\tilde{U}}_{i}^{1:n}\left[V_{S}\right]\leftarrow S_{i}$10:  **end if**11:  Successively draw the components of ${\tilde{U}}_{i}^{1:n}$ in $V_{X}^{c}$ according to
(4)$$\forall j\in V_{X}^{c}\;{\tilde{p}}_{U_{i}^{j}|U_{i}^{1:j-1}}\left(u_{i}^{j}|{\tilde{U}}_{i}^{1:j-1}\right)≜q_{U^{j}|U^{1:j-1}}\left(u_{i}^{j}|{\tilde{U}}_{i}^{1:j-1}\right)$$12:  Transmit ${\tilde{X}}_{i}^{1:n}≜{\tilde{U}}_{i}^{1:n}G^{⊗\nu }$ over the channel $W_{Y|X}$, which gives the output ${\tilde{Y}}_{i}^{1:n}$, and over the channel $W_{Z|X}$, which gives the output ${\tilde{Z}}_{i}^{1:n}$. Assume that $C_{i}^{\prime }⊕S_{i}^{\prime }≜{\tilde{U}}_{i}^{1:n}\left[V_{C^{\prime }}\right]$ is made available at the decoder. Randomize the position of the codeword window using $S_{i}^{\prime \prime }$13:**end for**


**Algorithm 2** Bob’s decoder**Require:**Vector *C* of $\left|V_{C}\right|$ uniformly distributed key bits;Vector $S_{1}$ of $\left|V_{S}\right|+\left|V_{S^{\prime }}\right|$ uniformly distributed key bits;$b_{n}-1$ vectors ${\left\{S_{i}\right\}}_{i=2,b_{n}}$ of $\left|V_{S}\right|$ uniformly distributed key bits;$b_{n}$ vectors ${\left\{S_{i}^{\prime }\right\}}_{i=1,b_{n}}$ of $\left|V_{C^{\prime }}\right|$ uniformly distributed key bits;$b_{n}$ vectors ${\left\{S_{i}^{\prime \prime }\right\}}_{i=1,b_{n}}$ of logN uniformly distributed key bits;$b_{n}$ vectors ${\left\{C_{i}^{\prime }⊕S_{i}^{\prime }\right\}}_{i=1,b_{n}}$ of $\left|V_{C^{\prime }}\right|$ made available;
1:Form an estimate ${\hat{X}}_{1}^{1:n}$ of ${\tilde{X}}_{1}^{1:n}$ from $\left(C,S_{1},C_{1}^{\prime },{\tilde{Y}}_{1}^{1:n}\right)$2:Form the estimate ${\hat{U}}_{1}^{1:n}={\hat{X}}_{1}^{1:n}G^{⊗\nu }$3:${\hat{W}}_{1}\leftarrow {\hat{U}}_{1}^{1:n}\left[V_{W}\right]$4:${\hat{W}}_{1}^{\prime }\leftarrow {\hat{U}}_{1}^{1:n}\left[V_{W^{\prime }}\right]$5:**for** block i=2 to $b_{n}$ **do**6:  Form an estimate ${\hat{X}}_{i}^{1:n}$ of ${\tilde{X}}_{i}^{1:n}$ from $\left(C,{\hat{W}}_{i-1}^{\prime },S_{i},C_{i}^{\prime },{\tilde{Y}}_{i}^{1:n}\right)$7:  Form the estimate ${\hat{U}}_{i}^{1:n}={\hat{X}}_{i}^{1:n}G^{⊗\nu }$8:  ${\hat{W}}_{i}\leftarrow {\hat{U}}_{i}^{1:n}\left[V_{W}\right]$9:  ${\hat{W}}_{i}^{\prime }\leftarrow {\hat{U}}_{i}^{1:n}\left[V_{W^{\prime }}\right]$10:**end for**


## 3. Preliminaries: Polarization of Sources with Vanishing Entropy Rate

Our code construction exploits recent results on polar codes that suggest how information-theoretic proofs exploiting source coding with side information and privacy amplification as primitives [16,17] may be converted into polar coding schemes by a suitable identification of polarization sets [11,18]. Specifically, the approach consists in recognizing that both primitives have counterparts based on polar codes, see Lemma 3 and Lemma 4 of [11], as well as [19,20]. Before we pursue a similar approach here, we must first extend Lemmas 3 and 4 of [11] to the case relevant for covert communications.

Formally, consider the sequences of positive numbers ${\left\{\alpha_{n}\right\}}_{n\in D}$ such that $\alpha_{n}\in \omega \left(\frac{1}{\sqrt{n}}\right)\cap o\left(1\right)$. For every n∈D, define the Bernoulli distribution $\Pi_{\alpha_{n}}$ over {0,1} as $\Pi_{\alpha_{n}}\left(1\right)=1-\Pi_{\alpha_{n}}\left(0\right)=\alpha_{n}$ and its associated product distribution

(5) $$\begin{array}{c} \Pi_{\alpha_{n}}^{⊗n}\left(x\right)=\prod_{i=1}^{n}\Pi_{\alpha_{n}}\left(x_{i}\right). \end{array}$$

Define the joint distribution of sequences in $X^{n}\times Y^{n}$

(6) $$\begin{array}{c} q_{X^{1:n}Y^{1:n}}\left(x,y\right)≜\Pi_{\alpha_{n}}^{⊗n}\left(x\right)W_{Y|X}^{⊗n}\left(y|x\right), \end{array}$$

with $W_{Y|X}$ defined in Section 2.2. In other words, for a fixed *n*, the process $X^{1:n}Y^{1:n}$ has a product distribution but the process ${\left\{X^{1:n}Y^{1:n}\right\}}_{n\in D}$ is not stationary and the entropy rate $\frac{1}{n}H\;\left(X^{1:n}|Y^{1:n}\right)$ vanishes. We refer to such a source as a “vanishing entropy rate source”. Assume now that the random vector $X^{1:n}\in X^{n}$ is transformed into $U^{1:n}=X^{1:n}G^{⊗\nu }$. For $\delta_{n}\in ]0,\frac{1}{2}[$, the set of high entropy bits is defined as

(7) $$H_{X|Y}\left(\delta_{n}\right)≜\left\{i\in 〚1,n〛:H\left(U_{i}|U^{1:i-1}Y^{1:n}\right)>\delta_{n}\right\},$$

and the set of very high entropy bits is defined as

(8) $$V_{X|Y}\left(\delta_{n}\right)≜\left\{i\in 〚1,n〛:H\left(U_{i}|U^{1:i-1}Y^{1:n}\right)>1-\delta_{n}\right\}$$

The following proposition shows that the sets $H_{X|Y}$ and $V_{X|Y}$ can still polarize for vanishing entropy rate sources.

**Proposition** **3.** (Fine polarization of vanishing entropy sources). *For any $\delta \in \;\left[0\;,\;\frac{1}{2}\right]$, set $\delta_{n}=2^{-n^{\delta }}$. For any ε∈[0,1−2δ], there exists $\kappa_{\delta ,\varepsilon }>0$, $A_{\delta ,\varepsilon }>0$ and $C_{\delta ,\varepsilon }$ such that for any vanishing entropy rate source $q_{X^{1:n}Y^{1:n}}\left(x,y\right)$ as in (6) and for any integer n∈D with $n>2^{C_{\delta ,\varepsilon }}$, we have*

(9) $$\begin{array}{ccc} 0 & \leq & \frac{|H_{X|Y}\left(\delta_{n}\right)\cap V_{X|Y}{\left(\delta_{n}\right)}^{c}|}{n}\leq \frac{A_{\delta ,\varepsilon }}{n^{\kappa_{\delta ,\varepsilon }\varepsilon }} \end{array}$$

(10) $$\begin{array}{ccc} \frac{1}{n}H\;\left(X^{1:n}|Y^{1:n}\right)-\delta_{n} & \leq & \frac{\left|H_{X|Y}\left(\delta_{n}\right)\right|}{n}\leq \frac{1}{n}H\;\left(X^{1:n}|Y^{1:n}\right)+\frac{A_{\delta ,\varepsilon }}{n^{\kappa_{\delta ,\varepsilon }\varepsilon }} \end{array}$$

(11) $$\begin{array}{ccc} \frac{1}{n}H\;\left(X^{1:n}|Y^{1:n}\right)-\frac{A_{\delta ,\varepsilon }}{n^{\kappa_{\delta ,\varepsilon }\varepsilon }} & \leq & \frac{\left|V_{X|Y}\left(\delta_{n}\right)\right|}{n}\leq \frac{1}{n}H\;\left(X^{1:n}|Y^{1:n}\right)+\delta_{n}. \end{array}$$

**Proof.**  The proof adapts the approach developed for finite length channel polarization [12] to source polarization. The idea is to first analyze a “rough” polarization to obtain a bound on the cardinality of the set of unpolarized sources, followed by a “fine” polarization to boost the polarization. Details require a careful adaptation but are otherwise similar to [12], and are therefore provided as supplementary material. ☐

For Proposition 3 to be meaningful, the relative size of the sets $H_{X|Y}\left(\delta_{n}\right)$ and $V_{X|Y}\left(\delta_{n}\right)$ in (10) and (11) should be asymptotically equivalent to the entropy rate $\frac{1}{n}H\;\left(X^{1:n}|Y^{1:n}\right)$. This is possible if $\frac{1}{n}H\;\left(X^{1:n}|Y^{1:n}\right)=\omega \left(\frac{1}{n^{\kappa_{\delta ,\varepsilon }\varepsilon }}\right)$, i.e., polarization happens “fast enough” and the relative number of unpolarized symbols in (9) decays faster than the entropy rate. Therefore, our result only ensures the polarization of vanishing entropy rate sources for values of $\alpha_{n}$ that do not decay too rapidly; specifically, we require $\alpha_{n}=\omega \left(\frac{1}{n^{\kappa_{\delta ,\varepsilon }\varepsilon }}\right)\cap o\left(1\right)$. Numerical analysis shows, for instance, that for δ=0.1 and ε=0.59, $\kappa_{\delta ,\varepsilon }\varepsilon \approx 6.53\times 10^{-3}$. Note that this falls short of $\frac{1}{\sqrt{n}}$, which would be required for the square-root-law of communication. Nevertheless, we are now able to extend Lemma 3 and Lemma 4 of [11] to the finite length regime, which forms the basis of our construction for covert communications.

**Lemma** **1.** (Source coding with side information). *Let $\delta \in \;[0\;,\;\frac{1}{2}]$, ε∈[0,1−2δ]; set $\delta_{n}=2^{-n^{\delta }}$ and let $\kappa_{\delta ,\varepsilon }>0$ be the constant identified by Proposition 3. Consider a vanishing entropy rate source $q_{X^{1:n}Y^{1:n}}$, as per (6) with $\alpha_{n}=\omega \left(\frac{1}{n^{\kappa_{\delta ,\varepsilon }\varepsilon }}\right)\cap o\left(1\right)$. For $X^{1:n}$ polarized as $U^{1:n}=X^{1:n}G^{⊗\nu }$, let $U^{1:n}\left[H_{X|Y}\left(\delta_{n}\right)\right]$ denote the high entropy bits of $U^{1:n}$. For every i∈〚1,n〛, sample ${\tilde{U}}^{1:n}$ from the distribution*

(12) $${\tilde{p}}_{U_{i}|U^{1:i-1}}\left({\tilde{u}}_{i}|{\tilde{u}}^{1:i-1}\right)≜\left\{\begin{array}{cc} 1\{{\tilde{u}}_{i}=u_{i}\} & \mathrm{if}\;i\in H_{X|Y}\left(\delta_{n}\right)\\ q_{U_{i}|U^{1:i-1}Y^{1:n}}\left({\tilde{u}}_{i}|{\tilde{u}}^{1:i-1}y\right) & \mathrm{if}\;i\in H_{X|Y}{\left(\delta_{n}\right)}^{c} \end{array}\right.$$

*and create $\tilde{x}=\tilde{u}G^{⊗\nu }$. Then ${\tilde{X}}^{1:n}\neq X^{1:n}=O(n\delta_{n})$.*

**Proof.**  See the proof of Lemma 3 in [11], using Proposition 3 instead of the standard polarization result. ☐

**Lemma** **2** (Privacy amplification). *Let $\delta \in \;[0\;,\;\frac{1}{2}]$, ε∈[0,1−2δ]; set $\delta_{n}=2^{-n^{\delta }}$ and let $\kappa_{\delta ,\varepsilon }>0$ be the constant identified by Proposition 3. Consider a vanishing entropy rate source $q_{X^{1:n}Y^{1:n}}$, as per (6) with $\alpha_{n}=\omega \left(\frac{1}{n^{\kappa_{\delta ,\varepsilon }\varepsilon }}\right)\cap o\left(1\right)$. For $X^{1:n}$ polarized as $U^{1:n}=X^{1:n}G^{⊗\nu }$, let $U^{1:n}\left[V_{X|Y}(\delta_{n})\right]$ denote the very high entropy bits of $U^{1:n}$. Denote by $q_{U^{1:n}\left[V_{X|Y}(\delta_{n})\right]Y^{1:n}}$ the joint distribution between $U^{1:n}\left[V_{X|Y}(\delta_{n})\right]$ and $Y^{1:n}$, and denote by $q_{U}$ the uniform distribution over $〚1,2^{\left|V_{X|Y}\right|}〛$. Then, $V\left(q_{U^{1:n}\left[V_{X|Y}(\delta_{n})\right]Y^{1:n}},q_{U}q_{Y^{1:n}}\right)=O\left(\sqrt{n\delta_{n}}\right)$.*

**Proof.**  See the proof of Lemma 4 in [11], using Proposition 3 instead of the standard polarization result. ☐

## 4. Polar Codes for Covert Communication

In this section, we describe our proposed polar-code based scheme for covert communication. After preliminaries regarding covert processes in Section 4.1, the algorithms used for encoding and decoding are described in Section 4.2, and their performance is analyzed in Section 4.3, Section 4.4 and Section 4.5.

### 4.1. Covert Process

Our code construction follows the idea put forward in [3,6], which suggests to have the code induce a “covert process” at the output of the adversary’s channel by leveraging the notion of channel resolvability [8], and to show that the covert process is itself indistinguishable from the product distribution $Q_{0}$.

Formally, consider any sequence of positive numbers ${\left\{\alpha_{n}\right\}}_{n\in D}$ such that $\alpha_{n}\in \omega \left(\frac{1}{\sqrt{n}}\right)\cap o\left(1\right)$. For every n∈D, recall the definition of the Bernoulli distribution $\Pi_{\alpha_{n}}$ over {0,1} as $\Pi_{\alpha_{n}}\left(1\right)=1-\Pi_{\alpha_{n}}\left(0\right)=\alpha_{n}$, and its associated product distribution $\Pi_{\alpha_{n}}^{⊗n}$; this distribution induces the mixture $Q_{\alpha_{n}}=\alpha_{n}Q_{1}+\left(1-\alpha_{n}\right)Q_{0}$ at the output of the channel $\left(X,W_{Z|X},Z\right)$, for which we also define the product distribution

(13) $$Q_{\alpha_{n}}^{⊗n}\left(z\right)=\prod_{i=1}^{n}Q_{\alpha_{n}}\left(z_{i}\right).$$

The “covert process” is the distribution $Q_{\alpha_{n}}^{N}\left(z\right)=E_{T}\left(Q_{\alpha_{n},T}^{N}\left(z\right)\right)$ where

(14) $$\begin{array}{c} Q_{\alpha_{n},t}^{N}\left(z\right)=\prod_{k=1}^{t-1}Q_{0}\left(z_{k}\right)\prod_{k=t}^{t+n-1}Q_{\alpha_{n}}\left(z_{k}\right)\prod_{k=t+n}^{N}Q_{0}\left(z_{k}\right) \end{array}$$

In other words, $Q_{\alpha_{n}}^{N}$ is the distribution at the output of the channel $\left(X,W_{Z|X},Z\right)$ obtained when randomizing the start index $T\in 〚1,N^{\prime }〛$ of a block of *n* consecutive bits sampled according to $\Pi_{\alpha_{n}}$. The name “covert process” is justified by the following lemma, which provides the scaling of the parameters $\alpha_{n}$ and *N* such that the distribution $Q_{\alpha_{n}}^{N}$ becomes asymptotically indistinguishable from the distribution $Q_{0}^{⊗N}$.

**Lemma** **3.** (adapted from Lemma 1 and Equation (25) in [6]). Consider sequences of positive numbers ${\left\{\alpha_{n}\right\}}_{n\in N^{*}}$, ${\left\{\beta_{n}\right\}}_{n\in N^{*}}$ such that $\alpha_{n}\in \omega \left(\frac{1}{\sqrt{n}}\right)\cap o\left(1\right)$, $\beta_{n}=o\left(1\right)$ as n goes to infinity. Let $N=\frac{2^{n\alpha_{n}^{2}}}{\beta_{n}\alpha_{n}^{2}}$. Then,

(15) $$D\;\left(Q_{\alpha_{n}}^{N}∥Q_{0}^{⊗N}\right)\leq O\left(\beta_{n}\right).$$

### 4.2. Encoding and Decoding Algorithms

Let n∈D be the length of the codeword window. We propose a scheme that operates over $b_{n}$ transmission windows of length *N*, where $b_{n}$ will be specified later. In every transmission window $i\in 〚1,b_{n}〛$:

1. Transmitter and receiver use a secret key $S_{i}^{\prime \prime }$ of logN bits to determine the position of the codeword window within the transmission window. Note that this secret key is not required in the random coding proof of [6], but is required here to maintain a low complexity at the decoder; fortunately, this change has negligible effect on the scaling of the key.
2. The content of each codeword window is obtained through a polar-code based scheme that ensures reliable decoding to the receiver and approximates the process $Q_{\alpha_{n}}^{⊗n}$ at the adversary’s output, which we describe next.

**Figure 3:**
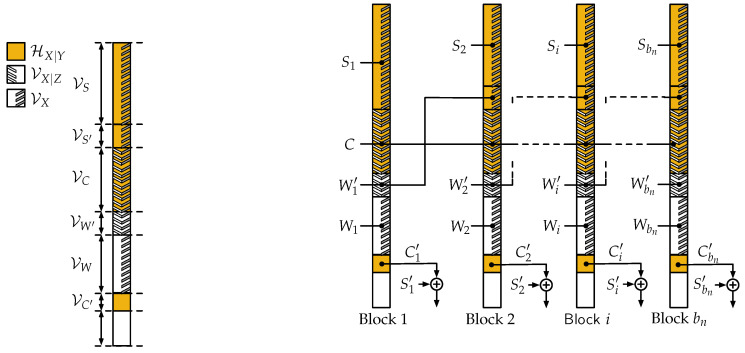
Illustration of polar coding scheme. (**a**) Sets used in polar coding scheme assuming $\left|H_{X|Y}^{c}\cap V_{X|Z}\right|\leq \left|H_{X|Y}\cap V_{X|Z}^{c}\cap V_{X}\right|$; (**b**) Chaining construction.

In the remainder of this section we fix $\delta \in ]0,\frac{1}{2}[$, ε∈]0,1−2δ[, $\delta_{n}≜2^{-n^{\delta }}$. We let $\kappa ≜\kappa_{\delta ,\varepsilon }\varepsilon$ and $A≜A_{\delta ,\varepsilon }$, where $\kappa_{\delta ,\varepsilon }$ and $A_{\delta ,\varepsilon }$ are the constants identified by Proposition 3. We consider sequences of positive numbers ${\left\{\alpha_{n}\right\}}_{n\in D}\in \omega \left(\frac{1}{n^{\kappa }}\right)\cap o\left(1\right)$, ${\left\{\beta_{n}\right\}}_{n\in D}\in \omega \left(2^{-\frac{n\alpha_{n}}{\log n}}\right)\cap o\left(\frac{1}{\log n}\right)$, a sequence of integers ${\left\{b_{n}\right\}}_{n\in D}\in \omega \left(\log n\right)\cap o\left(\frac{1}{\beta_{n}}\right)\cap o\left(n\right)$, and we set $N=\frac{2^{n\alpha_{n}^{2}}}{\beta_{n}\alpha_{n}^{2}}$. Finally, we consider a vanishing entropy rate source $q_{X^{1:n}Y^{1:n}Z^{1:n}}∼\Pi_{\alpha_{n}}^{⊗n}W_{Y|X}^{⊗n}W_{Z|X}^{⊗n}$ (the marginal $q_{Z^{1:n}}^{⊗n}$ is $Q_{\alpha_{n}}^{⊗n}$) and we define the sets

(16) $$\begin{array}{cc} V_{X}\left(\delta_{n}\right) & ≜\{j\in 〚1,n〛:\;H\left(U_{j}|U^{1:j-1}\right)>1-\delta_{n}\} \end{array}$$

(17) $$\begin{array}{cc} H_{X|Y}\left(\delta_{n}\right) & ≜\{j\in 〚1,n〛:\;H\left(U_{j}|U^{1:j-1}Y^{1:n}\right)>\delta_{n}\} \end{array}$$

(18) $$\begin{array}{cc} V_{X|Z}\left(\delta_{n}\right) & ≜\{j\in 〚1,n〛:\;H\left(U_{j}|U^{1:j-1}Z^{1:n}\right)>1-\delta_{n}\}, \end{array}$$

where all entropies should be computed based on $q_{X^{1:n}Y^{1:n}Z^{1:n}}$. To alleviate the notation, we drop the dependence on $\delta_{n}$ in the sets from now on, and write for instance $V_{X}$ in place of $V_{X}\left(\delta_{n}\right)$. We also write H(X) and H(X|Y) although these quantities should be understood for the independent and identically distributed (i.i.d.) random variables obtained as marginals of $q_{X^{1:n}Y^{1:n}Z^{1:n}}$. As illustrated in Figure 3a, the construction is based on the following sets:

- $V_{C}≜H_{X|Y}\cap V_{X|Z}$, which will contain uniformly distributed bits *C* representing the code;
- $V_{C^{\prime }}≜H_{X|Y}\cap V_{X}^{c}$, which will contain non-uniformly distributed bits $C^{\prime }$ computed from the other bits;
- $V_{W^{\prime }}$, the largest subset of $H_{X|Y}^{c}\cap V_{X|Z}$ such that $\left|V_{W^{\prime }}\right|\leq \left|H_{X|Y}\cap V_{X|Z}^{c}\cap V_{X}\right|$, which will contain uniformly distributed messages $W^{\prime }$;
- $V_{W}≜\left(H_{X|Y}^{c}\cap V_{X}\right)⧹V_{W^{\prime }}$, which will contain additional uniformly distributed messages *W*;
- $V_{S^{\prime }}$, any subset of $H_{X|Y}\cap V_{X|Z}^{c}\cap V_{X}$ such that $\left|V_{S^{\prime }}\right|=\left|V_{W^{\prime }}\right|$, which will use messages $W^{\prime }$ transmitted in the previous transmission window as a key;
- $V_{S}=\left(H_{X|Y}\cap V_{X|Z}^{c}\cap V_{X}\right)⧹V_{S^{\prime }}$, which will contain uniformly distributed secret key symbols *S*.

Alice’s encoder is formally provided in Algorithm 1 while Bob’s decoder is provided in Algorithm 2, but the chaining of the transmission windows over $b_{n}$ blocks is illustrated in Figure 3b and we discuss here the salient features of the algorithms. In every block $i\in 〚1,b_{n}〛$, a message $W_{i}$ is transmitted with the assistance of a secret key $S_{i}$ as expected from the model of Section 2.2. In addition, the chaining exploits the property that the bits in $V_{W^{\prime }}$ are held secret from Willie and can therefore be used as a secret key in the next block, which is formally proved in Section 4.5; this chaining allows us to transmit an additional message $W_{i}^{\prime }$ in every block, which is crucial to achieve the scalings of Proposition 2 as shown in Section 4.3. The chaining also relies on the secrecy of the bits in $V_{C}$, which allows us to reuse the same random bits *C* across all blocks. Finally, some bits of shared randomness $C_{i}^{\prime }$ must be transmitted secretly, covertly, and reliably to the receiver. As we show in Section 4.3, the number of such bits is negligible compared to the number of covert bits transmitted; we therefore ensure their secrecy by performing a one time pad $C_{i}^{\prime }⊕S_{i}^{\prime }$ with another secret key $S_{i}^{\prime }$, and we ensure reliability and covertness in a single additional block at the end, e.g., using the somewhat inefficient scheme of [1]. In the remainder, we will ignore this last block for simplicity and assume that $C_{i}^{\prime }⊕S_{i}^{\prime }$ is made available to the decoder “for free”.

Ultimately, the messages transmitted consist of the messages $W_{i}$ and $W_{i}^{\prime }$ transmitted in every block *i*; the keys required consist of the keys $S_{i}$, $S_{i}^{\prime }$, $S_{i}^{\prime \prime }$ used in every block *i*, as well as the bits *C*.

**Remark** **1.** The proposed chaining scheme could be further modified as follows. First, since the bits of C are secret from the perspective of Willie, they could be publicly disclosed and not counted as part of the secret keys, without compromising the performance. We have opted to count C as part of the key to make the analysis slightly more concise. Second, the bits of $C_{i}^{\prime }⊕S_{i}^{\prime }$ could be chained by sacrificing part of the message $W_{i}$; since their amount is negligible, this would again not affect performance. We have opted to avoid this chaining since a last transmission for the bits $C_{b_{n}}^{\prime }⊕S_{b_{n}}^{\prime }$ would be necessary anyway.

**Remark** **2.** Because of the stochastic encoding in Algorithm 1, our codes are neither linear codes nor cosets of linear codes. In that regard, calling our codes “polar codes” is a slight abuse of terminology but follows standard practice [11,18,20]. Strictly speaking, our codes are only “polarization-based”.

### 4.3. Analysis of Normalized Set Sizes

We start by analyzing the normalized set sizes of the proposed scheme. Specifically, we are interested in characterizing the asymptotic total number of message bits $\log M_{n}$ and total number of key bits $\log K_{n}$, normalized by $nb_{n}\alpha_{n}$.

Over $b_{n}$ transmission windows, the total number of message bits consists of those in $V_{W}$ and $V_{W^{\prime }}$ in every transmission window. Hence, for every n∈D, $\log M_{n}=b_{n}\left|V_{W}\right|+b_{n}\left|V_{W^{\prime }}\right|$. Similarly, the total number of key bits consists of those in $V_{S}$ (except for the first block which requires $V_{S}+V_{S^{\prime }}$), the bits for the one time pad in $V_{C^{\prime }}$, the bits required to identify the codeword window within the transmission window, and the bits in $V_{C}$, so that $\log K_{n}=b_{n}\left|V_{S}\right|+\left|V_{S^{\prime }}\right|+b_{n}\left|V_{C^{\prime }}\right|+b_{n}\log N+\left|V_{C}\right|$.

**Lemma** **4.** $$\begin{array}{c} \lim_{n\in D\to \infty }\frac{\log M_{n}}{nb_{n}\alpha_{n}}=D\;\left(P_{1}∥P_{0}\right). \end{array}$$

**Proof.**  By definition,

$$\begin{array}{c} \frac{\log M_{n}}{nb_{n}\alpha_{n}}=\frac{\left(\left|V_{W}\right|+\left|V_{W^{\prime }}\right|\right)}{n\alpha_{n}}=\frac{\left|H_{X|Y}^{c}\cap V_{X}\right|}{n\alpha_{n}}. \end{array}$$

Introducing $H_{X|Y}^{c}\subset V_{X|Y}^{c}$, we obtain

(19) $$\left|H_{X|Y}^{c}\cap V_{X}^{}\right|=\left|V_{X|Y}^{c}\cap V_{X}^{}\right|-\left|H_{X|Y}^{}\cap V_{X|Y}^{c}\cap V_{X}^{}\right|$$

Since, $V_{X|Y}^{}\subset V_{X}^{}$, we have $|V_{X|Y}^{c}\cap V_{X}^{}\left|=\right|V_{X}^{}\left|-\right|V_{X|Y}^{}|$ and, in addition, $0\leq |H_{X|Y}^{}\cap V_{X|Y}^{c}\cap V_{X}^{}\left|\leq \right|H_{X|Y}^{}\cap V_{X|Y}^{c}|$. Using Proposition 3 to bound $|V_{X}^{}|$, $|V_{X|Y}^{}|$, and $|H_{X|Y}^{}\cap V_{X|Y}^{c}|$, we obtain

(20) $$\begin{array}{cc} \frac{\log M_{n}}{nb_{n}\alpha_{n}} & \geq \frac{H\;\left(X\right)-H\;\left(X|Y\right)}{\alpha_{n}}-\frac{\delta_{n}}{\alpha_{n}}-2\frac{A}{n^{\kappa }\alpha_{n}}. \end{array}$$

Since $H\;\left(X\right)-H\;\left(X|Y\right)=I\;\left(X;Y\right)=\alpha_{n}D\;\left(P_{1}∥P_{0}\right)+o\left(\alpha_{n}\right)$ (Lemma 1, [3]), and remembering the choice of $\alpha_{n}$, $\delta_{n}$ earlier, we obtain the desired result (note that we use $\alpha_{n}\in \omega \left(\frac{1}{n^{\kappa }}\right)\cap o\left(1\right)$ here). ☐

**Lemma** **5.** $$\begin{array}{c} \lim_{n\in D\to \infty }\frac{\log K_{n}}{nb_{n}\alpha_{n}}={\left[D\;\left(Q_{1}∥Q_{0}\right)-D\;\left(P_{1}∥P_{0}\right)\right]}^{+}. \end{array}$$

**Proof.**  We first assume that $\left|H_{X|Y}^{c}\cap V_{X|Z}\right|\leq \left|H_{X|Y}\cap V_{X|Z}^{c}\cap V_{X}\right|$. By definition,

$$\begin{array}{cc} \frac{\log M_{n}+\log K_{n}}{nb_{n}\alpha_{n}} & =\frac{b_{n}\left|V_{W}^{}\right|+b_{n}\left|V_{S}^{}\right|+\left(b_{n}+1\right)\left|V_{S^{\prime }}^{}\right|+b_{n}\left|V_{C^{\prime }}\right|+b_{n}\log N+\left|V_{C}\right|}{nb_{n}\alpha_{n}}\\ & =\frac{\left|V_{W}^{}\right|+\left|V_{S}\right|+\left|V_{S^{\prime }}^{}\right|+\left|V_{C^{\prime }}^{}\right|}{n\alpha_{n}}+\frac{\left|V_{S^{\prime }}^{}\right|+\left|V_{C}^{}\right|}{nb_{n}\alpha_{n}}+\frac{\log N}{n\alpha_{n}}. \end{array}$$

We analyze the terms on the right hand side in order. First, since $V_{X|Z}\subset V_{X}$, we have $\left|V_{W}^{}\right|+\left|V_{S}\right|+\left|V_{S^{\prime }}^{}\right|=\left|V_{X|Z}^{c}\cap V_{X}\right|=\left|V_{X}^{}\right|-\left|V_{X|Z}^{}\right|$, and by Proposition 3 applied to the vanishing entropy rate sources $q_{X^{1:n}}$ and $q_{X^{1:n}Z^{1:n}}$

(21) $$\begin{array}{cc} \frac{\left|V_{W}^{}\right|+\left|V_{S}\right|+\left|V_{S^{\prime }}^{}\right|}{n\alpha_{n}} & \geq \frac{H(X)-H(X|Z)}{\alpha_{n}}-\frac{\delta_{n}}{\alpha_{n}}-\frac{A}{n^{\kappa }\alpha_{n}}. \end{array}$$

Since $H\;\left(X\right)-H\;\left(X|Z\right)=I\;\left(X;Z\right)=\alpha_{n}D\;\left(Q_{1}∥Q_{0}\right)+o\left(\alpha_{n}\right)$ (Lemma 1, [3]) and remembering the choice of $\alpha_{n}$, $\delta_{n}$ earlier, it follows that $\frac{\left|V_{W}^{}\right|+\left|V_{S}\right|+\left|V_{S^{\prime }}^{}\right|}{n\alpha_{n}}=D\;\left(Q_{1}∥Q_{0}\right)+o\left(1\right)$. This also implies that $\frac{\left|V_{S^{\prime }}^{}\right|}{nb_{n}\alpha_{n}}=o\left(1\right)$. Next, since $V_{X}^{c}\subset V_{X|Y}^{c}$, we have with Proposition 3 that

(22) $$\begin{array}{c} \frac{\left|V_{C^{\prime }}\right|}{n\alpha_{n}}\leq \frac{\left|H_{X|Y}\cap V_{X|Y}^{c}\right|}{n\alpha_{n}}\leq \frac{A}{n^{\kappa }\alpha_{n}}, \end{array}$$

which vanishes by definition of $\alpha_{n}$. Similarly, since $V_{C}\subset H_{X}$, Proposition 3 applied to the vanishing entropy rate source $q_{Z^{1:n}}$ ensures that

(23) $$\begin{array}{c} \frac{\left|V_{C}\right|}{nb_{n}\alpha_{n}}\leq \frac{H(X^{1:n})}{nb_{n}\alpha_{n}}+\frac{A}{n^{\kappa }b_{n}\alpha_{n}}=-\frac{\log\alpha_{n}}{b_{n}}-\frac{\left(1-\alpha_{n}\right)\log\left(1-\alpha_{n}\right)}{b_{n}\alpha_{n}}+\frac{A}{n^{\kappa }b_{n}\alpha_{n}}, \end{array}$$

which vanishes with our choice of $\alpha_{n}$ and $b_{n}$ (note that we use the condition $b_{n}=\omega \left(\log n\right)$ here). Finally, since $N=\frac{2^{n\alpha_{n}^{2}}}{\beta_{n}\alpha_{n}^{2}}$, we have

(24) $$\begin{array}{c} \frac{\log N}{n\alpha_{n}}=\alpha_{n}-\frac{\log\beta_{n}}{n\alpha_{n}}-\frac{2\log\alpha_{n}}{n\alpha_{n}}, \end{array}$$

which vanishes with the choice of $\alpha_{n}$, $\beta_{n}$ (note that we use the condition $\beta_{n}\in \omega \left(2^{-\frac{n\alpha_{n}}{\log n}}\right)$ here).

We finally assume that $\left|H_{X|Y}^{c}\cap V_{X|Z}\right|>\left|H_{X|Y}\cap V_{X|Z}^{c}\cap V_{X}\right|$, which is equivalent to assuming that $\left|V_{X}\cap H_{X|Y}^{c}\right|>\left|V_{X}\cap V_{X|Z}^{c}\right|$. With Proposition 3 and Lemma 1 of [3], this implies that $D\;\left(P_{1}∥P_{0}\right)>D\;\left(Q_{1}∥Q_{0}\right)+o\left(1\right)$. Since we have $V_{S}=\emptyset$ in this case, following the same steps as earlier we now obtain $\lim_{n\in D\to \infty }\frac{\log K_{n}}{nb_{n}\alpha_{n}}=0$, which is the desired result. ☐

### 4.4. Reliability Analysis

In this section, we prove that the proposed scheme ensures reliable communication. To avoid any confusion between the distribution induced by the algorithms and the underlying vanishing entropy rate source, we denote the distribution induced by Algorithm 1 by $\tilde{p}$; accordingly, all random variables generated according to this distribution have a tilde, e.g., $\tilde{X}$ has distribution ${\tilde{p}}_{X}$. The estimates obtained from Algorithm 2 are denoted with a hat, e.g., $\hat{X}$. Since the location of the transmission window is known to the legitimate receiver, it is sufficient to show that $\lim_{n\to \infty }P\left[{\hat{X}}_{1:b_{n}}^{1:n}\neq {\tilde{X}}_{1:b_{n}}^{1:n}\right]=0$. We proceed to prove this with a series of lemmas.

**Lemma** **6.** *For any transmission window $i\in 〚1,b_{n}〛$*

(25) $$D\left(q_{X^{1:n}Y^{1:n}}∥{\tilde{p}}_{X_{i}^{1:n}Y_{i}^{1:n}}\right)=D\left(q_{X^{1:n}}∥{\tilde{p}}_{X_{i}^{1:n}}\right)\leq \delta_{n}^{\left(1\right)}$$

*where $\delta_{n}^{\left(1\right)}≜n\delta_{n}$.*

**Proof.**  We have

(26) $$\begin{array}{cc} D\left(q_{X^{1:n}Y^{1:n}}∥{\tilde{p}}_{X_{i}^{1:n}Y_{i}^{1:n}}\right) & =D\left(q_{X^{1:n}}∥{\tilde{p}}_{X_{i}^{1:n}}\right)+E_{q_{X^{1:n}}}\left[D\left(q_{Y^{1:n}|X^{1:n}}∥{\tilde{p}}_{Y_{i}^{1:n}|X_{i}^{1:n}}\right)\right] \end{array}$$

(27) $$\begin{array}{cc} & =D\left(q_{X^{1:n}}∥{\tilde{p}}_{X_{i}^{1:n}}\right) \end{array}$$

(28) $$\begin{array}{cc} & =D\left(q_{U^{1:n}}∥{\tilde{p}}_{U_{i}^{1:n}}\right) \end{array}$$

(29) $$\begin{array}{cc} & =\sum_{j=1}^{n}E_{q_{U^{1:j-1}}}\left[D\left(q_{U^{j}|U^{1:j-1}}∥{\tilde{p}}_{U_{i}^{j}|U_{i}^{1:j-1}}\right)\right] \end{array}$$

(30) $$\begin{array}{cc} & =\sum_{j\in V_{X}}E_{q_{U^{1:j-1}}}\left[D\left(q_{U^{j}|U^{1:j-1}}∥{\tilde{p}}_{U_{i}^{j}|U_{i}^{1:j-1}}\right)\right] \end{array}$$

(31) $$\begin{array}{cc} & =\sum_{j\in V_{X}}\left(1-H\left(U^{j}|U^{1:j-1}\right)\right) \end{array}$$

(32) $$\begin{array}{cc} & \leq \left|V_{X}\right|\delta_{n} \end{array}$$

(33) $$\begin{array}{cc} & \leq n\delta_{n} \end{array}$$

where (26) comes from the chain rule of divergence, (27) comes from

(34) $$E_{q_{X^{1:n}}}\left[D\left(q_{Y^{1:n}|X^{1:n}}∥{\tilde{p}}_{Y_{i}^{1:n}|X_{i}^{1:n}}\right)\right]=E_{q_{X^{1:n}}}\left[D\left(W_{Y|X}^{⊗n}∥W_{Y|X}^{⊗n}\right)\right]=0$$

Equation (28) comes from the invertibility of $X^{1:n}=U^{1:n}G^{⊗\nu }$ and ${\tilde{X}}^{1:n}={\tilde{U}}^{1:n}G^{⊗\nu }$, (29) comes from the chain rule of divergence, (30) comes from the definition of the encoder for $j\in V_{X}^{c}$ in (4), (31) comes from the uniformity of the symbols in $V_{X}$, (32) comes from the definition of $V_{X}$. ☐

**Lemma** **7.** *For any transmission window $i\in 〚1,b_{n}〛$, define the event*

(35) $$E_{i}≜\left\{{\hat{X}}_{i}^{1:n}\neq {\tilde{X}}_{i}^{1:n}\right\}$$

*Then*,

(36) $$P\left[E_{i}|E_{i-1}^{c}\right]=P\left[{\hat{X}}_{i}^{1:n}\neq {\tilde{X}}_{i}^{1:n}|{\hat{X}}_{i-1}^{1:n}={\tilde{X}}_{i-1}^{1:n}\right]\leq \delta_{n}^{\left(2\right)}$$

*where $\delta_{n}^{\left(2\right)}=O\left(n^{1/2}\delta_{n}^{1/2}\right)$*.

**Proof.**  For $i\in 〚1,b_{n}〛$, define the event that the sequence produced by the polar encoder differs from the actual one:

(37) $$E_{i}^{\left(XY\right)}≜\left\{\left({\tilde{X}}_{i}^{1:n},{\tilde{Y}}_{i}^{1:n}\right)\neq \left(X^{1:n},Y^{1:n}\right)\right\}$$

and define an optimal coupling such that

(38) $$P\left[E_{i}^{\left(XY\right)}\right]=V\left(q_{X^{1:n}Y^{1:n}},{\tilde{p}}_{X_{i}^{1:n}Y_{i}^{1:n}}\right)\leq \sqrt{D\left(q_{X^{1:n}Y^{1:n}}∥{\tilde{p}}_{X_{i}^{1:n}Y_{i}^{1:n}}\right)}\leq \sqrt{\delta_{n}^{\left(1\right)}}$$

by Pinsker’s inequality and Lemma 6. Then, we have

(39) $$\begin{array}{cc} P\left[E_{i}|E_{i-1}^{c}\right] & =P\left[E_{i}|E_{i}^{(XY)c}\cap E_{i-1}^{c}\right]P\left[E_{i}^{(XY)c}\right]+P\left[E_{i}|E_{i}^{\left(XY\right)}\cap E_{i-1}^{c}\right]P\left[E_{i}^{\left(XY\right)}\right] \end{array}$$

(40) $$\begin{array}{c} \leq P\left[E_{i}|E_{i}^{(XY)c}\cap E_{i-1}^{c}\right]+P\left[E_{i}^{\left(XY\right)}\right] \end{array}$$

(41) $$\begin{array}{c} \leq O\left(n\delta_{n}\right)+\sqrt{\delta_{n}^{\left(1\right)}} \end{array}$$

where (39) comes from the law of total probabilities, (40) from $P\left[E_{i}^{(XY)c}\right]\leq 1$, $P\left[E_{i}|E_{i}^{\left(XY\right)}\cap E_{i-1}^{c}\right]\leq 1$, and (41) from Lemma 1 and the optimal coupling. ☐

**Lemma** **8.** *We have*

(42) $$P\left[{\hat{X}}_{1:b_{n}}^{1:n}\neq {\tilde{X}}_{1:b_{n}}^{1:n}\right]\leq \delta_{n}^{\left(3\right)}$$

*where $\delta_{n}^{\left(3\right)}=O\left(b_{n}n^{1/2}\delta_{n}^{1/2}\right)$.*

**Proof.**  We have the following partition

(43) $$\overset{b_{n}}{\underset{i=1}{⋃}}E_{i}=\overset{b_{n}}{\underset{i=1}{⋃}}\left(E_{i}\cap {\left(\overset{i-1}{\underset{j=1}{⋃}}E_{j}\right)}^{c}\right).$$

Thus,

(44) $$\begin{array}{cc} P\left[{\hat{X}}_{1:b_{n}}^{1:n}\neq {\tilde{X}}_{1:b_{n}}^{1:n}\right]=P\left[\overset{b_{n}}{\underset{i=1}{⋃}}E_{i}\right] & =P\left[\overset{b_{n}}{\underset{i=1}{⋃}}E_{i}\cap {\left(\overset{i-1}{\underset{j=1}{⋃}}E_{j}\right)}^{c}\right] \end{array}$$

(45) $$\begin{array}{c} =\sum_{i=1}^{b_{n}}P\left[E_{i}\cap {\left(\overset{i-1}{\underset{j=1}{⋃}}E_{j}\right)}^{c}\right] \end{array}$$

(46) $$\begin{array}{c} \leq \sum_{i=1}^{b_{n}}P\left[E_{i}\cap E_{i-1}^{c}\right] \end{array}$$

(47) $$\begin{array}{c} =\sum_{i=1}^{b_{n}}P\left[E_{i}|E_{i-1}^{c}\right]P\left[E_{i-1}^{c}\right] \end{array}$$

(48) $$\begin{array}{c} \leq \sum_{i=1}^{b_{n}}P\left[E_{i}|E_{i-1}^{c}\right] \end{array}$$

(49) $$\begin{array}{c} \leq O\left(b_{n}n\delta_{n}\right)+b_{n}\sqrt{\delta_{n}^{\left(1\right)}} \end{array}$$

where (45) comes from the probability of the partition, (46) from $E_{i-1}\subseteq \overset{i-1}{\underset{j=1}{⋃}}E_{j}$, (47) from the definition of conditional probability, (48) from $P\left[E_{i-1}^{c}\right]\leq 1$, and (49) from Lemma 7. The choice $b_{n}=o\left(n\right)$ ensures that $P\left[{\hat{X}}_{1:b_{n}}^{1:n}\neq {\tilde{X}}_{1:b_{n}}^{1:n}\right]$ vanishes. ☐

### 4.5. Covertness Analysis

In this section, we prove that the proposed scheme is covert in the sense that $\lim_{n\in D\to \infty }V\;\left({\tilde{p}}_{Z_{1:b_{n}}^{1:n}}∥q_{Z_{1:b_{n}}^{1:n}}\right)=0$, where $q_{Z_{1:b_{n}}^{1:n}}\left(z_{1},\ldots ,z_{b_{n}}\right)≜\prod_{i=1}^{b_{n}}Q_{\alpha_{n}}^{⊗n}\left(z_{i}\right)$.

**Lemma** **9.** *For any transmission window $i\in 〚1,b_{n}〛$*

(50) $$\begin{array}{cc} D\left(q_{X^{1:n}Z^{1:n}}∥{\tilde{p}}_{X_{i}^{1:n}Z_{i}^{1:n}}\right) & =D\left(q_{X^{1:n}}∥{\tilde{p}}_{X_{i}^{1:n}}\right)\leq \delta_{n}^{\left(1\right)} \end{array}$$

(51) $$\begin{array}{cc} V\left({\tilde{p}}_{Z_{i}^{1:n}},q_{Z^{1:n}}\right) & \leq V\left({\tilde{p}}_{X_{i}^{1:n}Z_{i}^{1:n}},q_{X^{1:n}Z^{1:n}}\right)\leq \delta_{n}^{\left(4\right)} \end{array}$$

*where $\delta_{n}^{\left(1\right)}≜n\delta_{n}$ and $\delta_{n}^{\left(4\right)}=\sqrt{n\delta_{n}}$.*

**Proof.**  The proof of the divergence inequality is identical to Lemma 6. The proof of the total variation distance inequality follows from

(52) $$\begin{array}{cc} V\left({\tilde{p}}_{Z_{i}^{1:n}},q_{Z^{1:n}}\right) & \leq V\left({\tilde{p}}_{X_{i}^{1:n}Z_{i}^{1:n}},q_{X^{1:n}Z^{1:n}}\right) \end{array}$$

(53) $$\begin{array}{c} \leq \sqrt{D\left(q_{X^{1:n}Z^{1:n}}∥{\tilde{p}}_{X_{i}^{1:n}Z_{i}^{1:n}}\right)} \end{array}$$

(54) $$\begin{array}{c} \leq \sqrt{\delta_{n}^{\left(1\right)}}=\sqrt{n\delta_{n}} \end{array}$$

where (52) comes from the total variation of marginal distributions, (53) from Pinsker’s inequality, and (54) from the previous inequality. ☐

**Lemma** **10.** *For $i\in 〚1,b_{n}〛$,*

(55) $$\begin{array}{c} I\;\left({\tilde{Z}}_{i}^{1:n};CW_{i}^{\prime }\right)\leq \delta_{n}^{\left(5\right)} \end{array}$$

*where $\delta_{n}^{\left(5\right)}=n\delta_{n}+2\delta_{n}^{\left(4\right)}\left[n\left(1+2\log|Z|\right)-2\log2\delta_{n}^{\left(4\right)}\right]$.*

**Proof.**  Let $i\in 〚2,b_{n}〛$.

(56) $$\begin{array}{c} H\left(\left.U^{1:n}\left[V_{X|Z}\right]\right|Z^{1:n}\right)-H\left(\left.{\tilde{U}}_{i}^{1:n}\left[V_{X|Z}\right]\right|{\tilde{Z}}_{i}^{1:n}\right) \end{array}$$

(57) $$\begin{array}{c} \;=H\left(U^{1:n}\left[V_{X|Z}\right],Z^{1:n}\right)-H\left({\tilde{U}}_{i}^{1:n}\left[V_{X|Z}\right],{\tilde{Z}}_{i}^{1:n}\right)+H\left({\tilde{Z}}_{i}^{1:n}\right)-H\left(Z^{1:n}\right) \end{array}$$

(58) $$\begin{array}{c} \;\leq 2V\left({\tilde{p}}_{U_{i}^{1:n}\left[V_{X|Z}\right],Z_{i}^{1:n}},q_{U^{1:n}\left[V_{X|Z}\right],Z^{1:n}}\right)\left[n\log\left(|X||Z|\right)-\log2V\left({\tilde{p}}_{U_{i}^{1:n}\left[V_{X|Z}\right],Z_{i}^{1:n}},q_{U^{1:n}\left[V_{X|Z}\right],Z^{1:n}}\right)\right] \end{array}$$

(59) $$\begin{array}{c} \;\;+2V\left({\tilde{p}}_{Z_{i}^{1:n}},q_{Z^{1:n}}\right)\left[n\log|Z|-\log2V\left({\tilde{p}}_{Z_{i}^{1:n}},q_{Z^{1:n}}\right)\right] \end{array}$$

(60) $$\begin{array}{c} \;\leq 2V\left({\tilde{p}}_{X_{i}^{1:n}Z_{i}^{1:n}},q_{X^{1:n}Z^{1:n}}\right)\left[n\log\left(|X||Z|\right)-\log2V\left({\tilde{p}}_{X_{i}^{1:n},Z_{i}^{1:n}},q_{X^{1:n},Z^{1:n}}\right)\right] \end{array}$$

(61) $$\begin{array}{c} \;\;+2V\left({\tilde{p}}_{Z_{i}^{1:n}},q_{Z^{1:n}}\right)\left[n\log|Z|-\log2V\left({\tilde{p}}_{Z_{i}^{1:n}},q_{Z^{1:n}}\right)\right] \end{array}$$

(62) $$\begin{array}{c} \;\leq 2\delta_{n}^{\left(4\right)}\left[n\log\left(|X||Z|\right)-\log2\delta_{n}^{\left(4\right)}\right]+2\delta_{n}^{\left(4\right)}\left[n\log|Z|-\log2\delta_{n}^{\left(4\right)}\right] \end{array}$$

(63) $$\begin{array}{c} \;=2\delta_{n}^{\left(4\right)}\left[n\left(1+2\log|Z|\right)-2\log2\delta_{n}^{\left(4\right)}\right] \end{array}$$

(64) $$\begin{array}{c} \;≜\delta_{n}^{\left(XZ\right)} \end{array}$$

where (57) comes from the chain rule of entropy, (58) and (59) from Lemma 2.7 of [21] with *n* large enough, (60) and (61) from the total variation of marginal distributions, the invertibility of $X^{1:n}=U^{1:n}G^{⊗\nu }$ and ${\tilde{X}}^{1:n}={\tilde{U}}^{1:n}G^{⊗\nu }$ and that the function x↦x(1−logx) is monotonically increasing, and (62) from Lemma 9. Hence for $i\in 〚2,b_{n}〛$,

(65) $$\begin{array}{cc} I\;\left({\tilde{Z}}_{i}^{1:n};CW_{i}^{\prime }\right) & \leq I\;\left({\tilde{Z}}_{i}^{1:n};{\tilde{U}}_{i}^{1:n}\left[V_{X|Z}\right]\right) \end{array}$$

(66) $$\begin{array}{c} =H\left({\tilde{U}}_{i}^{1:n}\left[V_{X|Z}\right]\right)-H\left({\tilde{U}}_{i}^{1:n}\left[V_{X|Z}\right]|{\tilde{Z}}_{i}^{1:n}\right) \end{array}$$

(67) $$\begin{array}{c} =|V_{X|Z}|-H\left({\tilde{U}}_{i}^{1:n}\left[V_{X|Z}\right]|{\tilde{Z}}_{i}^{1:n}\right) \end{array}$$

(68) $$\begin{array}{c} \leq |V_{X|Z}|-H\left(U^{1:n}\left[V_{X|Z}\right]|Z^{1:n}\right)+\delta_{n}^{\left(XZ\right)} \end{array}$$

(69) $$\begin{array}{c} \leq |V_{X|Z}|-\sum_{j\in V_{X|Z}}H\left(U^{j}|U^{1:j-1}Z^{1:n}\right)+\delta_{n}^{\left(XZ\right)} \end{array}$$

(70) $$\begin{array}{c} \leq |V_{X|Z}\left|-\right|V_{X|Z}|\left(1-\delta_{n}\right)+\delta_{n}^{\left(XZ\right)} \end{array}$$

(71) $$\begin{array}{c} \leq n\delta_{n}+\delta_{n}^{\left(XZ\right)} \end{array}$$

where (66) come the definition of mutual information, (65) and (70) from the definition of the set $V_{X|Z}$, (67) from the uniformity of symbols in $V_{X|Z}$, (68) comes from (64), and (69) from the chain rule of entropy and conditioning. ☐

**Lemma** **11.** *The outputs of all blocks are asymptotically independent in the sense that*

(72) $$V\left({\tilde{p}}_{Z_{1:b_{n}}^{1:n}},\prod_{i=1}^{b_{n}}{\tilde{p}}_{Z_{i}^{1:n}}\right)\leq b_{n}\sqrt{\delta_{n}^{\left(5\right)}}.$$

**Proof.**  We have

(73) $$\begin{array}{cc} V\left({\tilde{p}}_{Z_{1:b_{n}}^{1:n}},\prod_{i=1}^{b_{n}}{\tilde{p}}_{Z_{i}^{1:n}}\right) & \leq \sum_{i=1}^{b_{n}}E_{{\tilde{p}}_{Z_{1:i-1}^{1:n}}}\left[V\left({\tilde{p}}_{Z_{i}^{1:n}|Z_{1:i-1}^{1:n}},{\tilde{p}}_{Z_{i}^{1:n}}\right)\right] \end{array}$$

(74) $$\begin{array}{c} =\sum_{i=1}^{b_{n}}V\left({\tilde{p}}_{Z_{1:i}^{1:n}},{\tilde{p}}_{Z_{1:i-1}^{1:n}}{\tilde{p}}_{Z_{i}^{1:n}}\right) \end{array}$$

(75) $$\begin{array}{c} \leq \sum_{i=1}^{b_{n}}V\left({\tilde{p}}_{Z_{1:i}^{1:n}CW_{i}^{\prime }},{\tilde{p}}_{Z_{1:i-1}^{1:n}CW_{i}^{\prime }}{\tilde{p}}_{Z_{i}^{1:n}}\right) \end{array}$$

(76) $$\begin{array}{c} \leq \sum_{i=1}^{b_{n}}\sqrt{D\;\left({\tilde{p}}_{Z_{1:i}^{1:n}CW_{i}^{\prime }}∥{\tilde{p}}_{Z_{1:i-1}^{1:n}CW_{i}^{\prime }}{\tilde{p}}_{Z_{i}^{1:n}}\right)}. \end{array}$$

where (73) follows from the chain rule of total variation. Note that

(77) $$\begin{array}{cc} D\;\left({\tilde{p}}_{Z_{1:i}^{1:n}CW_{i}^{\prime }}∥{\tilde{p}}_{Z_{1:i-1}^{1:n}CW_{i}^{\prime }}{\tilde{p}}_{Z_{i}^{1:n}}\right) & =I\;\left({\tilde{Z}}_{1:i-1}CW_{i}^{\prime };{\tilde{Z}}_{i}^{1:n}\right) \end{array}$$

(78) $$\begin{array}{c} =I\;\left(CW_{i}^{\prime };{\tilde{Z}}_{i}^{1:n}\right)+I\;\left({\tilde{Z}}_{1:i-1};{\tilde{Z}}_{i}^{1:n}|CW_{i}^{\prime }\right) \end{array}$$

(79) $$\begin{array}{c} =I\;\left(CW_{i}^{\prime };{\tilde{Z}}_{i}^{1:n}\right), \end{array}$$

where we have used the Markov chain $Z_{1:i-1}^{1:n}-CW_{i}^{\prime }-Z_{i}^{1:n}$. The result then follows by Lemma 10. ☐

**Lemma** **12.** *We have*

(80) $$V\left({\tilde{p}}_{Z_{1:b_{n}}^{1:n}},q_{Z_{1:b_{n}}^{1:n}}\right)\leq \delta_{n}^{\left(6\right)}$$

*where $\delta_{n}^{\left(6\right)}=b_{n}\left(\sqrt{\delta_{n}^{\left(5\right)}}+\delta_{n}^{\left(4\right)}\right)$.*

**Proof.**  We have

(81) $$\begin{array}{cc} V\left({\tilde{p}}_{Z_{1:b_{n}}^{1:n}},q_{Z_{1:b_{n}}^{1:n}}\right) & =V\left({\tilde{p}}_{Z_{1:b_{n}}^{1:n}},\prod_{i=1}^{b_{n}}q_{Z^{1:n}}\right) \end{array}$$

(82) $$\begin{array}{c} \leq V\left({\tilde{p}}_{Z_{1:b_{n}}^{1:n}},\prod_{i=1}^{b_{n}}{\tilde{p}}_{Z_{i}^{1:n}}\right)+V\left(\prod_{i=1}^{b_{n}}{\tilde{p}}_{Z_{i}^{1:n}},\prod_{i=1}^{b_{n}}q_{Z^{1:n}}\right) \end{array}$$

(83) $$\begin{array}{c} \leq V\left({\tilde{p}}_{Z_{1:b_{n}}^{1:n}},\prod_{i=1}^{b_{n}}{\tilde{p}}_{Z_{i}^{1:n}}\right)+\sum_{i=1}^{b_{n}}V\left({\tilde{p}}_{Z_{i}^{1:n}},q_{Z^{1:n}}\right) \end{array}$$

(84) $$\begin{array}{c} \leq b_{n}\sqrt{\delta_{n}^{\left(5\right)}}+b_{n}\delta_{n}^{\left(4\right)} \end{array}$$

where we have used the results of Lemmas 9 and 11. ☐

We finally conclude the proof of covertness as follows. We let ${\left\{T_{i}\right\}}_{i=1}^{b_{n}}$ be the independent uniform random variables denoting the choice of the start time in each of the chained $b_{n}$ transmission windows. Note that the distribution ${\hat{Q}}^{b_{n}N}$ induced by the code may be written as ${\hat{Q}}^{b_{n}N}\left(z\right)=E_{T_{1},\cdots ,T_{b_{n}}}\left({\hat{Q}}_{T_{1},\cdots ,T_{n}}^{b_{n}N}\left(z\right)\right)$ where for $\left(t_{1},\cdots ,t_{b_{n}}\right)\in 〚1,N^{\prime }{〛}^{b_{n}}$

(85) $$\begin{array}{c} {\hat{Q}}_{t_{1},\cdots ,t_{n}}^{b_{n}N}\left(z\right)={\hat{Q}}^{b_{n}n}\left(z_{t}\right)\prod_{i=1}^{b_{n}}\left(\prod_{k=1}^{t_{i}-1}Q_{0}\left(z_{(i-1)N+k}\right)\prod_{k=t_{i}+n}^{N}Q_{0}\left(z_{(i-1)N+k}\right)\right) \end{array}$$

and $z_{t}$ contains the components ${\left\{z_{\left(i-1\right)N+t_{i}-1+k}\right\}}_{i\in 〚1,b_{N}〛,k\in 〚1,n〛}$ corresponding to the positions where a code is used in every transmission window. This formulation allows us to isolate the distribution ${\hat{Q}}^{b_{n}n}\left(z_{t}\right)$, which corresponds to the chained coded blocks of length *n*. We also define the process $Q_{\alpha_{n}}^{b_{n}N}$ as

(86) $$\begin{array}{c} Q_{\alpha_{n}}^{b_{n}N}\left(z\right)≜E_{T_{1},\cdots ,T_{b_{n}}}\left(\prod_{i=1}^{b_{n}}Q_{\alpha_{n},T_{i}}^{N}\left(z_{i}\right)\right)=\prod_{i=1}^{b_{n}}E_{T_{i}}\left(Q_{\alpha_{n},T_{i}}^{N}\left(z_{i}\right)\right), \end{array}$$

where $z=(z_{1},\ldots ,z_{b_{n}})$. We then bound $V\;\left({\hat{Q}}^{b_{n}N},Q_{0}^{⊗b_{n}N}\right)$ as follows.

(87) $$\begin{array}{cc} V\;\left({\hat{Q}}^{b_{n}N},Q_{0}^{⊗b_{n}N}\right) & \overset{\left(a\right)}{\leq }V\;\left({\hat{Q}}^{b_{n}N},Q_{\alpha_{n}}^{b_{n}N}\right)+V\;\left(Q_{\alpha_{n}}^{b_{n}N},Q_{0}^{⊗b_{n}N}\right) \end{array}$$

(88) $$\begin{array}{c} \overset{\left(b\right)}{\leq }V\;\left(E_{T_{1},\cdots ,T_{b_{n}}}\left({\hat{Q}}_{T_{1},\cdots ,T_{n}}^{b_{n}N}\right),E_{T_{1},\cdots ,T_{b_{n}}}\left(\prod_{i=1}^{b_{n}}Q_{\alpha_{n},T_{i}}^{N}\right)\right)+\sqrt{b_{n}D\;\left(Q_{\alpha_{n}}^{N}∥Q_{0}^{⊗N}\right)} \end{array}$$

(89) $$\begin{array}{c} \overset{\left(c\right)}{\leq }E_{T_{1},\cdots ,T_{b_{n}}}\left(V\;\left(Q_{T_{1},\cdots ,T_{n}}^{b_{n}N}\right),\prod_{i=1}^{b_{n}}Q_{\alpha_{n},T_{i}}^{N}\right)+O\left(\sqrt{b_{n}\beta_{n}}\right) \end{array}$$

(90) $$\begin{array}{c} \overset{\left(d\right)}{=}V\;\left({\hat{Q}}^{b_{n}n},Q_{\alpha_{n}}^{⊗b_{n}n}\right)+O\left(\sqrt{b_{n}\beta_{n}}\right) \end{array}$$

(91) $$\begin{array}{c} \overset{\left(e\right)}{=}V\;\left({\tilde{p}}_{Z_{1:b_{n}}^{1:n}},q_{Z_{1:b_{n}}^{1:n}}\right)+O\left(\sqrt{b_{n}\beta_{n}}\right) \end{array}$$

(92) $$\begin{array}{c} \overset{\left(f\right)}{=}\delta_{n}^{\left(6\right)}+O\left(\sqrt{b_{n}\beta_{n}}\right), \end{array}$$

where (a) follows by the triangle inequality; (b) follows from the definition of ${\hat{Q}}^{b_{n}N}$ and $Q_{\alpha_{n}}^{b_{n}N}$, Pinsker’s inequality, and the product form of $Q_{\alpha_{n}}^{b_{n}N}$ and $Q_{0}^{⊗b_{n}N}$ over the $b_{n}$ blocks; (c) follows from the convexity of total variation distance and Lemma 3; (d) follows from the definition of $V\;\left(Q_{T_{1},\cdots ,T_{n}}^{b_{n}N}\right)$ and $\prod_{i=1}^{b_{n}}Q_{\alpha_{n},T_{i}}^{N}$; (e) follows by substituting the notation used in the analysis of the chained scheme; (f) follows from Lemma 12. With our choice of $\beta_{n}$, $b_{n}$, and $\delta_{n}$, note that $\lim_{n\to \infty }\delta_{n}^{\left(6\right)}=0$ and $\lim_{n\to \infty }b_{n}\beta_{n}=0$, hence establishing covertness (note that we use the condition $b_{n}\in o\left(\frac{1}{\beta_{n}}\right)$ here).

## 5. Conclusions

In this paper, we have proposed a coding scheme for covert communication based on polar codes. Although our scheme offers a first explicit solution of covert communication in a non-trivial regime, its performance is still far from that of random codes. The proven speed of polarization severely limits the rate at which the average weight of codewords can decay, and in particular we cannot approach the average codeword weight on the order of $\sqrt{n}$ required by the square root law. We have circumvented this issue by hiding the transmission window within a larger window as in [5,6], and at least in the regime for which our proofs hold, the proposed scheme achieves the best known rates. Several extensions and improvements are currently under investigation, particularly the refinement of Proposition 3 to improve the constant κ and the use of non-binary polar codes in conjunction with pulse-position modulation [22].

## Supplementary Materials

The following are available online at www.mdpi.com/1099-4300/20/1/3/s1.
