# Supplementary material for: Polar Codes for Covert Communications over Asynchronous Discrete Memoryless Channels
Source: Entropy (Basel). 2017 Dec 22;20(1):3. doi: 10.3390/e20010003 (PMC7512237; doi:10.3390/e20010003)
Supplement: Supplementary file 1 [file entropy-20-00003-s001.pdf]

# Supplementary material for Proposition 3.1

## 1 Notations and preliminaries

The functions log and ln denote respectively the base 2 and the natural logarithms. By convention, $0\log 0 = 0 = 0\ln 0$. For $x \in \mathbb{R}$, $\lfloor x \rfloor$ (resp. $\lceil x \rceil$) denotes the greatest (resp. smallest) integer not greater (resp. not smaller) than $x$. For integers $a \leq b$, $[\![a\,,\,b]\!]$ denotes the set of integers between $a$ and $b$, bounds included. Let $\mathbb{D} = \{2^\mu \,:\, \mu \in \mathbb{N}\}$.

Let $(X,Y) \simeq P_{X,Y}$ be an arbitrary pair of random variables over $\mathcal{B} \times \mathcal{Y}$ with $\mathcal{B} = \{0,\,1\}$ and $\mathcal{Y}$ an arbitrary countable set. We regard $(X,Y)$ as a memoryless source $S$, with $X$ as the part to be compressed and $Y$ in the role of "side-information" about $X$. We consider a sequence $S = \{(X_i,Y_i) \,:\, i \in \mathbb{N}^*\}$ of independent drawings from $(X,Y)$ – which can be interpreted as a representation of the source $S$ – and we introduce the two transformations $^-$ and $^+$ applied to the source $S$ and defined by

$$
\begin{aligned}
S^- &= \left\{\left(X_{2i-1} \oplus X_{2i}, (Y_{2i-1}, Y_{2i})\right) \,:\, i \in \mathbb{N}^*\right\} && (1)\\
S^+ &= \left\{\left(X_{2i}, (Y_{2i-1}, Y_{2i}, X_{2i-1} \oplus X_{2i})\right) \,:\, i \in \mathbb{N}^*\right\}. && (2)
\end{aligned}
$$

With these notations, $S^-$ (resp. $S^+$) is the memoryless source that takes its values in $\mathcal{B} \times (\mathcal{Y}^2)$ (resp. in $\mathcal{B} \times (\mathcal{Y}^2 \times \mathcal{B})$), with $X_1 \oplus X_2$ (resp. $X_2$) as the part to be compressed and $(Y_1,Y_2)$ (resp. $(Y_1,Y_2,X_1 \oplus X_2)$) in the role of "side-information".

The process that constructs $S^-$ and $S^+$ from $S$ can be written $S_0^{(0)} = S$,

$$
S_1^{(0)} = \left(S_0^{(0)}\right)^- = S^- \qquad \text{and} \qquad S_1^{(1)} = \left(S_0^{(0)}\right)^+ = S^+. \tag{3}
$$

Applied recursively, this process leads to the sequence of memoryless sources $(S_\mu^{(i)})_{\mu\in\mathbb{N}, i\in[\![0\,,\,2^\mu-1]\!]}$, where $S_\mu^{(i)}$ takes its values in a set $\mathcal{B} \times \left(\mathcal{Y}^{2^\mu} \times \mathcal{B}^{K(i)}\right)$ with $K(i) \in [\![0\,,\,2^\mu - 1]\!]$ and is defined by

$$
S_{\mu+1}^{(i)} = \begin{cases} \left(S_\mu^{(\lfloor i/2 \rfloor)}\right)^- & \text{if } i \text{ is even} \\ \left(S_\mu^{(\lfloor i/2 \rfloor)}\right)^+ & \text{if } i \text{ is odd.} \end{cases} \tag{4}
$$

Let us introduce the sources' conditional entropies expressed in bits:

$$
\begin{aligned}
H(S) &= \mathbb{H}(X_1 \,|\, Y_1) = \mathbb{H}(X_2 \,|\, Y_2), && (5)\\
H(S^-) &= \mathbb{H}(X_1 \oplus X_2 \,|\, Y_1,\, Y_2), && (6)\\
H(S^+) &= \mathbb{H}(X_2 \,|\, Y_1,\, Y_2,\, X_1 \oplus X_2). && (7)
\end{aligned}
$$

For any $m \in \mathbb{D}$ ($m = 2^\mu$ with $\mu \in \mathbb{N}$) and for any $\theta \in \left]0\,,\,\frac{1}{2}\right]$, let

$$
\begin{aligned}
\mathcal{H}_{X|Y} &= \mathcal{H}_{X|Y}(\theta) = \mathcal{H}_{X|Y}^{(m)}(\theta) = \left\{i \in [\![0\,,\,m-1]\!] \,:\, H\left(S_\mu^{(i)}\right) > \theta\right\} && (8)\\
\mathcal{V}_{X|Y} &= \mathcal{V}_{X|Y}(\theta) = \mathcal{V}_{X|Y}^{(m)}(\theta) = \left\{i \in [\![0\,,\,m-1]\!] \,:\, H\left(S_\mu^{(i)}\right) > 1-\theta\right\}. && (9)
\end{aligned}
$$

For any memoryless source $S = (X,Y) \simeq P_{X,Y}$, we introduce its Bhattacharyya parameter:

$$
\begin{aligned}
Z(S) &= 2\sum_{y\in\mathcal{Y}} \sqrt{P_{X,Y}(0,y)P_{X,Y}(1,y)} && (10)\\
&= \sqrt{4P_X(0)P_X(1)} \sum_{y\in\mathcal{Y}} \sqrt{P_{Y|X}(y\,|\,0)P_{Y|X}(y\,|\,1)}
\end{aligned}
$$

which is the inner product between the unit vectors whose components are the square root of the distributions $P_{Y|X=0}$ and $P_{Y|X=1}$, under equiprobability $P_X(0) = P_X(1) = \frac{1}{2}$. This

quantity informs about the similarity between the side-information $Y$ when $X$ is 0 and 1, under equiprobability $P_X(0) = P_X(1) = \frac{1}{2}$.

Let

$$h(x) = -x \log x - (1-x)\log(1-x) \tag{11}$$

be the entropy function expressed in bits, which admits an inverse $h^{-1} : [0\,,\,1] \mapsto [0\,,\,\frac{1}{2}]$ when we restrict $x$ to be in $[0\,,\,\frac{1}{2}]$.

**Proposition 1.1 (Properties of Bhattacharyya parameter)** *Let $(X,Y) \simeq P_{X,Y}$ be an arbitrary pair of random variables over $\mathcal{B} \times \mathcal{Y}$ with $\mathcal{B} = \{0, 1\}$ and $\mathcal{Y}$ an arbitrary countable set. For any memoryless source $S = (X,Y) \simeq P_{X,Y}$, with $X$ as the part to be compressed and $Y$ in the role of "side-information" about $X$, we have*

$$Z(S)^2 \leq\ H(S)\ \leq \log\big(1 + Z(S)\big) \tag{12}$$

$$Z(S^+) = Z(S)^2 \quad \textit{and} \quad \sqrt{2Z(S)^2 - Z(S)^4} \leq Z(S^-) \leq 2Z(S) \tag{13}$$

The proof of the left inequality in (13) can be found in the paper[1] by Chou *et al.* and the proofs of Proposition 1.1 and Theorem 1.2 can be find in the paper by Şaşoğlu[2].

**Theorem 1.2 (Şaşoğlu)** *Let $(X_1, Y_1)$ and $(X_2, Y_2)$ be independent pairs of discrete random variables taking their values in $\mathcal{B} \times \mathcal{Y}_1$ and respectively in $\mathcal{B} \times \mathcal{Y}_2$ with $\mathcal{B} = \{0, 1\}$, and let $\mathbb{H}(X_1 \,|\, Y_1) = \alpha$ and $\mathbb{H}(X_2 \,|\, Y_2) = \beta$. Then, the conditional entropy $\mathbb{H}(X_1 \oplus X_2 \,|\, Y_1, Y_2)$ is minimized when $\mathbb{H}(X_1 \,|\, Y_1 = y_1) = \alpha$ and $\mathbb{H}(X_2 \,|\, Y_2 = y_2) = \beta$ for all $(y_1, y_2) \in \mathcal{Y}_1 \times \mathcal{Y}_2$ such that $P_{Y_1}(y_1)P_{Y_2}(y_2) > 0$. Moreover, if $\beta = \alpha = h(x)$ with $x \in [0\,,\,\frac{1}{2}]$ and if $0 < \alpha < 1$, then*

$$\min\big(\mathbb{H}(X_1 \oplus X_2 \,|\, Y_1, Y_2) - \mathbb{H}(X_1 \,|\, Y_1)\big) = h\big(2x(1-x)\big) - h(x) > 0, \tag{14}$$

*where the minimum in (14) is taken on the set $\{P_{X_1Y_1}, P_{X_2Y_2} : \mathbb{H}(X_1 \,|\, Y_1) = \mathbb{H}(X_2 \,|\, Y_2) = \alpha\}$.*

Finally, let us introduce

$$Z'(S) = 1 - Z(S)^2. \tag{15}$$

The next proposition results straightforwardly from Proposition 1.1 and definition (15).

**Proposition 1.3** *For any memoryless source $S$ with binary part to be compressed and discret "side-information", we have*

$$Z'(S^+) = 2Z'(S) - Z'(S)^2 \textit{ and } Z'(S^-) \leq Z'(S)^2. \tag{16}$$

# 2 Rough polarization

The following corollaries and theorems are adaptations to source polarization of results given by Guruswami and Xia[3] for channel polarization.

**Corollary 2.1 (Guruswami & Xia)** *There exists a constant $\theta_0$ with $0.799 < \theta_0 < 0.8$ such that for any memoryless source $S$ with binary-part to compress and discrete side-information,*

$$H(S^-) - H(S) = H(S) - H(S^+) \geq \theta_0 H(S)(1 - H(S)). \tag{17}$$

[1] The left inequality in (13) corresponds to Lemma 16 in "Polar coding for secret-key generation", Rémi Chou, Matthieu Bloch and Emmanuel Abbe, *IEEE Trans. on Information Theory*, vol. 61, no. 11, pp. 6213–6237, 2015.

[2] Proposition 1.1 corresponds to Proposition 2.8 and Lemma 2.9 and Theorem 1.2 corresponds to Lemma 2.2 in "Polarization and polar codes", Eren Şaşoğlu, *Fundations and Trends in Communications and Information Theory*, vol. 8, no. 4, pp. 259–381, 2011.

[3] Corollary 2.1, Theorem 2.3, Corollary 2.4 and Theorem 2.8 correspond respectively to Lemma 6, Lemma 8, Corollary 9 and Proposition 5 in "Polar codes: speed of polarization and polynomial gap to capacity", by Venkatesan Guruswami and Patrick Xia, *IEEE Transactions on Information Theory*, vol. 61, no. 1, pp. 3–16, 2015.

**Proof**. We write $S = \{(X_i, Y_i) : i \in \mathbb{N}^*\}$, a sequence of independent drawings from $(X, Y) \simeq P_{X,Y}$, as in Section 1. According to the definitions of $H(S^-)$ and $H(S)$, we have

$$H(S^-) - H(S) = H(X_1 \oplus X_2 \,|\, Y_1, Y_2) - H(X_1 \,|\, Y_1). \tag{18}$$

Using the entropy function $h$ defined in equation (11) and setting $H(X_1 \,|\, Y_1) = H(X_2 \,|\, Y_2) = h(x)$, with $x \in [0\,,\,\frac{1}{2}]$, it results from Theorem 1.2 that

$$H(S^-) - H(S) \geq h\big(2x(1-x)\big) - h(x). \tag{19}$$

Therefore

$$\frac{H(S^-) - H(S)}{H(S)\big(1 - H(S)\big)} \geq \frac{h\big(2x(1-x)\big) - h(x)}{h(x)\big(1 - h(x)\big)} \geq \min_{x \in [0\,,\,\frac{1}{2}]} \frac{h\big(2x(1-x)\big) - h(x)}{h(x)\big(1 - h(x)\big)} = \theta_0 \tag{20}$$

and numerical simulations give $0.799 < \theta_0 < 0.8$. In order to end the proof, let us remark that the transformation $(X_1, X_2) \mapsto (X_1 \oplus X_2, X_2)$ is invertible, hence $H(S^+) + H(S^-) = 2H(S)$, i.e., $H(S) - H(S^+) = H(S^-) - H(S)$. ◇

**Remark 2.1** *It results from Theorem 1.2 and its proof that for any $x \in \big[0\,,\,\frac{1}{2}\big]$, the inequality (19) can be an equality, hence the constant $\theta_0$ is the greatest value $\theta \in \mathbb{R}$ such that $H(S^-) - H(S) \geq \theta H(S)\big(1 - H(S)\big)$ for any memoryless source $S$ with binary-part to compress and discrete side-information.*

**Lemma 2.2** *The function*

$$\begin{array}{rcl} g \,:\, [0\,,\,1] & \to & \big[0\,,\,\frac{1}{2}\big] \\ \eta & \mapsto & \sqrt{\eta(1-\eta)} \end{array} \tag{21}$$

*is strictly concave and for any $\eta \in ]0\,,1\,[$, the function*

$$\begin{array}{rcl} G_\eta \,:\, [0\,,\,\min(\eta,\,1-\eta)] & \to & [0\,,\,1] \\ \delta & \mapsto & \frac{g(\eta+\delta)+g(\eta-\delta)}{2g(\eta)} \end{array} \tag{22}$$

*is strictly decreasing.*

**Proof**. The functions $g$ and $G_\eta$ are well defined for any $\eta \in ]0\,,\,1[$. Moreover, the two first derivatives of $g$ are

$$g'(\eta) = \frac{1 - 2\eta}{2g(\eta)} \qquad \text{and} \qquad g''(\eta) = \frac{-1}{g(\eta)}\left[1 + \frac{(1-2\eta)^2}{4\eta(1-\eta)}\right] < 0 \tag{23}$$

hence $g$ is strictly concave and finally for any $\eta \in ]0\,,\,1[$ and $\delta \in [0\,,\,\min(\eta,\,1-\eta)]$

$$G'_\eta(\delta) = \frac{g'(\eta+\delta) - g'(\eta-\delta)}{2g(\eta)} \leq 0, \tag{24}$$

with equality if and only if $\delta = 0$, which completes the proof. ◇

**Theorem 2.3 (Guruswami & Xia)** *Let $g$ be the function defined in (21). There exists a constant $\Lambda < 1$, with $0.9165 < \Lambda < 0.9166$, such that for any memoryless source $S$ with binary-part to compress and discrete side-information*

$$\frac{1}{2}\left[g\big(H(S^-)\big) + g\big(H(S^+)\big)\right] \leq \Lambda g\big(H(S)\big). \tag{25}$$

**Proof**. For a memoryless source $S$ with binary-part to compress and discrete side-information, let $\eta = H(S)$, $\varepsilon_0(\eta) = \theta_0\eta(1-\eta)$, where $\theta_0$ has been introduced in Corollary 2.1 and $\varepsilon = H(S^-) - H(S) = H(S) - H(S^+)$. It results from Corollary 2.1 that $\varepsilon \geq \varepsilon_0(\eta)$, which implies, according to Lemma 2.2, that

$$\begin{aligned}\frac{g\big(H(S^-)\big)+g\big(H(S^+)\big)}{2g\big(H(S)\big)} &= \frac{g(\eta+\varepsilon)+g(\eta-\varepsilon)}{2g(\eta)}\\ &\leq \frac{g(\eta+\varepsilon_0(\eta))+g(\eta-\varepsilon_0(\eta))}{2g(\eta)}\\ &= \frac{1}{2}\left(\sqrt{A_{\theta_0}(\eta)}+\sqrt{A_{\theta_0}(1-\eta)}\right) \qquad (26)\end{aligned}$$

with $A_{\theta_0}(\eta) = [1+\theta_0(1-\eta)](1-\theta_0\eta) = \theta_0^2(\eta-\theta_0^{-1})\left[\eta-(1+\theta_0^{-1})\right]$. The two roots of polynomial $A_{\theta_0}(\eta)$ are both outside the interval $[0\,,\,1]$, therefore the function $\eta \mapsto \sqrt{A_{\theta_0}(\eta)}$ is strictly convexe for $\eta \in [0\,,\,1]$. As a result, its derivative is injective and the term (26) is maximum for $\eta \in [0\,,\,1]$ if and only if

$$\frac{A'_{\theta_0}(\eta)}{\sqrt{A_{\theta_0}(\eta)}} = \frac{A'_{\theta_0}(1-\eta)}{\sqrt{A_{\theta_0}(1-\eta)}}, \qquad (27)$$

i.e., if and only if $\eta = \frac{1}{2}$. Hence, it comes

$$\begin{aligned}\frac{g\big(H(S^-)\big)+g\big(H(S^+)\big)}{2g\big(H(S)\big)} &\leq \frac{1}{2}\sqrt{[1+\theta_0(1-\eta)](1-\theta_0\eta)}+\frac{1}{2}\sqrt{[1-\theta_0(1-\eta)](1+\theta_0\eta)}\bigg|_{\eta=\frac{1}{2}}\\ &= \sqrt{1-\frac{\theta_0^2}{4}} = \Lambda. \qquad (28)\end{aligned}$$

Numerical simulations give $0.9165 < \Lambda < 0.9166$. ◇

A recursive application of Theorem 2.3 gives

$$\forall\mu\in\mathbb{N},\qquad \frac{1}{2^\mu}\sum_{i=0}^{2^\mu-1} g\left[H\left(S_\mu^{(i)}\right)\right] \leq \Lambda^\mu g\big[H(S)\big] \leq \Lambda^\mu \max_{\eta\in[0\,,\,1]} g(\eta) = \frac{1}{2}\Lambda^\mu. \qquad (29)$$

This last equation can be interpreted as

$$\mathrm{E}\left\{g\left[H\left(S_\mu^{(J)}\right)\right]\right\} \leq \frac{1}{2}\Lambda^\mu, \qquad (30)$$

where $J$ is a uniform random variable over $[\![0\,,\,2^\mu-1]\!]$. Hence, the next corollary results from Markov's inequality.

**Corollary 2.4 (Guruswami & Xia)** *For any memoryless source $S$, with binary-part to compress and discrete side-information, and the associated sequence introduced in equation (4), for any $\mu \in \mathbb{N}$, if $J$ is a uniform random variable over $[\![0\,,\,2^\mu-1]\!]$, then*

$$\forall\theta>0,\qquad \mathbb{P}\big(g^2\left[H\left(S_\mu^{(J)}\right)\right]\geq\theta\big) \leq \frac{\Lambda^\mu}{2\sqrt{\theta}}. \qquad (31)$$

We conclude this section by proving an adaptation to source polarization of the Guruswami and Xia rough (channel) polarization theorem. For any $\theta \in ]0\,,\,\frac{1}{2}]$, let

$$x_1 = x_1(\theta) = \frac{1-\sqrt{1-2\theta}}{2} \quad\text{and}\quad x_2 = x_2(\theta) = \frac{1+\sqrt{1-2\theta}}{2} = 1-x_1 \qquad (32)$$

be the solutions of $x(1-x) = \frac{\theta}{2}$. We have $0 \leq x_1 \leq \frac{1}{2} \leq x_2 \leq 1$ and

$$\left\{x\in[0\,,\,1]\,:\,x(1-x)\geq\frac{\theta}{2}\right\} = [x_1(\theta)\,,\,x_2(\theta)]\,. \qquad (33)$$

Moreover, $0 \leq 1-2\theta \leq \sqrt{1-2\theta} \leq 1$ implies $x_1(\theta) \leq \theta$. Hence, for any $m \in \mathbb{D}$ ($m = 2^\mu$), for any $\theta \in ]0\,,\,\frac{1}{2}[$ and for any memoryless source $S$ with binary-part to compress and discret side-information, we have

$$\begin{aligned}\left\{i \in [\![0\,,\,m-1]\!] \,:\, g^2\left[H\left(S_\mu^{(i)}\right)\right] < \frac{\theta}{2}\right\} &= \left\{i \in [\![0\,,\,m-1]\!] \,:\, H\left(S_\mu^{(i)}\right) \in [0\,,\,x_1[\cup]x_2\,,\,1]\right\} \\ &\subset \mathcal{V}_{X|Y}(x_1) \cup \mathcal{H}^c_{X|Y}(x_1) \end{aligned} \qquad (34)$$

and the following partition of $[\![0\,,\,m-1]\!]$:

$$[\![0\,,\,m-1]\!] = \mathcal{V}_{X|Y}(x_1) \cup \mathcal{H}^c_{X|Y}(x_1) \cup \left[\mathcal{V}^c_{X|Y}(x_1) \cap \mathcal{H}_{X|Y}(x_1)\right], \qquad (35)$$

implies, by setting $A = \mathcal{V}_{X|Y}(x_1)$, $B = \mathcal{H}^c_{X|Y}(x_1)$ and $C = \mathcal{V}^c_{X|Y}(x_1) \cap \mathcal{H}_{X|Y}(x_1)$,

$$\begin{aligned}1 - H(S) &= 1 - \frac{1}{m}\sum_{i=0}^{m-1} H\left(S_\mu^{(i)}\right) \\ &= \frac{1}{m}\left[\sum_{i\in A}\left(1 - H\left(S_\mu^{(i)}\right)\right) + \sum_{i\in B}\left(1 - H\left(S_\mu^{(i)}\right)\right) + \sum_{i\in C}\left(1 - H\left(S_\mu^{(i)}\right)\right)\right] & (36)\\ &\leq \frac{|A|}{m}\Big(1 - \min\left\{H\left(S_\mu^{(i)}\right) \,:\, i \in \mathcal{V}_{X|Y}(x_1)\right\}\Big) + \frac{|B|+|C|}{m} & (37)\\ &\leq x_1(\theta) + \frac{|B|+|C|}{m} \leq \theta + \frac{|B|}{m} + \mathbb{P}(J \in C), & (38)\end{aligned}$$

where $J$ is a random variable uniformly distributed over $[\![0\,,\,m-1]\!]$. Now, the contraposition of (34) gives

$$C \subset \left\{i \in [\![0\,,\,m-1]\!] \,:\, g^2\left[H\left(S_\mu^{(i)}\right)\right] \geq \frac{\theta}{2}\right\}, \qquad (39)$$

hence it results from Corollary 2.4 that

$$\mathbb{P}(J \in C) \leq \frac{\Lambda^\mu}{2\sqrt{\theta/2}}, \qquad (40)$$

and this implies, with inequality (38) where $\frac{|B|}{m} = \mathbb{P}\big(J \in \mathcal{H}^c_{X|Y}(x_1)\big)$, that

$$\mathbb{P}\big(J \in \mathcal{H}^c_{X|Y}(x_1)\big) \geq 1 - H(S) - \theta - \frac{\Lambda^\mu}{2\sqrt{\theta/2}}. \qquad (41)$$

**Proposition 2.5** *There exists $\Lambda \in ]0\,,\,1[$ such that for any $\theta \in ]0\,,\,\frac{1}{2}]$, for any memoryless source $S$ with binary-part to compress and discrete side-information, for any $m \in \mathbb{D}$ ($m = 2^\mu$), the subsets defined in equations (8–9) satisfy*

$$\begin{aligned}\frac{|\mathcal{H}_{X|Y}(\theta) \cap \mathcal{V}^c_{X|Y}(\theta)|}{m} &\leq \frac{\sqrt{2}\Lambda^\mu}{2\sqrt{\theta}} & (42)\\ H(S) - \theta \leq \frac{|\mathcal{H}_{X|Y}(\theta)|}{m} &\leq H(S) + \theta + \frac{\sqrt{2}\Lambda^\mu}{2\sqrt{\theta}} & (43)\\ H(S) - \theta - \frac{\sqrt{2}\Lambda^\mu}{2\sqrt{\theta}} \leq \frac{|\mathcal{V}_{X|Y}(\theta)|}{m} &\leq H(S) + \theta. & (44)\end{aligned}$$

**Proof**. Since $x_1 = x_1(\theta) \leq \theta$, we have $\mathcal{H}^c_{X|Y}(x_1) \subset \mathcal{H}^c_{X|Y}(\theta)$ and $\mathcal{V}_{X|Y}(x_1) \subset \mathcal{V}_{X|Y}(\theta)$. Therefore: firstly $\mathcal{H}_{X|Y}(\theta) \cap \mathcal{V}^c_{X|Y}(\theta) \subset \mathcal{H}_{X|Y}(x_1) \cap \mathcal{V}^c_{X|Y}(x_1)$ and inequality (42) results from (40);

secondly $|\mathcal{H}^c_{X|Y}(x_1)| \leq |\mathcal{H}^c_{X|Y}(\theta)|$ and the right inequality in (43) comes directly from (41). Furthermore, the conditions $\max_{i\in\mathcal{H}^c_{X|Y}(\theta)} H\big(S^{(i)}_\mu\big) \leq \theta$ and $\max_{i\in\mathcal{H}_{X|Y}(\theta)} H\big(S^{(i)}_\mu\big) \leq 1$ give

$$H(S) = \frac{1}{m}\sum_{i=0}^{m-1} H\big(S^{(i)}_\mu\big) \leq \theta\,\mathbb{P}\left[J \in \mathcal{H}^c_{X|Y}(\theta)\right] + \mathbb{P}\left[J \in \mathcal{H}_{X|Y}(\theta)\right] \leq \theta + \frac{|\mathcal{H}_{X|Y}(\theta)|}{m}, \tag{45}$$

which proves the left inequality in (43). Similarly, condition $\min_{i\in\mathcal{V}_{X|Y}(\theta)} H\big(S^{(i)}_\mu\big) \geq 1-\theta$ implies

$$H(S) \geq (1-\theta)\,\mathbb{P}\big[J \in \mathcal{V}_{X|Y}(\theta)\big] \geq \frac{|\mathcal{V}_{X|Y}(\theta)|}{m} - \theta, \tag{46}$$

which proves the right inequality in (44). Finally, note that $\mathcal{V}_{X|Y}(\theta) \subset \mathcal{H}_{X|Y}(\theta)$ implies $\mathcal{V}_{X|Y}(\theta) = \mathcal{H}_{X|Y}(\theta) \setminus \Big[\mathcal{H}_{X|Y}(\theta) \cap \mathcal{V}^c_{X|Y}(\theta)\Big]$, so the left inequality in (44) results from (42–43).$\diamond$

Let us now consider $\rho \in ]0\,,\,1[$ and $\mu \in \mathbb{N}^*$ such that $4\rho^{2\mu} < \frac{1}{2}$, i.e., $\mu > \frac{3}{2\log(1/\rho)}$. For any memoryless source $S$ with binary-part to compress and discrete side-information, since according to Proposition 1.1 for all $i \in [\![0\,,\,2^\mu-1]\!]$, $Z\big(S^{(i)}_\mu\big) \leq \sqrt{H\big(S^{(i)}_\mu\big)}$, we have

$$\big\{i \in [\![0\,,\,2^\mu-1]\!] \,:\, H\big(S^{(i)}_\mu\big) \leq 4\rho^{2\mu}\big\} \;\subset\; \big\{i \in [\![0\,,\,2^\mu-1]\!] \,:\, Z\big(S^{(i)}_\mu\big) \leq 2\rho^{\mu}\big\}, \tag{47}$$

$$\big\{i \in [\![0\,,\,2^\mu-1]\!] \,:\, H\big(S^{(i)}_\mu\big) \leq x_1(4\rho^{2\mu})\big\} \;\subset\; \big\{i \in [\![0\,,\,2^\mu-1]\!] \,:\, H\big(S^{(i)}_\mu\big) \leq 4\rho^{2\mu}\big\}; \tag{48}$$

therefore with $\theta = 4\rho^{2\mu}$ in (41), we obtain the following Proposition.

**Proposition 2.6** *With the notations introduced in this subsection, for any $\rho \in ]0\,,\,1[$, for any integer $\mu > \frac{3}{2\log(1/\rho)}$ we have*

$$\mathbb{P}\left(Z\big(S^{(J)}_\mu\big) \leq 2\rho^\mu\right) \geq 1 - H(S) - 4\rho^{2\mu} - \frac{1}{2\sqrt{2}}\left(\frac{\Lambda}{\rho}\right)^\mu, \tag{49}$$

*where $J$ is a random variable uniformly distributed over $[\![0\,,\,2^\mu-1]\!]$.*

Let us remark that

$$\forall\theta \in \left[0\,,\,\frac{\sqrt{5}-1}{2}\right[, \quad \sqrt{1-\theta} \leq 1-\theta^2 \tag{50}$$

and for any $\rho \in ]0\,,\,1[$, for any $\mu \in \mathbb{N}$ such that

$$\mu > \frac{2-\log(\sqrt{5}-1)}{\log(1/\rho)} \simeq \frac{1.69}{\log(1/\rho)} \qquad \text{i.e.,} \quad 2\rho^\mu < \frac{\sqrt{5}-1}{2}, \tag{51}$$

we have

$$\log\left(\frac{1}{1-2\rho^{2\mu}}\right) < \frac{1}{2} \qquad \text{i.e.,} \quad \mu > \left[\frac{3}{4} - \frac{\log(\sqrt{2}-1)}{2}\right]\frac{1}{\log(1/\rho)} \simeq \frac{1.38}{\log(1/\rho)}, \tag{52}$$

and it results from the right inequality in (12) that

$$\Big(H(S) > \log(2-4\rho^{2\mu})\Big) \Rightarrow \Big(1 + Z(S) > 2 - 4\rho^{2\mu}\Big). \tag{53}$$

Now, $\log(2-4\rho^{2\mu}) = 1 - \log\big[(1-2\rho^{2\mu})^{-1}\big]$, moreover $1 + Z(S) > 2 - 4\rho^{2\mu}$ if and only if $Z(S) > 1 - 4\rho^{2\mu}$ and $1 - 4\rho^{2\mu} \geq \sqrt{1-2\rho^\mu}$ according to (50–51), hence

$$\left(H(S) > 1 - \log\left[\frac{1}{1-2\rho^{2\mu}}\right]\right) \Rightarrow \Big(Z(S) > \sqrt{1-2\rho^\mu}\Big) \qquad \text{i.e.,} \quad Z'(S) = 1 - Z(S)^2 < 2\rho^\mu. \tag{54}$$

Finally,

$$1 - \log\left[\frac{1}{1-2\rho^{2\mu}}\right] = 1 + \frac{\ln(1-2\rho^{2\mu})}{\ln 2} \leq 1 - \frac{2\rho^{2\mu}}{\ln 2}. \tag{55}$$

Therefore the next proposition results from the left inequality in (44).

**Proposition 2.7** *With the notations introduced in this subsection, for any $\rho \in ]0\,,\,1[$, for any integer $\mu > \frac{2-\log(\sqrt{5}-1)}{\log(1/\rho)}$ we have*

$$\mathbb{P}\left(Z'\big(S_\mu^{(J)}\big) < 2\rho^\mu\right) \geq H(S) - \frac{2}{\ln 2}\rho^{2\mu} - \frac{\sqrt{\ln 2}}{2}\left(\frac{\Lambda}{\rho}\right)^\mu, \tag{56}$$

*where $J$ is a random variable uniformly distributed over $[\![0\,,\,2^\mu - 1]\!]$.*

%%%%%%%%%%%
We add this paragraph to prove the Rough polarization theorem.

Let $\rho \in ]\Lambda\,,\,1[$ and $\varepsilon \in ]0\,,\,\frac{1}{2}[$; let

$$b_\rho = \max\left(\frac{2}{\ln(1/\rho)}, \frac{1}{\ln(\rho/\Lambda)}\right), \tag{57}$$

and let $\mu \in \mathbb{N}$ such that $\mu > b_\rho \ln(1/\varepsilon)$. Then, we have $\frac{\ln(1/\varepsilon)-(\ln 2)/2}{\ln(\rho/\Lambda)} < \frac{\ln(1/\varepsilon)}{\ln(\rho/\Lambda)} \leq b_\rho \ln(1/\varepsilon) < \mu$ and $\frac{\ln(1/\varepsilon)+3\ln 2}{2\ln(1/\rho)} \leq \frac{4\ln(1/\varepsilon)}{2\ln(1/\rho)} \leq b_\rho \ln(1/\varepsilon) < \mu$, which imply

$$\frac{1}{2\sqrt{2}}\left(\frac{\Lambda}{\rho}\right)^\mu < \frac{\varepsilon}{2} \quad \text{and} \quad 4\rho^{2\mu} < \frac{\varepsilon}{2}, \quad \text{hence} \quad 4\rho^{2\mu} + \frac{1}{2\sqrt{2}}\left(\frac{\Lambda}{\rho}\right)^\mu < \varepsilon. \tag{58}$$

We proved the following theorem by Guruswami and Xia.

**Theorem 2.8 (Rough polarization)** *There exists $\Lambda \in ]0\,,\,1[$ such that for any $\rho \in ]\Lambda\,,\,1[$, there exists $b_\rho > 0$ such that for any memoryless source $S$ with binary-part to compress and discrete side-information, for any $\varepsilon \in ]0\,,\,\frac{1}{2}[$ and for any $\mu \in \mathbb{N}$, such that $\mu > b_\rho \ln(1/\varepsilon)$, there exists a* roughly polarized *set*

$$\mathcal{S}_r \subset \left\{S_\mu^{(i)}\,:\,0 \leq i < 2^\mu\right\} \tag{59}$$

*such that for any $M \in \mathcal{S}_r$, $Z(M) \leq 2\rho^\mu$ and $\mathbb{P}\big(S_\mu^{(J)} \in \mathcal{S}_r\big) \geq 1 - H(S) - \varepsilon$, where $J$ is a random variable uniformly distributed over $[\![0\,,\,2^\mu - 1]\!]$.*

%%%%%%%%%%%

# 3 Fine polarization

This section is an adaptation of the reasoning given by Guruswami and Xia (see footnote 3) to prove their fine polarization theorem.

## 3.1 Preliminaries

**Lemma 3.1** *For any $\beta \in ]0\,,\,\frac{1}{2}[$, the function $\zeta\,:\,\mathbb{R}_+^* \to \mathbb{R}_+$ defined by*

$$\zeta(y) = \frac{\lfloor y \rfloor}{2y} + \frac{2\beta^2 y}{\lceil y \rceil} = \begin{cases} \frac{1+4\beta^2}{2} & \text{if } y \in \mathbb{N}^* \\ \frac{q}{2(q+\alpha)} + \frac{2\beta^2(q+\alpha)}{q+1} & \text{if } y = q + \alpha \text{ with } q \in \mathbb{N} \text{ and } 0 < \alpha < 1, \end{cases} \tag{60}$$

*satisfies the condition:*

$$\forall y, \quad \left(\lfloor y \rfloor \geq \frac{4\beta^2}{1-4\beta^2} \Rightarrow \min\{\zeta(\lfloor y \rfloor + \alpha)\,:\,\alpha \in [0\,,\,1]\} = \frac{\lfloor y \rfloor}{2\big(\lfloor y \rfloor + 1\big)} + 2\beta^2\right). \tag{61}$$

**Proof**. For $q \in \mathbb{N}^*$, let us introduce the continuously differentiable function of $\alpha$

$$\begin{array}{rcl} f_q : \mathbb{R}_+ & \to & \mathbb{R}_+^* \\ \alpha & \mapsto & \frac{q}{2(q+\alpha)} + \frac{2\beta^2(q+\alpha)}{q+1}, \end{array} \quad (62)$$

which satisfies $f_q(\alpha) = \zeta(q+\alpha)$ ($\forall \alpha \in ]0\,,\,1[$) and

$$f_q(0) = \frac{1}{2} + 2\beta^2 \frac{q}{q+1} < \frac{1}{2} + 2\beta^2 \quad \text{and} \quad f_q(1) = \frac{q}{2(q+1)} + 2\beta^2 < \frac{1}{2} + 2\beta^2, \quad (63)$$

$$f_q'(\alpha) = \frac{2\beta^2}{q+1} - \frac{q}{2(q+\alpha)^2} \quad \text{and} \quad f_q'(\alpha) = 0 \Leftrightarrow \alpha = \frac{\sqrt{q(q+1)}}{2\beta} - q. \quad (64)$$

Let us remark that $\beta \in ]0\,,\,\frac{1}{2}[$ implies $f_q'(0) = \frac{-1}{2q} + \frac{2\beta^2}{q+1} < 0$ and the zero of the derivative function is always greater than zero. Moreover, the zero of the derivative function is smaller than 1 if and only if

$$\frac{q(q+1)}{4\beta^2} < (q+1)^2 \quad \Leftrightarrow \quad q < \frac{4\beta^2}{1-4\beta^2}. \quad (65)$$

Hence, if $\lfloor y \rfloor = q \geq \frac{4\beta^2}{1-4\beta^2}$, then $\min\{f_q(\alpha) : \alpha \in [0\,,\,1]\} = f_q(1) = \frac{\lfloor y \rfloor}{2(\lfloor y \rfloor + 1)} + 2\beta^2$. ◇

The proofs of the following three lemmas are straightforward.

**Lemma 3.2** *The function*

$$\begin{array}{rcl} \zeta : \mathbb{R}_+ & \to & \mathbb{R}_- \\ y & \mapsto & -\alpha_0 2^y + y \end{array} \quad \text{with} \quad \alpha_0 = \frac{2}{e \ln 2} \simeq 1.06 \quad (66)$$

*is maximum at the point* $y_0 = \frac{-\ln(\alpha_0 \ln 2)}{\ln 2} = \frac{1}{\ln 2} - 1 \simeq 0{,}44$ *and* $\zeta(y_0) = -1$.

**Lemma 3.3** *For any* $\beta \in ]0\,,\,\frac{1}{2}[$*, the function*

$$\begin{array}{rcl} \varphi : \mathbb{R}_+^* & \to & \mathbb{R}_+^* \\ y & \mapsto & \frac{1-\beta+y^{-1}}{1-2^{-\beta y}} \end{array} \quad (67)$$

*is strictly decreasing,* $\varphi(1/\beta) = 2$ *and* $\varphi(y)$ *approaches to* $1-\beta < 1$ *when* $y$ *approaches to infinity. Hence, there exists* $c_\beta > 0$ *such that*

$$\forall y,\ (y \geq c_\beta \Rightarrow \varphi(y) < 1)\,. \quad (68)$$

**Lemma 3.4** $\forall \beta \in ]0\,,\,\frac{1}{2}[$*,* $\forall \gamma \in \mathbb{R}_+^*$*,* $\forall \xi > 1$ *and* $\forall \rho \in ]0\,,\,1[$ *the function*

$$\begin{array}{rcl} \psi : \mathbb{R}_+ & \to & \mathbb{R}_+^* \\ y & \mapsto & \sqrt{e}\left(1 + \frac{\gamma\xi}{\log(1/\rho)}\right) \exp\left[\frac{-(1-2\beta)^2 y}{2}\right] \end{array} \quad (69)$$

*is strictly decreasing on* $\mathbb{R}_+$*,* $\psi(0) > 1$ *and* $\psi(y)$ *approaches to* 0 *when* $y$ *approaches to infinity.*

## 3.2 Introduction of parameters, constants and notations

Let $\delta \in ]0\,,\,\frac{1}{2}[$ and $\beta \in ]\delta\,,\,\frac{1}{2}[$ such that $\gamma = \frac{\delta}{\beta-\delta}$ is a rational number. We put $\gamma = \frac{\gamma_n}{\gamma_d}$ with $\gamma_n$ and $\gamma_d$ co-prime integers ($\gamma$ can take any value in $\mathbb{Q}_+^*$).
Let $\rho \in ]\Lambda\,,\,1[$, $\xi > 1$ (the $x$ parameter used by Guruswami and Xia is connected to $\xi$ with the relation $x = \frac{\log(1/\rho)}{\xi \log(2/\rho)}$) and

$$c = \left\lceil \frac{\gamma\xi}{\log(1/\rho)} \right\rceil. \quad (70)$$

Using the constant $c_\beta$ introduced in lemma 3.3, let

$$c'_\beta = \left(\frac{\xi}{\log(1/\rho)} + \frac{1}{\gamma}\right) \max\left\{c_\beta, \frac{1}{(1-2\beta)}\right\}, \tag{71}$$

$$c_\delta = \max\left(\frac{(1+\alpha_0)\xi}{(\xi-1)\log(1/\rho)}, c'_\beta\right). \tag{72}$$

Let $\mu$ be a natural integer multiple of $\gamma_d$ such that

$$\mu > c_\delta. \tag{73}$$

Finally, let us introduce

$$\nu = \gamma\mu \quad \text{and} \quad \nu_0 = (\gamma+1)\mu = \nu + \mu, \tag{74}$$

which are natural integers because $\mu$ is a multiple of $\gamma_d$.

For any $j \in [\![1\,,\,c]\!]$, let us note $I_j = \left[\frac{(j-1)\nu}{c}\,,\,\frac{j\nu}{c}\right[ \cap \mathbb{N}$, $n_j = |I_j|$ and

$$G_j(\nu) = \left\{ i = \sum_{k=0}^{\nu-1} b_k 2^k \;:\; \sum_{k\in I_j} b_k \geq \frac{\beta\nu}{c} \right\}, \quad G'_j(\nu) = \left\{ i = \sum_{k=0}^{\nu-1} b_k 2^k \;:\; \sum_{k\in I_j} (1-b_k) \geq \frac{\beta\nu}{c} \right\} \tag{75}$$

where $b_0, \ldots, b_{\nu-1}$ are the binary digits of $i$. It comes

$$\left\lfloor \frac{\nu}{c} \right\rfloor \leq n_j \leq \left\lceil \frac{\nu}{c} \right\rceil \quad \text{and} \quad \sum_{j=1}^{c} n_j = \nu. \tag{76}$$

Finally, let us put

$$G(\nu) = \bigcap_{j=1}^{c} G_j(\nu) \quad \text{and} \quad G'(\nu) = \bigcap_{j=1}^{c} G'_j(\nu). \tag{77}$$

### 3.3 Proof of the fine polarization theorem

**Lemma 3.5 (Guruswami & Xia)** *With the notations introduced in the previous subsections, if $J_2$ is a random variable with uniform distribution over $[\![0\,,\,2^\nu - 1]\!]$, then*

$$\mathbb{P}\big(J_2 \in G(\nu)\big) \;\geq\; 1 - \psi\left(\frac{\nu}{c}\right), \tag{78}$$

$$\mathbb{P}\big(J_2 \in G'(\nu)\big) \;\geq\; 1 - \psi\left(\frac{\nu}{c}\right). \tag{79}$$

**Proof**. If we write $J_2 = \sum_{k=0}^{\nu-1} B_k 2^k$, then the $\nu$ bits $B_k$ are independent Bernoulli random variables with parameter $\frac{1}{2}$. If $J_2$ takes its values in $G_j(\nu)$, we have

$$\sum_{k\in I_j} B_k - \frac{n_j}{2} \geq -\left(\frac{n_j}{2} - \frac{\beta\nu}{c}\right). \tag{80}$$

Moreover

$$\frac{n_j}{2} - \frac{\beta\nu}{c} \geq \frac{1}{2}\left\lfloor \frac{\nu}{c} \right\rfloor - \frac{\beta\nu}{c} > \frac{1}{2}\left(\frac{\nu}{c} - 1\right) - \frac{\beta\nu}{c} \tag{81}$$

and since, according to equations (70–74), we have

$$\frac{\nu}{c} \geq \frac{\gamma\mu}{1 + \frac{\gamma\xi}{\log(1/\rho)}} = \frac{\mu}{\frac{1}{\gamma} + \frac{\xi}{\log(1/\rho)}} > \frac{1}{(1-2\beta)}, \tag{82}$$

it comes

$$\frac{1}{2}\left(\frac{\nu}{c}-1\right)-\frac{\beta\nu}{c}=\frac{(1-2\beta)\nu}{2c}-\frac{1}{2}>0. \tag{83}$$

It then results from Hoeffding's inequality that

$$\begin{aligned}\mathbb{P}\big(J_2\in G_j(\nu)\big) &= 1-\mathbb{P}\left(\sum_{k\in I_j}B_k-\frac{n_j}{2}<-\left(\frac{n_j}{2}-\frac{\beta\nu}{c}\right)\right)\\ &\geq 1-\exp\left(-\left(\frac{1}{2}-\frac{\beta\nu}{cn_j}\right)^2 2n_j\right). \end{aligned}\tag{84}$$

Now, $\left(\frac{1}{2}-\frac{\beta\nu}{cn_j}\right)^2 2n_j=\frac{n_j}{2}-\frac{2\beta\nu}{c}+\frac{2\beta^2\nu^2}{c^2n_j}=\frac{\nu}{c}\left(\frac{n_j}{2\frac{\nu}{c}}-2\beta+\frac{2\beta^2\frac{\nu}{c}}{n_j}\right)\geq\frac{\nu}{c}\left(\zeta\left(\frac{\nu}{c}\right)-2\beta\right)$, where the last inequality comes from (76) and the $\zeta$ function is defined in equation (60). Further it results from condition (82) that[4] $\left\lfloor\frac{\nu}{c}\right\rfloor\geq\frac{4\beta^2}{1-4\beta^2}$. Therefore $\zeta\left(\frac{\nu}{c}\right)\geq\frac{\lfloor\frac{\nu}{c}\rfloor}{2\left(\lfloor\frac{\nu}{c}\rfloor+1\right)}+2\beta^2=\frac{1}{2}+2\beta^2-\frac{1}{2\left(\lfloor\frac{\nu}{c}\rfloor+1\right)}$, according to Lemma 3.1, and

$$\left(\frac{1}{2}-\frac{\beta\nu}{cn_j}\right)^2 2n_j\geq\frac{\nu(1-2\beta)^2}{2c}-\frac{1}{2}\frac{\frac{\nu}{c}}{\lfloor\frac{\nu}{c}\rfloor+1}\geq\frac{\nu}{c}\frac{(1-2\beta)^2}{2}-\frac{1}{2}. \tag{85}$$

Hence

$$\mathbb{P}\big(J_2\in G_j(\nu)\big)\geq 1-\sqrt{e}\exp\left[\frac{-(1-2\beta)^2\nu}{2c}\right] \tag{86}$$

and we obtain

$$\mathbb{P}\big(J_2\notin G(\nu)\big)\leq\sum_{j=1}^{c}P\big(J_2\notin G_j(\nu)\big)\leq c\sqrt{e}\exp\left[\frac{-(1-2\beta)^2\nu}{2c}\right]\leq\psi\left(\frac{\nu}{c}\right) \tag{87}$$

(the last inequality resulting from equations (70) and (69)), that proves (78). Finally, the same proof, where $B_k$ is replaced with $(1-B_k)$ in relations (80) and (84) and $G_j(\nu)$ is replaced with $G'_j(\nu)$ leads to (79). ◇

Now for any memoryless source $S$ with binary-part to compress and discrete side-information, it results from Proposition 2.6 (since[5] $\mu>\frac{3}{2\log(1/\rho)}$) that there exists $\mathcal{S}_r\subset\{S_\mu^{(i)}\ :\ 0\leq i<2^\mu\}$ such that

$$\forall M\in\mathcal{S}_r,\ Z(M)\leq 2\rho^\mu\quad\text{ and }\quad\mathbb{P}\left(S_\mu^{(J_1)}\in\mathcal{S}_r\right)\geq 1-H(S)-4\rho^{2\mu}-\frac{1}{2\sqrt{2}}\left(\frac{\Lambda}{\rho}\right)^\mu, \tag{88}$$

where $J_1$ is a random variable uniformly distributed over $[\![0\,,\,2^\mu-1]\!]$. In a same way, it results from Proposition 2.7 (since[6] $\mu>\frac{3}{2\log(1/\rho)}$) that there exists $\mathcal{S}'_r\subset\{S_\mu^{(i)}\ :\ 0\leq i<2^\mu\}$ such that

$$\forall M\in\mathcal{S}'_r,\ Z'(M)\leq 2\rho^\mu\quad\text{ and }\quad\mathbb{P}\left(S_\mu^{(J_1)}\in\mathcal{S}'_r\right)\geq H(S)-\frac{2}{\ln 2}\rho^{2\mu}-\frac{\sqrt{\ln 2}}{2}\left(\frac{\Lambda}{\rho}\right)^\mu. \tag{89}$$

For any $M\in\mathcal{S}_r$, we define the sequence

$$\tilde{Z}_k^{(i)}=\begin{cases}\left(\tilde{Z}_{k-1}^{(\lfloor i/2\rfloor)}\right)^2 & \text{if } i \text{ is odd,}\\ 2\tilde{Z}_{k-1}^{(\lfloor i/2\rfloor)} & \text{if } i \text{ is even,}\end{cases}\qquad\text{for any } k\in\mathbb{N}^*,\ \text{with } \tilde{Z}_0^{(0)}=Z(M). \tag{90}$$

[4]Indeed, the condition (82) implies

$$\left\lfloor\frac{\nu}{c}\right\rfloor\geq\frac{\nu}{c}-1>\frac{1}{1-2\beta}-1=\frac{2\beta}{1-2\beta}>\left(\frac{2\beta}{1-2\beta}\right)\left(\frac{2\beta}{1+2\beta}\right)=\frac{4\beta^2}{1-4\beta^2}.$$

[5]Indeed, condition (72) implies $\mu>\frac{(1+\alpha_0)\xi}{(\xi-1)\log(1/\rho)}>\frac{1+\alpha_0}{\log(1/\rho)}\simeq\frac{2.06}{\log(1/\rho)}>\frac{3}{2\log(1/\rho)}$.

[6]Indeed, condition (72) implies $\mu>\frac{(1+\alpha_0)\xi}{(\xi-1)\log(1/\rho)}>\frac{1+\alpha_0}{\log(1/\rho)}\simeq\frac{2.06}{\log(1/\rho)}>\frac{2-\log(\sqrt{5}-1)}{\log(1/\rho)}\simeq\frac{1.69}{\log(1/\rho)}$.

Let us note $R(\mu) = \{i \in [\![0\,,\,2^\mu - 1]\!] \,:\, S_\mu^{(i)} \in \mathcal{S}_r\}$.

In the same way, for any $M' \in \mathcal{S}'_r$, we define the sequence

$$\tilde{Z}_k^{\prime(i)} = \begin{cases} \left(\tilde{Z}_{k-1}^{\prime(\lfloor i/2 \rfloor)}\right)^2 & \text{if } i \text{ is even,} \\ 2\tilde{Z}_{k-1}^{\prime(\lfloor i/2 \rfloor)} & \text{if } i \text{ is odd,} \end{cases} \quad \text{for any } k \in \mathbb{N}^*, \text{ with } \tilde{Z}_0^{\prime(0)} = Z'(M'). \tag{91}$$

Let us note $R'(\mu) = \{i \in [\![0\,,\,2^\mu - 1]\!] \,:\, S_\mu^{(i)} \in \mathcal{S}'_r\}$.

**Lemma 3.6 (Guruswami & Xia)** *With the notations introduced in the previous subsection, we have* $\log\left(\max\left\{\tilde{Z}_\nu^{(i)} \,:\, i \in G(\nu)\right\}\right) \le -2^{\beta\nu}$.

**Proof**. Let us note for $j \in [\![1\,,\,c]\!]$, $\nu_j = \sum_{k=1}^{j} n_k$ (thus $\nu_c = \nu$) and

$$z_j = \max\left\{\tilde{Z}_{\nu_j}^{(\lfloor i/2^{\nu-\nu_j}\rfloor)} \,:\, i \in G(\nu)\right\} \text{ for any } j \in [\![1\,,\,c]\!] \quad \text{and } z_0 = Z(M). \tag{92}$$

Let us remark first that if $z_j$ has been attained by $p$ squarings ($0 \le p \le n_j$) and $n_j - p$ doublings from $z_{j-1}$, the maximum value will be obtained by applying first the $n_j - p$ doublings followed by the $p$ squarings[7]. Moreover, if $z_j < 1$, the maximum value will be reached by minimizing the number of squarings[8].

According to relations (72), (74), (70) and (88), we have $\mu > \frac{(1+\alpha_0)\xi}{(\xi-1)\log(1/\rho)}$, $M \in \mathcal{S}_r$, $\frac{\nu}{c} \le \frac{\mu\log(1/\rho)}{\xi}$ and

$$\log Z(M) + \frac{\nu}{c} \le 1 - \mu\log(1/\rho)(1 - 1/\xi) \le 1 - (1 + \alpha_0) = -\alpha_0 \le -1. \tag{93}$$

Equation (93) shows that $\log Z(M) + n_1 \le \log Z(M) + \frac{\nu}{c} + 1 < 0$, hence if one doubles $n_1$ times $z_0$ one obtains a value that is smaller than 1. Thus $z_1 < 1$ and since for any $i \in G(\nu)$, the number of squarings is worth at least $\lceil\frac{\beta\nu}{c}\rceil$, we have

$$\log z_1 \le 2^{\lceil\frac{\beta\nu}{c}\rceil}\left(\log Z(M) + \frac{\nu}{c} + 1 - \left\lceil\frac{\beta\nu}{c}\right\rceil\right) \le 2^{\frac{\beta\nu}{c}}\left(\log Z(M) + \frac{(1-\beta)\nu}{c} + 1\right), \tag{94}$$

which can be written, using the $\varphi$ function introduced in Lemma 3.3,

$$\log z_1 + \frac{\nu}{c}\varphi\left(\frac{\nu}{c}\right) \le 2^{\frac{\beta\nu}{c}}\left(\log Z(M) + \frac{\nu}{c}\varphi\left(\frac{\nu}{c}\right)\right) \le 2^{\frac{\beta\nu}{c}}\left(\log Z(M) + \frac{\nu}{c}\right), \tag{95}$$

according to condition (68) and

$$\frac{\nu}{c} \ge \frac{\gamma\mu}{1 + \frac{\gamma\xi}{\log(1/\rho)}} = \frac{\mu}{\frac{1}{\gamma} + \frac{\xi}{\log(1/\rho)}} > c_\beta \tag{96}$$

– the last inequality resulting from condition (73) and definitions (71–72) of $c_\delta$.

Moreover, according to Lemma 3.3, the $\varphi$ function is greater than $1-\beta$ and it results from (93) and (95) that

$$\log z_1 + \frac{\nu}{c} < -\alpha_0 2^{\frac{\beta\nu}{c}} + \frac{\beta\nu}{c} = \zeta\left(\frac{\beta\nu}{c}\right) \le -1, \tag{97}$$

where the $\zeta$ function is defined in Lemma 3.2. So $\frac{\nu}{c} + 1 + \log z_1 < 0$, therefore $z_2 < 1$ and the same reasoning as above leads to

$$\log z_2 \le 2^{\frac{\beta\nu}{c}}\left(\log z_1 + n_2 - \left\lceil\frac{\beta\nu}{c}\right\rceil\right) < 2^{\frac{\beta\nu}{c}}\left(\log z_1 + \frac{(1-\beta)\nu}{c} + 1\right), \tag{98}$$

---

[7] Indeed, starting from $x$, if we apply $p$ squarings and $n_j - p$ doublings, the final result will be of the form $x^{2^p}2^\alpha$, and $\alpha$, the power of 2, will be maximum if the $n_j - p$ doublings precede the $p$ squarings.

[8] If $z_j > 1$, the maximum value can be reached by replacing some doublings by squarings: starting from $x > 0$, $p+1$ squarings will give a greater result than $p$ squarings if and only if

$$2^p(\log x + n_j - p) \le 2^{p+1}(\log x + n_j - p - 1) \quad \Leftrightarrow \quad \log x \ge 2 - (n_j - p)\,.$$

which can be written

$$\log z_2 + \frac{\nu}{c}\varphi\left(\frac{\nu}{c}\right) \le 2^{\frac{\beta\nu}{c}}\left(\log z_1 + \frac{\nu}{c}\varphi\left(\frac{\nu}{c}\right)\right), \tag{99}$$

which, with (95), leads to

$$\log z_2 + (1-\beta)\frac{\nu}{c} \le \log z_2 + \frac{\nu}{c}\varphi\left(\frac{\nu}{c}\right) \le 2^{\frac{2\beta\nu}{c}}\left(\log z_0 + \frac{\nu}{c}\varphi\left(\frac{\nu}{c}\right)\right) \tag{100}$$

and according to conditions (93) and (96) and the property (68), it follows that

$$\begin{aligned}\log z_2 + \frac{\nu}{c} &\le 2^{\frac{2\beta\nu}{c}}\left(\log z_0 + \frac{\nu}{c}\right) + \frac{\beta\nu}{c}\\ &< -\alpha_0 2^{\frac{2\beta\nu}{c}} + \frac{\beta\nu}{c} = \zeta\left(\frac{2\beta\nu}{c}\right) - \frac{\beta\nu}{c} \le \zeta\left(\frac{2\beta\nu}{c}\right) \le -1,\end{aligned} \tag{101}$$

where the last inequality results from Lemma 3.2. More generally, let us suppose that

$$\log z_{j-1} + \frac{\nu}{c} \le 2^{\frac{(j-1)\beta\nu}{c}}\left(\log z_0 + \frac{\nu}{c}\right) + \frac{\beta\nu}{c}, \tag{102}$$

so $1 + \frac{\nu}{c} + \log z_{j-1} < 1 + \zeta\left(\frac{(j-1)\beta\nu}{c}\right) \le 0$ according to (93) and Lemma 3.2, and the same reasoning as above gives

$$\log z_j + \frac{(1-\beta)\nu}{c} \le 2^{\frac{j\beta\nu}{c}}\left(\log z_0 + \frac{\nu}{c}\right), \tag{103}$$

in particular for $j = c$ :

$$\log z_c \le 2^{\beta\nu}\left(\log z_0 + \frac{\nu}{c}\right) \le -2^{\beta\nu}, \tag{104}$$

where the last inequality results from relation (93). ◇

The same reasoning replacing $\tilde{Z}_k^{(i)}$ with $\tilde{Z}_k'^{(i)}$ leads to the following lemma.

**Lemma 3.7** *With the notations introduced in the previous subsection, we have*

$$\log\left(\max\left\{\tilde{Z}_\nu'^{(i)} : i \in G'(\nu)\right\}\right) \le -2^{\beta\nu}.$$

After all, we can deduce from equations (103–104) that

$$\log z_c + \frac{(1-\beta)\nu}{c} \le -2^{\beta\nu}. \tag{105}$$

Further, since $\log(1/\ln 2) \simeq 0.529$ and $\beta > 0$, we have

$$\beta\big(2\log(1/\ln 2) - 1\big) > 0 > \log(1/\ln 2) - 1, \qquad \text{i.e.,} \quad \frac{1}{1-2\beta} > \frac{\log(1/\ln 2)}{1-\beta}, \tag{106}$$

which implies, according to the conditions (71,73) and the equation (70),

$$\mu > \left(\frac{\xi}{\log(1/\rho)} + \frac{1}{\gamma}\right)\frac{1}{1-2\beta} > \frac{c\log(1/\ln 2)}{\gamma(1-\beta)}, \tag{107}$$

i.e., $\frac{(1-\beta)\nu}{c} > \log(1/\ln 2)$. Thus the relation (105) leads to the following lemma.

**Lemma 3.8** *With the notations introduced in the previous subsection, we have*

$$\log\left(\max\left\{\tilde{Z}_\nu^{(i)} : i \in G(\nu)\right\}\right) \le -2^{\beta\nu} + \log(\ln 2). \tag{108}$$

Let us recall that $\nu_0 = \nu + \mu$ and let us summarize the results proved in this section. We expanded $i \in [\![0\,,\,2^{\nu_0}-1]\!]$ into $i = i_1 + 2^\mu i_2$, where the binary digits of $i_2 \in [\![0\,,\,2^{\nu}-1]\!]$ and $i_1 \in [\![0\,,\,2^{\mu}-1]\!]$ correspond respectively to the first $\nu$ and last $\mu$ bits of $i$. We proved that if $i_1 \in R(\mu)$ (i.e., if $S_\mu^{(i_1)} \in \mathcal{S}_r$ or equivalently if $Z(S_\mu^{(i_1)}) < 2\rho^\mu$) and if $i_2 \in G(\nu)$, then

$$Z(S_{\nu_0}^{(i)}) \leq 2^{-2^{\beta\nu}} \ln 2 = 2^{-2^{\delta\nu_0}} \ln 2. \tag{109}$$

We also proved that if $i_1 \in R'(\mu)$ (i.e., if $S_\mu^{'(i_1)} \in \mathcal{S}'_r$ or equivalently if $Z'(S_\mu^{(i_1)}) < 2\rho^\mu$) and if $i_2 \in G'(\nu)$, then

$$Z'(S_{\nu_0}^{(i)}) \leq 2^{-2^{\beta\nu}} = 2^{-2^{\delta\nu_0}}. \tag{110}$$

Now, according to equations (88) and (78) and assuming $J = J_1 + 2^\mu J_2$ is a random variable uniformly distributed over $[\![0\,,\,2^{\nu_0}-1]\!]$, we have

$$\begin{aligned}
\mathbb{P}\big(J_1 \in R(\mu) \text{ and } J_2 \in G(\nu)\big) &= \mathbb{P}\big(J_1 \in R(\mu)\big)\,\mathbb{P}\big(J_2 \in G(\nu)\big) \\
&\geq \left(1 - H(S) - 4\rho^{2\mu} - \frac{1}{2\sqrt{2}}\left(\frac{\Lambda}{\rho}\right)^\mu\right)\left(1 - \psi\left(\frac{\nu}{c}\right)\right) \\
&\geq 1 - H(S) - 4\rho^{2\mu} - \frac{1}{2\sqrt{2}}\left(\frac{\Lambda}{\rho}\right)^\mu - \psi\left(\frac{\nu}{c}\right).
\end{aligned} \tag{111}$$

We deduce that

$$\begin{aligned}
\mathbb{P}\left(Z(S_{\nu_0}^{(J)}) \leq 2^{-2^{\delta\nu_0}} \ln 2\right) &\geq \mathbb{P}\big(J_1 \in R(\mu) \text{ and } J_2 \in G(\nu)\big) \\
&\geq 1 - H(S) - 4\rho^{2\mu} - \frac{1}{2\sqrt{2}}\left(\frac{\Lambda}{\rho}\right)^\mu - \psi\left(\frac{\nu}{c}\right).
\end{aligned} \tag{112}$$

In a same way, we have

$$\begin{aligned}
\mathbb{P}\left(Z'(S_{\nu_0}^{(J)}) \leq 2^{-2^{\delta\nu_0}}\right) &\geq \mathbb{P}\big(J_1 \in R'(\mu) \text{ and } J_2 \in G'(\nu)\big) \\
&\geq H(S) - \frac{2}{\ln 2}\rho^{2\mu} - \frac{\sqrt{\ln 2}}{2}\left(\frac{\Lambda}{\rho}\right)^\mu - \psi\left(\frac{\nu}{c}\right).
\end{aligned} \tag{113}$$

Thus, we proved that for any $\mu$ multiple of $\gamma_d$: $\mu = k\gamma_d$ with $k \in \mathbb{N}^*$ great enough ($k > \frac{c_\delta}{\gamma_d}$) or in other words for any sufficiently large $\nu_0 = \mu(1+\gamma) = k(\gamma_d + \gamma_n)$, the relations (109–110) and (112–113) are valid.

Let us consider now $\nu'_0$ between two successive multiples of $\gamma_d + \gamma_n$:

$$\nu'_0 = k(\gamma_d + \gamma_n) + u = \mu(1+\gamma') \tag{114}$$

with

$$\mu = k\gamma_d, \quad 0 \leq u < \gamma_n + \gamma_d \quad \text{ and } \quad \gamma' = \frac{k\gamma_n + u}{k\gamma_d} = \gamma + \frac{u}{k\gamma_d}, \tag{115}$$

then

$$\gamma \leq \gamma' = \gamma + \frac{u}{k\gamma_d} \leq \gamma + \frac{u}{\gamma_d} < 1 + 2\gamma. \tag{116}$$

Let us remark that if we introduce $\delta'$ such that

$$\gamma' = \frac{\delta'}{\beta - \delta'} \geq \gamma = \frac{\delta}{\beta - \delta}, \tag{117}$$

then $\delta \leq \delta'$ and $2^{-2^{\delta'\nu'_0}} \leq 2^{-2^{\delta\nu'_0}}$. Thus, by replacing $\gamma$ with $1 + 2\gamma$ in the definition of $\psi$ (see (69)), leaving $\gamma$ unchanged in equation (71) and replacing $\gamma$ with $\gamma'$ everywhere else, the above reasoning can be remade in order to prove the following proposition.

**Proposition 3.9** *For any* $\delta \in \left]0\,,\,\frac{1}{2}\right[$*, for any* $\beta \in \left]\delta\,,\,\frac{1}{2}\right[$*, for any* $\rho \in ]0\,,\,1[$*, for any* $\xi > 1$*, there exists* $C_{\delta,\beta} > 0$ *and* $A_{\delta,\beta} > 0$ *such that for any memoryless source* $S$ *with binary-part to compress and discrete side-information and for any integer* $\nu_0 > C_{\delta,\beta}$*, we have*

$$\mathbb{P}\left(Z(S_{\nu_0}^{(J)}) \leq 2^{-2^{\delta\nu_0}} \ln 2\right) \geq 1 - H(S) - 4\rho^{2\mu} - \frac{1}{2\sqrt{2}}\left(\frac{\Lambda}{\rho}\right)^{\mu} - A_{\delta,\beta} \exp\left(\frac{-(1-2\beta)^2\nu}{2c}\right), \quad (118)$$

$$\mathbb{P}\left(Z'(S_{\nu_0}^{(J)}) \leq 2^{-2^{\delta\nu_0}}\right) \geq H(S) - \frac{2}{\ln 2}\rho^{2\mu} - \frac{\sqrt{2}}{2}\left(\frac{\Lambda}{\rho}\right)^{\mu} - A_{\delta,\beta} \exp\left(\frac{-(1-2\beta)^2\nu}{2c}\right), \quad (119)$$

*where* $J$ *is a random variable uniformly distributed over* $[\![0\,,\,2^{\nu_0}-1]\!]$, $\gamma = \frac{\delta}{\beta-\delta}$, $\nu_0 = (\gamma+1)\mu$, $\nu = \gamma\mu$ *and* $c = \left\lceil \frac{\gamma\xi}{\log(1/\rho)} \right\rceil$.

Let us denote

$$\varepsilon = \frac{\mu}{\nu_0} = \frac{1}{\gamma+1} = \frac{\beta-\delta}{\beta}, \quad \text{hence} \quad \beta = \frac{\delta}{1-\varepsilon} \quad \text{and} \quad \gamma = \frac{1}{\varepsilon} - 1. \quad (120)$$

Now we can choose $\xi > 1$ so that the fraction $\frac{\gamma\xi}{\log(1/\rho)}$ is an integer (equal to $c$), then we have

$$\frac{\nu}{c} = \frac{\gamma\mu}{c} = \frac{\mu\log(1/\rho)}{\xi} \quad (121)$$

and the previous proposition becomes:

**Proposition 3.10** *For any* $\delta \in \left]0\,,\,\frac{1}{2}\right[$*, for any* $\rho \in ]0\,,\,1[$*, for any* $\varepsilon \in ]0\,,\,1-2\delta[$*, for any* $\xi > 1$ *such that* $\frac{(1-\varepsilon)\xi}{\varepsilon\log(1/\rho)} \in \mathbb{N}^\star$*, there exists* $C_{\delta,\varepsilon} > 0$ *and* $A_{\delta,\varepsilon} > 0$ *such that for any memoryless source* $S$ *with binary-part to compress and discrete side-information and for any integer* $\nu_0 > C_{\delta,\varepsilon}$*, we have*

$$\begin{aligned}\mathbb{P}\left(Z(S_{\nu_0}^{(J)}) \leq 2^{-2^{\delta\nu_0}} \ln 2\right) &\geq 1 - H(S) - 4\rho^{2\varepsilon\nu_0} - \frac{1}{2\sqrt{2}}\left(\frac{\Lambda}{\rho}\right)^{\varepsilon\nu_0} \\ &\qquad - A_{\delta,\varepsilon}\exp\left(\frac{-(1-\frac{2\delta}{1-\varepsilon})^2\log(1/\rho)\varepsilon\nu_0}{2\xi}\right) \\ &= 1 - H(S) - 4\rho^{2\varepsilon\nu_0} - \frac{1}{2\sqrt{2}}\left(\frac{\Lambda}{\rho}\right)^{\varepsilon\nu_0} - A_{\delta,\varepsilon}\left[(\rho)^{\left(1-\frac{2\delta}{1-\varepsilon}\right)^2\frac{1}{2\xi\ln 2}}\right]^{\varepsilon\nu_0} \quad (122)\\ \mathbb{P}\left(Z'(S_{\nu_0}^{(J)}) \leq 2^{-2^{\delta\nu_0}}\right) &\geq H(S) - \frac{2}{\ln 2}\rho^{2\varepsilon\nu_0} - \frac{\sqrt{\ln 2}}{2}\left(\frac{\Lambda}{\rho}\right)^{\varepsilon\nu_0} - A_{\delta,\varepsilon}\left[(\rho)^{\left(1-\frac{2\delta}{1-\varepsilon}\right)^2\frac{1}{2\xi\ln 2}}\right]^{\varepsilon\nu_0}, \quad (123)\end{aligned}$$

*where* $J$ *is a random variable uniformly distributed over* $[\![0\,,\,2^{\nu_0}-1]\!]$*. Moreover we can choose*

$$A_{\delta,\varepsilon} = \sqrt{e}\left(1 + \frac{(2-\varepsilon)\xi}{\varepsilon\log(1/\rho)}\right). \quad (124)$$

Let us put $n = 2^{\nu_0}$. Equation (122) can be written

$$\mathbb{P}\left(Z(S_{\nu_0}^{(J)}) \leq 2^{-n^\delta}\ln 2\right) \geq 1 - H(S) - \frac{4}{n^{\kappa_\epsilon^{(1)}}} - \frac{1}{2\sqrt{2}\cdot n^{\kappa_\epsilon^{(2)}}} - \frac{A_{\delta,\varepsilon}}{n^{\kappa_\epsilon^{(3)}}} \quad (125)$$

with

$$\begin{aligned}\kappa_\epsilon^{(1)} &= 2\varepsilon\log(1/\rho), \quad (126)\\ \kappa_\epsilon^{(2)} &= \varepsilon\log(\rho/\Lambda) \quad (127)\\ \kappa_\epsilon^{(3)} &= \varepsilon\left(1-\frac{2\delta}{1-\varepsilon}\right)^2\frac{\log(1/\rho)}{2\xi\ln 2}. \quad (128)\end{aligned}$$

In order to shorten the notations, let us put

$$\alpha = \alpha(\delta, \varepsilon, \rho, \xi) = \left(1 - \frac{2\delta}{1-\varepsilon}\right)^2 \frac{1}{2\xi \ln 2}. \tag{129}$$

For fixed $\delta \in \left]0\,,\, \frac{1}{2}\right[$, we look for $\varepsilon \in ]0\,,\,1-2\delta[$, $\rho \in ]0\,,\,1[$ and $\xi > 1$ that maximize $\kappa_\epsilon = \min\left(\kappa_\epsilon^{(1)}, \kappa_\epsilon^{(2)}, \kappa_\epsilon^{(3)}\right)$. Let us remark that

$$\kappa_\epsilon^{(3)} = \varepsilon\left(1 - \frac{2\delta}{1-\varepsilon}\right)^2 \frac{\log(1/\rho)}{2\xi \ln 2} < \frac{\varepsilon \log(1/\rho)}{2\ln 2} < 2\varepsilon \log(1/\rho) = \kappa_\epsilon^{(1)}, \tag{130}$$

therefore $\min\left(\kappa_\epsilon^{(1)}, \kappa_\epsilon^{(2)}, \kappa_\epsilon^{(3)}\right) = \min\left(\kappa_\epsilon^{(2)}, \kappa_\epsilon^{(3)}\right)$. Moreover, we have

$$\kappa_\epsilon^{(2)} \leq \kappa_\epsilon^{(3)} \Leftrightarrow \log(\rho/\Lambda) \leq \alpha \log(1/\rho) \Leftrightarrow \log \rho \leq \frac{\log \Lambda}{1+\alpha} \Leftrightarrow \rho \leq \Lambda^{\frac{1}{1+\alpha}}. \tag{131}$$

Firstly, let us suppose that

$$\rho \leq \Lambda^{\frac{1}{1+\alpha}}. \tag{132}$$

In this case we have $\min\left(\kappa_\epsilon^{(1)}, \kappa_\epsilon^{(2)}, \kappa_\epsilon^{(3)}\right) = \kappa_\epsilon^{(2)} = \varepsilon \log(\rho/\Lambda)$ and this expression is maximum when the independent variables $\rho$ and $\varepsilon$ are maximum, hence for $\rho = \Lambda^{\frac{1}{1+\alpha}}$, which leads to

$$\min\left(\kappa_\epsilon^{(1)}, \kappa_\epsilon^{(2)}, \kappa_\epsilon^{(3)}\right) = \kappa_\epsilon^{(2)} = \kappa_\epsilon^{(3)} = \log(1/\Lambda)\frac{\varepsilon\alpha}{1+\alpha} \qquad \text{with} \qquad \alpha = \alpha(\varepsilon, \xi), \tag{133}$$

since $\delta$ is supposed to be fixed. Secondly, if we suppose that

$$\rho \geq \Lambda^{\frac{1}{1+\alpha}}, \tag{134}$$

then $\min\left(\kappa_\epsilon^{(1)}, \kappa_\epsilon^{(2)}, \kappa_\epsilon^{(3)}\right) = \kappa_\epsilon^{(3)} = \varepsilon\alpha \log(1/\rho)$ and this quantity is maximum when $\alpha$ is maximum and $\rho$ minimum, i.e., when inequality (134) is an equality, i.e., when (133) is satisfied.

Further, the exponent $\kappa_\epsilon^{(2)} = \kappa_\epsilon^{(3)}$ in equation (133) is maximum if and only if

$$g(\varepsilon, \xi) \stackrel{\text{def}}{=} \frac{\varepsilon\alpha(\varepsilon, \xi)}{1+\alpha(\varepsilon, \xi)} \tag{135}$$

is maximum. Since

$$\frac{\partial g}{\partial \xi}(\varepsilon, \xi) = \frac{\varepsilon}{(1+\alpha)^2} \cdot \frac{\partial \alpha}{\partial \xi}(\varepsilon, \xi) = \frac{-\varepsilon\left(1 - \frac{2\delta}{1-\varepsilon}\right)^2}{(1+\alpha)^2 2\xi^2 \ln 2} < 0, \tag{136}$$

$g(\varepsilon, \xi)$ is maximum when

$$\xi = \xi_{\text{min}} > 1 \tag{137}$$

and equation (129) implies that

$$\alpha(\varepsilon, \xi_{\text{min}}) = \frac{\left(1 - \frac{2\delta}{1-\varepsilon}\right)^2}{2\xi_{\text{min}} \ln 2} \stackrel{\text{def}}{=} \alpha_{\text{max}}(\varepsilon). \tag{138}$$

Finally, the function to maximize is

$$\tilde{g}(\varepsilon) \stackrel{\text{def}}{=} \frac{\varepsilon\alpha_{\text{max}}(\varepsilon)}{1+\alpha_{\text{max}}(\varepsilon)} \tag{139}$$

whose derivative

$$\tilde{g}'(\varepsilon) = \frac{\alpha_{\text{max}}(\varepsilon)}{1+\alpha_{\text{max}}(\varepsilon)} + \frac{\varepsilon\alpha'_{\text{max}}(\varepsilon)}{\left(1+\alpha_{\text{max}}(\varepsilon)\right)^2} \tag{140}$$

vanishes if and only if $\alpha_{\max}(\varepsilon)\big(1+\alpha_{\max}(\varepsilon)\big)+\varepsilon\alpha'_{\max}(\varepsilon)=0$. We obtain a trivial solution $\varepsilon=1-2\delta$ (corresponding to a minimum: $\tilde{g}(1-2\delta)=0$) and a third degree algebraic equation in $u$, with $u=1-\varepsilon$:

$$P(u)=Au^3-Bu^2-Cu-D, \quad \text{with} \quad \left\{\begin{array}{lcl} A &=& 2\xi_{\min}\ln 2+1 \\ B &=& 2\delta(3-2\xi_{\min}\ln 2) \\ C &=& 4\delta(2\xi_{\min}\ln 2-3\delta) \\ D &=& 8\delta^3 \end{array}\right. . \tag{141}$$

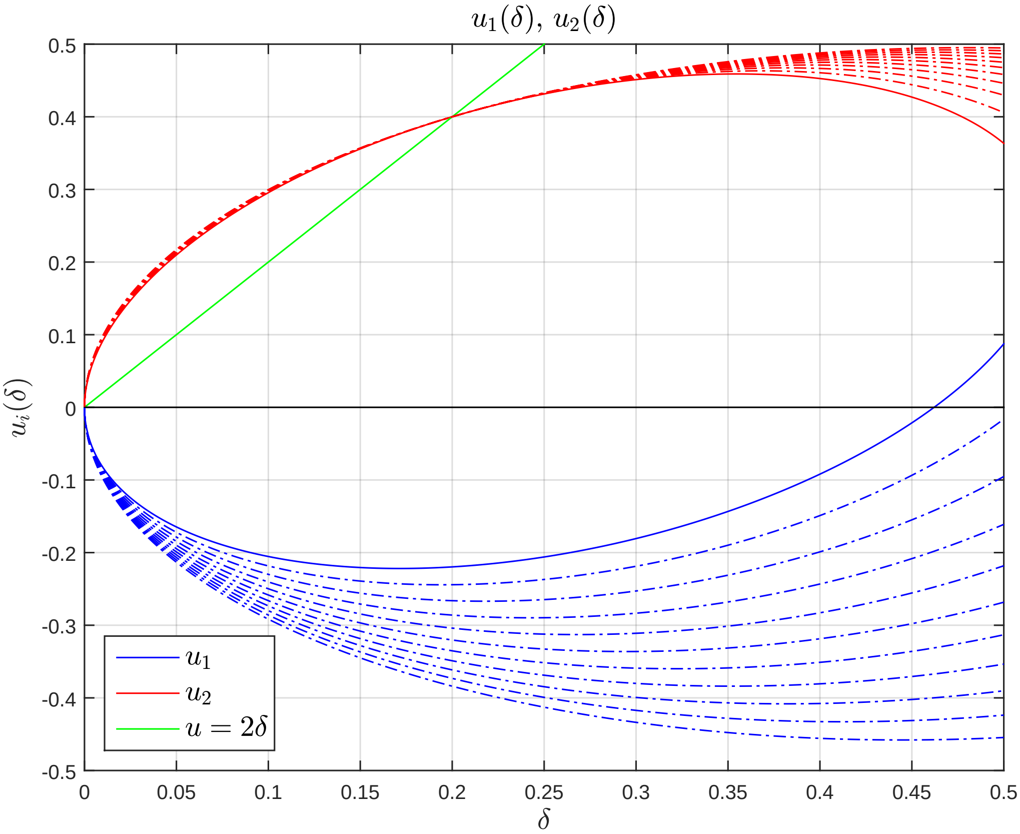


Figure 1: *Graphs of $u_1(\delta)$ and $u_2(\delta)$, the roots of $P'(u)$, for $\delta\in]0\,,\,\frac{1}{2}[$ and various values of $\xi_{\min}=(10+k)/10$ $(0\le k\le 10)$; solid line corresponds to $\xi_{\min}=1$.*

The discriminant of $P$ is the resultant $\mathrm{Res}(P,P')$ between polynomials $P(u)$ and its derivative $P'(u)=3Au^2-2Bu-C$:

$$\mathrm{Res}(P,P') = \begin{vmatrix} A & -B & -C & -D & 0 \\ 0 & A & -B & -C & -D \\ 0 & 0 & 3A & -2B & -C \\ 0 & 3A & -2B & -C & 0 \\ 3A & -2B & -C & 0 & 0 \end{vmatrix} \tag{142}$$

$$= 256(\xi_{\min}\ln 2)^2(2\xi_{\min}\ln 2+1)(4\delta^2-4\delta+2\xi_{\min}\ln 2+1)\delta^3 \times\big(8\xi_{\min}\ln 2-\delta(27-2\xi_{\min}\ln 2)\big). \tag{143}$$

The third degree equation $P(u)=0$ admits a multiple root if and only if the resultant $\mathrm{Res}(P,P')$ vanishes, i.e., if and only if $\delta=0$ or (see Figure 2)

$$\delta=\delta_1(\xi_{\min})=\frac{8\xi_{\min}\ln 2}{27-2\xi_{\min}\ln 2} \underset{\xi_{\min}=1}{\simeq} 0.21649. \tag{144}$$

Thus for all $\delta \in ]0\,,\, \delta_1(\xi_{\min})[$, the equation $P(u) = 0$ admits three real roots, for $\delta > \delta_1(\xi_{\min})$ the same equation admits only one real root and for $\delta = \delta_1(\xi_{\min})$, the real root is multiple. Moreover, let us introduce the discriminant of $P'(u)$:

$$\Delta' = B^2 + 3AC = 8\delta\xi_{\min} \ln 2\big(6\xi_{\min} \ln 2 + 3 - \delta(15 - 2\xi_{\min} \ln 2)\big), \tag{145}$$

which vanishes when

$$\delta = \delta_0(\xi_{\min}) = \frac{6\xi_{\min} \ln 2 + 3}{15 - 2\xi_{\min} \ln 2} \underset{\xi_{\min}=1}{\simeq} 0.52586, \tag{146}$$

and, for $\delta \in \left]0\,,\, \frac{1}{2}\right[$, let

$$u_1(\delta) = \frac{B - \sqrt{\Delta'}}{3A} < 0 \qquad \text{and} \qquad u_2(\delta) = \frac{B + \sqrt{\Delta'}}{3A} > 0 \tag{147}$$

be the real roots of $P'(u)$ (see Figure 1). We have $P\left(u_1\big(\delta_1(\xi_{\min})\big)\right) = 0$ and $u_2(\delta_2) = 2\delta_2$ with $\delta_2 = 1/5 = 0.2$.

We show on Figure 2 the graphs of the values of $P(u)$ when $P'(u)$ vanishes as functions of $\delta$ for different values of $\xi_{\min}$ and we can see that for all $\delta \in ]0\,,\, \delta_1(\xi_{\min})[$, $P(u_1) > 0$. Further,

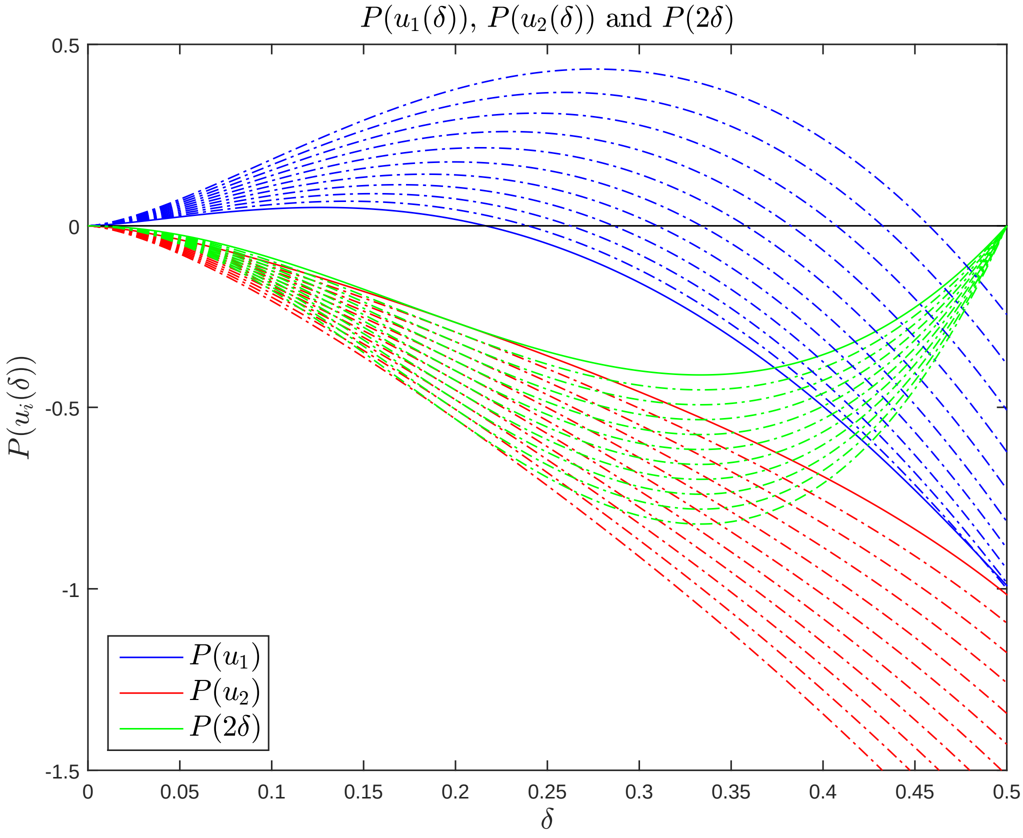


Figure 2: *Graphs of $P(u_1(\delta))$, $P(u_2(\delta))$ and $P(2\delta)$ for $\delta \in ]0\,,\, \frac{1}{2}[$ and various values of $\xi_{\min} = (10 + k)/10$ $(0 \le k \le 10)$; solid line corresponds to $\xi_{\min} = 1$.*

since $P(0) = -D < 0$ $(\forall\delta > 0)$ and $P(u) \to -\infty$ when $u \to -\infty$ $(\forall\delta)$, we deduce that when equation $P(u) = 0$ admits three real zeros (i.e., when $0 < \delta < \delta_1(\xi_{\min})$), two of the three roots are smaller than zero.

Finally, since polynomial $4\delta^2 - 4\delta + 2\xi_{\min} \ln 2 + 1$ has no real roots, we can remark that

$$P(1) = A - B - C - D = (1 - 2\delta)(4\delta^2 - 4\delta + 2\xi_{\min} \ln 2 + 1) > 0 \quad \text{for all} \quad \delta \in \left]0\,,\, \frac{1}{2}\right[ \tag{148}$$

$$P(2\delta) = 8A\delta^3 - 4B\delta^2 - 2C\delta - D = -16\xi_{\min}(\ln 2)\delta^2(1 - 2\delta) < 0 \quad \text{for all} \quad \delta \in \left]0\,,\, \frac{1}{2}\right[ \tag{149}$$

Therefore, for all $\delta \in ]0\,,\,\frac{1}{2}[$, there is always one and only one zero of $P(u)$ with $2\delta < u < 1$. Let us note $\nu_1(\delta)$ this root of $P$. All the above mentioned conditions on the real roots $\nu_i(\delta)$ $(1 \leq i \leq 3)$ of $P(u)$ can be observed on Figure 3, which has been obtained with numerical simulations.

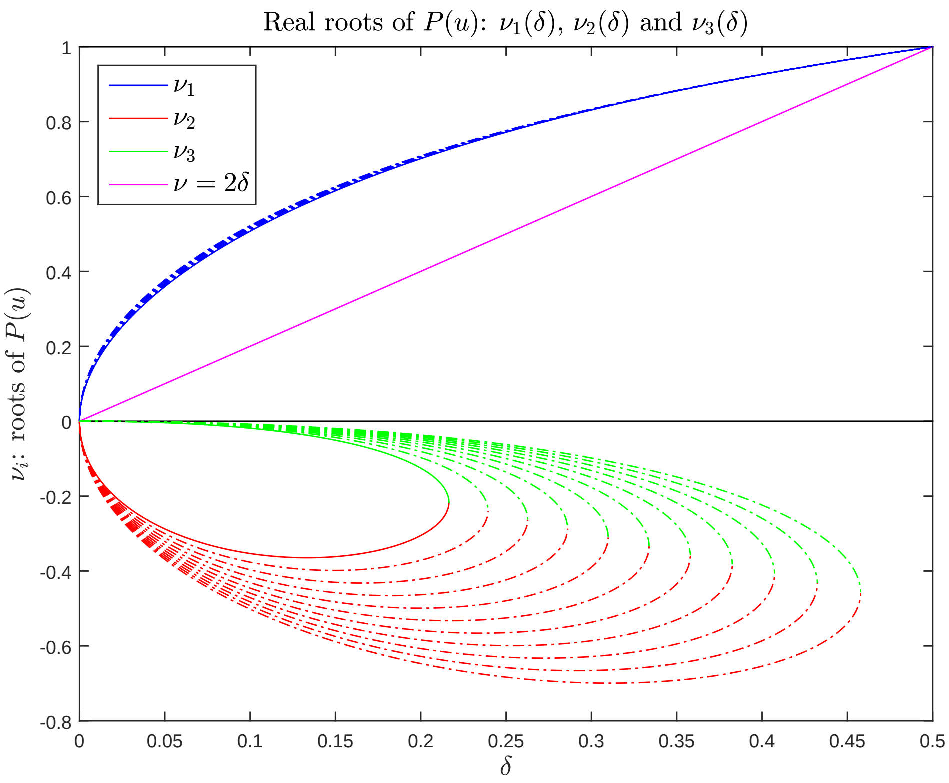


Figure 3: *Graphs of* $\nu_1(\delta)$, $\nu_2(\delta)$, $\nu_3(\delta)$ *the real roots of* $P(u)$ *and of* $\nu = 2\delta$ *for* $\delta \in ]0\,,\,\frac{1}{2}[$ *and various values of* $\xi_{\min} = (10+k)/10$ $(0 \leq k \leq 10)$*; solid line corresponds to* $\xi_{\min} = 1$.

We show on Figure 4 $(a)$ the graph of $\tilde{g}(1-\nu_1(\delta))$ as a function of $\delta$ and $(b)$ the graph of $\tilde{g}(\varepsilon)$ as a function of $\varepsilon$ for various $\delta$. We see that $\tilde{g}$ is maximum for $\lim\limits_{\delta\to 0^+} \tilde{g}(1-\nu_1(\delta)) \underset{\xi_{\min}=1}{\simeq} 0.0046789995$.

Thus, we prove the following Proposition.

**Proposition 3.11** *For any* $\delta \in \left]0\,,\,\frac{1}{2}\right[$ *and for any* $\varepsilon \in ]0\,,\,1-2\delta[$*, there exists* $\kappa_{\delta,\varepsilon} > 0$, $A_{\delta,\varepsilon} > 0$ *and* $C_{\delta,\varepsilon}$ *such that for any memoryless source* $S$ *with binary-part to compress and discrete side-information and for any integer* $\nu_0 > C_{\delta,\varepsilon}$ *– noting* $n = 2^{\nu_0}$ *–, we have*

$$\mathbb{P}\left(Z(S_{\nu_0}^{(J)}) \leq 2^{-n^\delta}\ln 2\right) \;\geq\; 1 - H(S) - \frac{A_{\delta,\varepsilon}}{n^{\varepsilon\kappa_{\delta,\varepsilon}}} \tag{150}$$

$$\mathbb{P}\left(Z'(S_{\nu_0}^{(J)}) \leq 2^{-n^\delta}\right) \;\geq\; H(S) - \frac{A_{\delta,\varepsilon}}{n^{\varepsilon\kappa_{\delta,\varepsilon}}}, \tag{151}$$

*where* $J$ *is a random variable uniformly distributed over* $[\![0\,,\,n-1]\!]$.

*Moreover putting* $B_{\delta,\varepsilon} = \left\lceil \frac{\left(1-\frac{2\delta}{1-\varepsilon}\right)^2 + 2\ln 2}{2\ln(1/\Lambda)} \right\rceil$*, we can choose* $A_{\delta,\varepsilon}$ *such that*

$$\sqrt{e}\left(1 + \frac{2-\varepsilon}{\varepsilon}B_{\delta,\varepsilon}\right) + \frac{\sqrt{\ln 2}}{2} < A_{\delta,\varepsilon} < \sqrt{e}\left(1 + \frac{2-\varepsilon}{\varepsilon}B_{\delta,\varepsilon}\right) + \frac{\sqrt{\ln 2}}{2} + \frac{2}{\ln 2}. \tag{152}$$

Further, for any $\delta \in ]0\,,\,\frac{1}{2}[$, for any memoryless source $S$ with binary-part to compress and discret side-information, for any $\nu_0 \in \mathbb{N}$, let us apply inequality (42) of Proposition 2.5 with

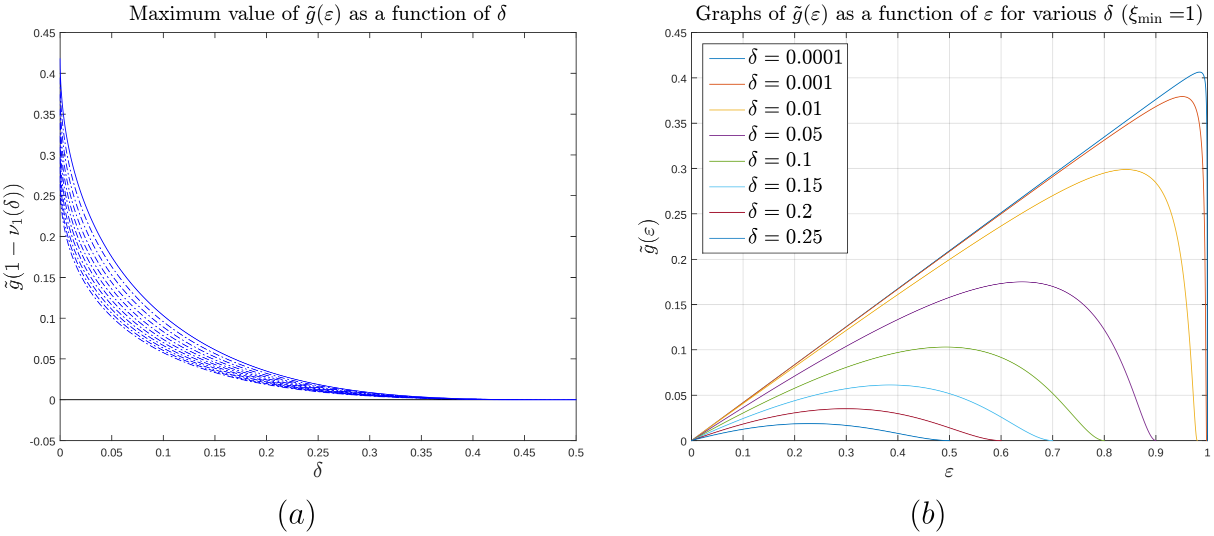


Figure 4: *Graphs of* (*a*) $\tilde{g}(1-\nu_1(\delta))$ *as a function of* $\delta$ *and various values of* $\xi_{\min} = (10+k)/10$ *(*$0 \le k \le 10$*) (solid line corresponds to* $\xi_{\min} = 1$*) and* (*b*) *of* $\tilde{g}(\varepsilon)$ *as a function of* $\varepsilon$ *for various* $\delta$ *and* $\xi_{\min} = 1$.

$\mu = \nu_0$, $m = n = 2^{\nu_0}$ and $\theta = 2^{-n^\delta}$ (provided that $\theta \le \frac{1}{2}$):

$$\frac{|\mathcal{H}_{X|Y}(2^{-n^\delta}) \cap \mathcal{V}^c_{X|Y}(2^{-n^\delta})|}{n} \le \frac{\sqrt{2}\Lambda^{\nu_0}}{2\sqrt{2^{-n^\delta}}}. \tag{153}$$

We claim that for any $\kappa > 0$, for any $\varepsilon > 0$, there exists $\theta_{\kappa,\varepsilon} > 0$ such that for all $n > \theta_{\kappa,\varepsilon}$

$$\frac{\sqrt{2}\Lambda^{\nu_0}}{2\sqrt{2^{-n^\delta}}} \le \frac{\theta_{\kappa,\varepsilon}}{n^{\kappa\varepsilon}}. \tag{154}$$

Indeed, inequality (154) is equivalent to

$$\log n \left(\log \Lambda + \kappa\varepsilon\right) \le \log \theta_{\kappa,\varepsilon} + \frac{1}{2}\left(1 + n^\delta\right), \tag{155}$$

which is satisfied when $\theta_{\kappa,\varepsilon}$ and $n$ are sufficiently large.

Finally we have

$$\mathbb{P}\left(H(S^{(J)}_{\nu_0}) > 2^{-n^\delta}\right) = 1 - \mathbb{P}\left(H(S^{(J)}_{\nu_0}) \le 2^{-n^\delta}\right) \tag{156}$$

and since according to relation (12),

$$Z(S^{(J)}_{\nu_0}) \le 2^{-n^\delta} \ln 2 \quad \Rightarrow \quad H(S^{(J)}_{\nu_0}) \le \log\left(1 + Z(S^{(J)}_{\nu_0})\right) \le \frac{Z(S^{(J)}_{\nu_0})}{\ln 2} \le 2^{-n^\delta}, \tag{157}$$

then

$$\mathbb{P}\left(H(S^{(J)}_{\nu_0}) \le 2^{-n^\delta}\right) \ge \mathbb{P}\left(Z(S^{(J)}_{\nu_0}) \le 2^{-n^\delta} \ln 2\right) \tag{158}$$

and with the notations of Proposition 3.11, we have

$$\mathbb{P}\left(H(S^{(J)}_{\nu_0}) > 2^{-n^\delta}\right) \le H(S) + \frac{A_{\delta,\varepsilon}}{n^{\varepsilon\kappa_{\delta,\varepsilon}}} \tag{159}$$

Similarly

$$Z'(S^{(J)}_{\nu_0}) \le 2^{-n^\delta} \quad \Rightarrow \quad H(S^{(J)}_{\nu_0}) \ge 1 - 2^{-n^\delta} \tag{160}$$

implies

$$\mathbb{P}\left(H(S^{(J)}_{\nu_0}) > 1 - 2^{-n^\delta}\right) \ge \mathbb{P}\left(Z'(S^{(J)}_{\nu_0}) \le 2^{-n^\delta}\right) \ge H(S) - \frac{A_{\delta,\varepsilon}}{n^{\varepsilon\kappa_{\delta,\varepsilon}}}. \tag{161}$$

Therefore, applying the left inequality of relation (43) and the right inequality of relation (44) we prove the following proposition.

**Proposition 3.12** *For any $\delta \in \left]0\,,\,\frac{1}{2}\right[$ and for any $\varepsilon \in ]0\,,\,1-2\delta[$, there exists $\kappa_{\delta,\varepsilon} > 0$, $A_{\delta,\varepsilon} > 0$ and $C_{\delta,\varepsilon}$ such that for any memoryless source $S$ with binary-part to compress and discrete side-information and for any integer $\nu_0 > C_{\delta,\varepsilon}$ – noting $n = 2^{\nu_0}$ –, we have*

$$0 \leq \frac{|\mathcal{H}_{X|Y}(2^{-n^\delta}) \cap \mathcal{V}^c_{X|Y}(2^{-n^\delta})|}{n} \leq \frac{A_{\delta,\varepsilon}}{n^{\varepsilon\kappa_{\delta,\varepsilon}}} \tag{162}$$

$$H(S) - 2^{-n^\delta} \leq \frac{\left|\mathcal{H}_{X|Y}\left(2^{-n^\delta}\right)\right|}{n} \leq H(S) + \frac{A_{\delta,\varepsilon}}{n^{\varepsilon\kappa_{\delta,\varepsilon}}} \tag{163}$$

$$H(S) - \frac{A_{\delta,\varepsilon}}{n^{\varepsilon\kappa_{\delta,\varepsilon}}} \leq \frac{\left|\mathcal{V}_{X|Y}\left(2^{-n^\delta}\right)\right|}{n} \leq H(S) + 2^{-n^\delta}, \tag{164}$$

*where $A_{\delta,\varepsilon}$ satisfies the inequalities (152).*

## 3.4 Order of magnitude of constants

In this subsection we study the values of the constants $c_\beta$ and $c_\delta$ with numerical simulations. Firstly we compute the solution[9] $\alpha = \alpha(\beta)$ of the equation

$$\varphi\left(\frac{\log\alpha - \log\beta}{\beta}\right) = 1 \quad \Leftrightarrow \quad \frac{\beta}{\alpha} + \frac{\beta}{\log\alpha - \log\beta} = \beta, \tag{165}$$

such that

$$c_\beta^{(1)} = \frac{1}{\beta}\log\left(\frac{\alpha(\beta)}{\beta}\right) \tag{166}$$

is the smallest permissible value of $c_\beta$ satisfying (68). Moreover we find a simple expression

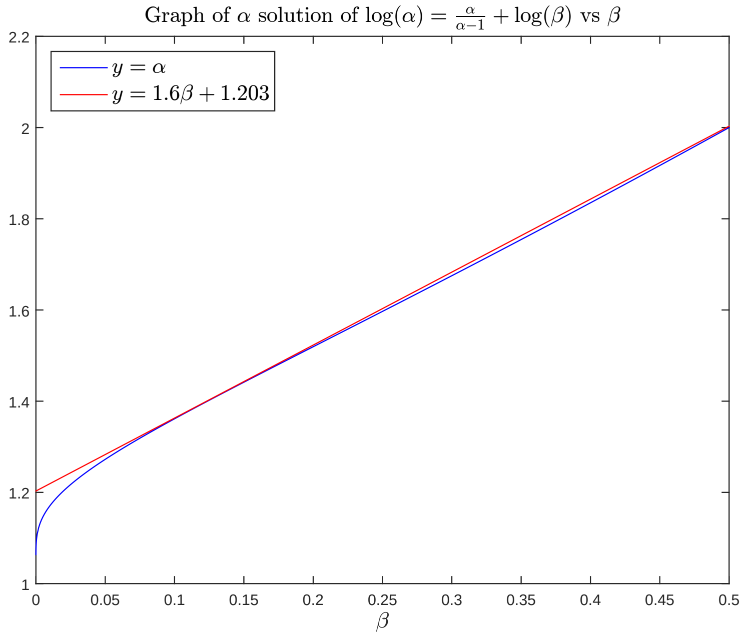


Figure 5: *Graphs of $\alpha(\beta)$ solution of equation (165) and of an affine upper bound.*

$$c_\beta^{(2)} = \frac{1}{\beta}\log\left(\frac{1.6\beta + 1.203}{\beta}\right) \tag{167}$$

slightly greater than the smallest value $c_\beta^{(1)}$ (see Figures 5–7).

[9]Let us remark that this new function $\alpha$ is not connected to the $\alpha$ function introduced in (129).

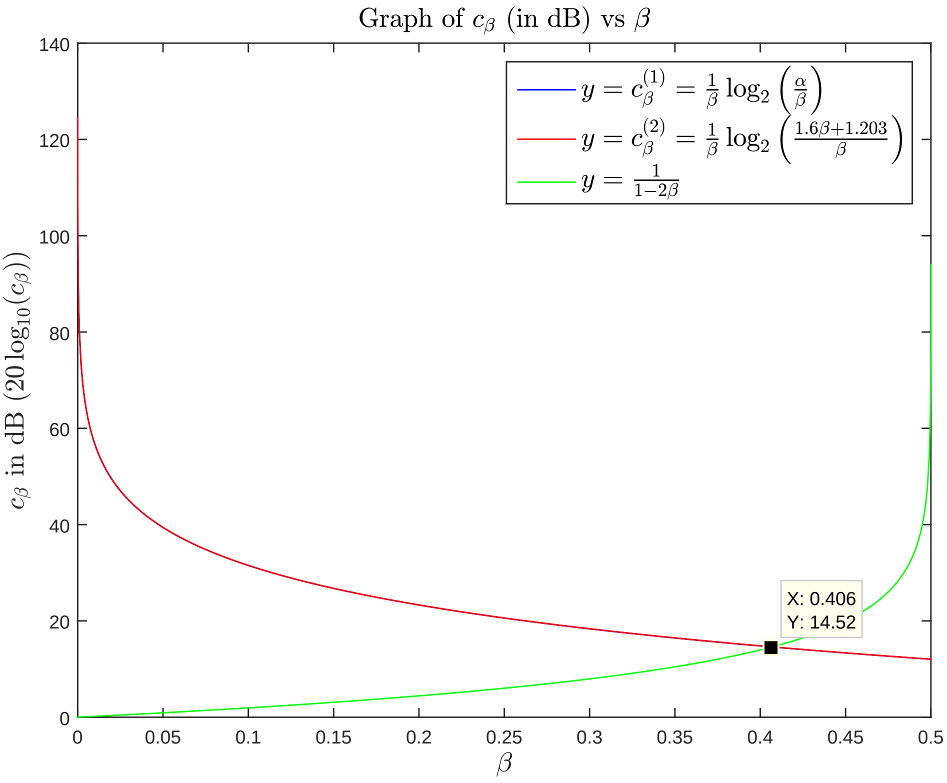


Figure 6: *Graphs of* $c_\beta^{(1)}$, $c_\beta^{(2)}$ *and* $\frac{1}{1-2\beta}$.

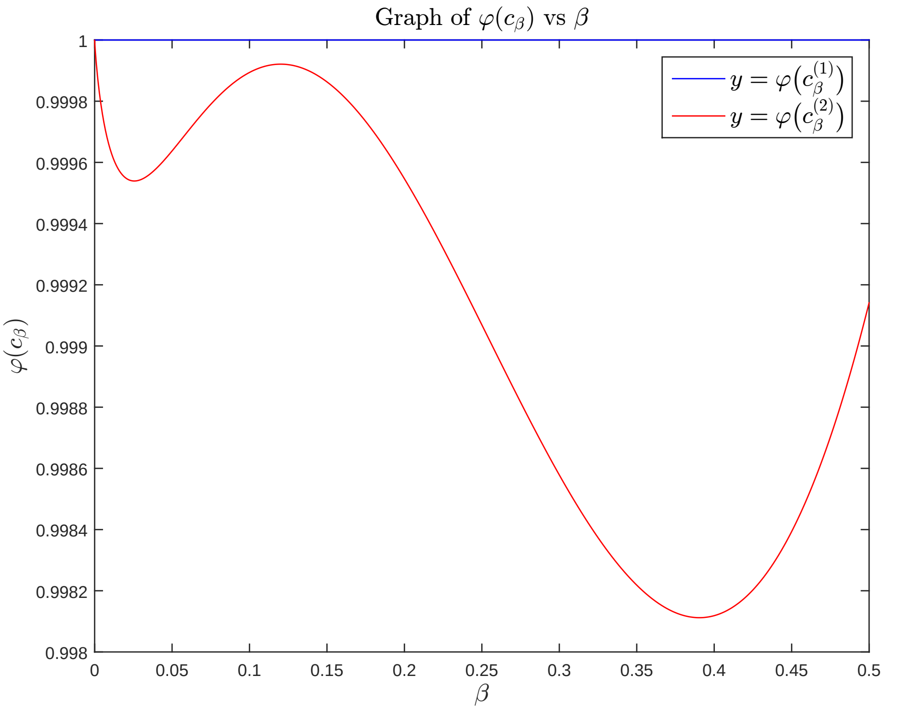


Figure 7: *Graphs of* $\varphi\left(c_\beta^{(1)}\right)$ *and* $\varphi\left(c_\beta^{(2)}\right)$.

Secondly, we assume that inequality (134) is an equality and replacing $\alpha$ by the expression (129), we obtain

$$\log(1/\rho) = \frac{\log(1/\Lambda)}{1+\alpha} = \frac{2\xi \ln(1/\Lambda)}{2\xi \ln 2 + (1-2\beta)^2} \tag{168}$$

and we express $c'_\beta$ and $c_\delta$ introduced in relations (71,72) as functions of $\beta$ and $\xi$.

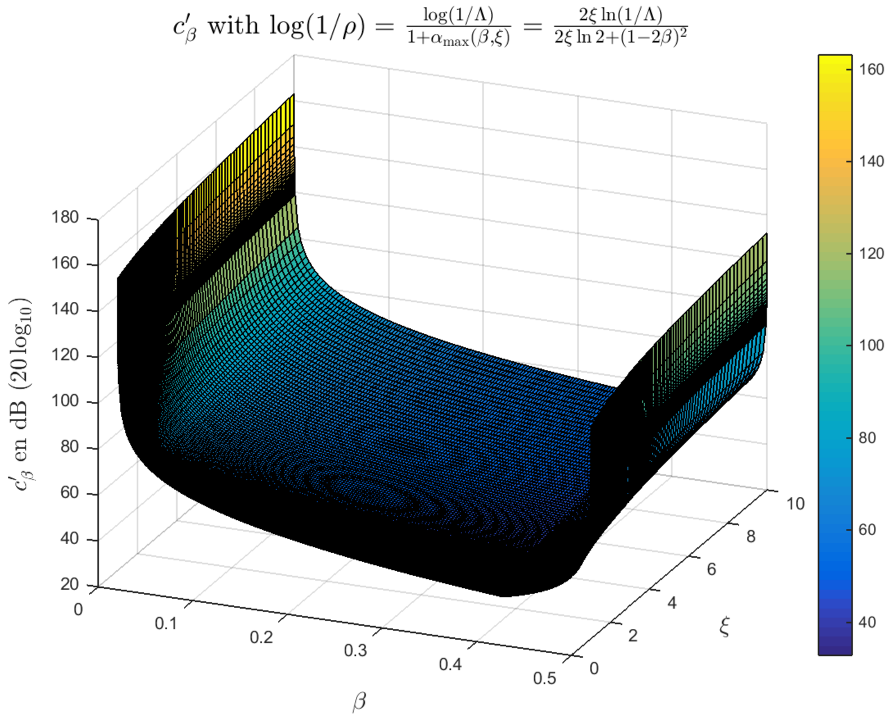


Figure 8: *Graphs of $c'_\beta$ versus $\xi$ and $\beta$.*

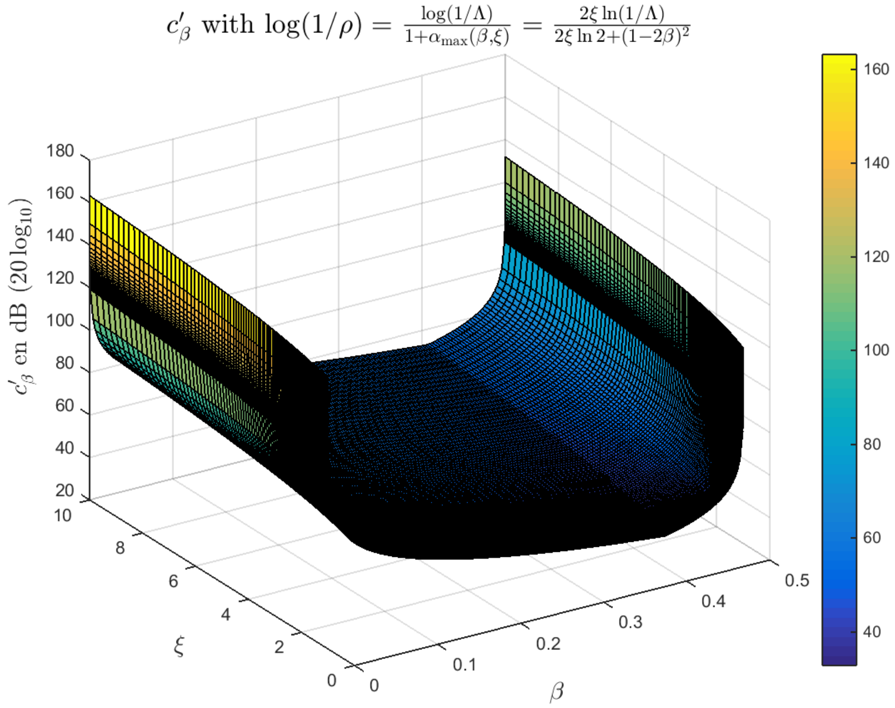


Figure 9: *Graphs of $c'_\beta$ versus $\xi$ and $\beta$.*

Let us denote

$$c_\delta^{(1)} = \frac{(1+\alpha_0)\xi}{(\xi - 1)\log(1/\rho)} \tag{169}$$

appearing in the definition (72) of $c_\delta$.

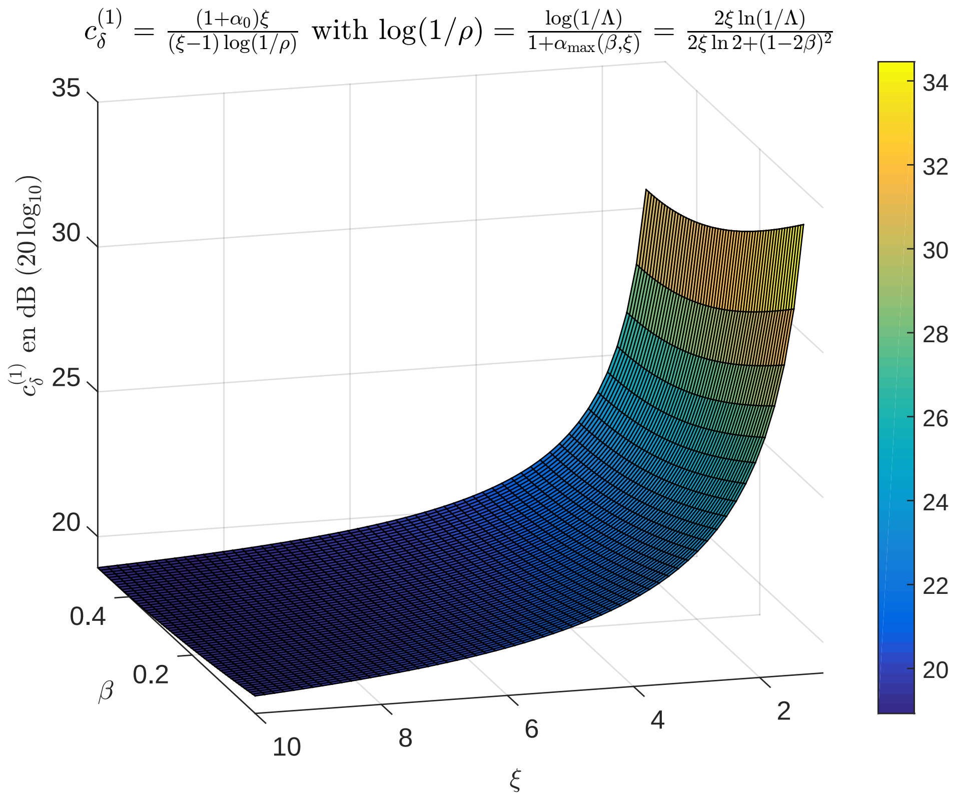


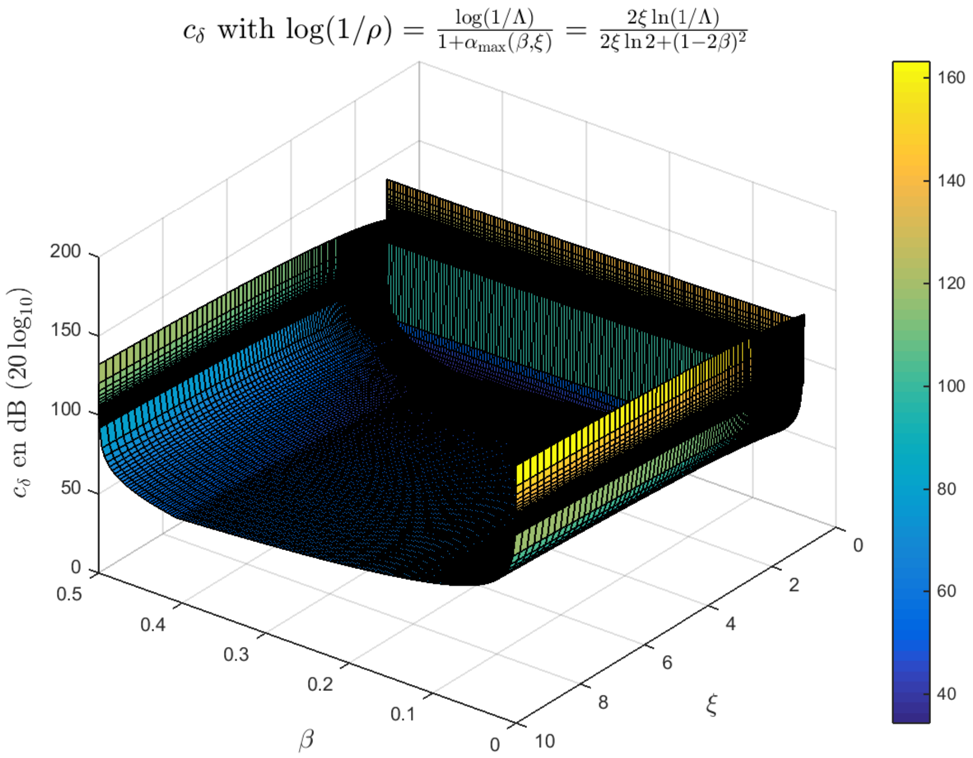

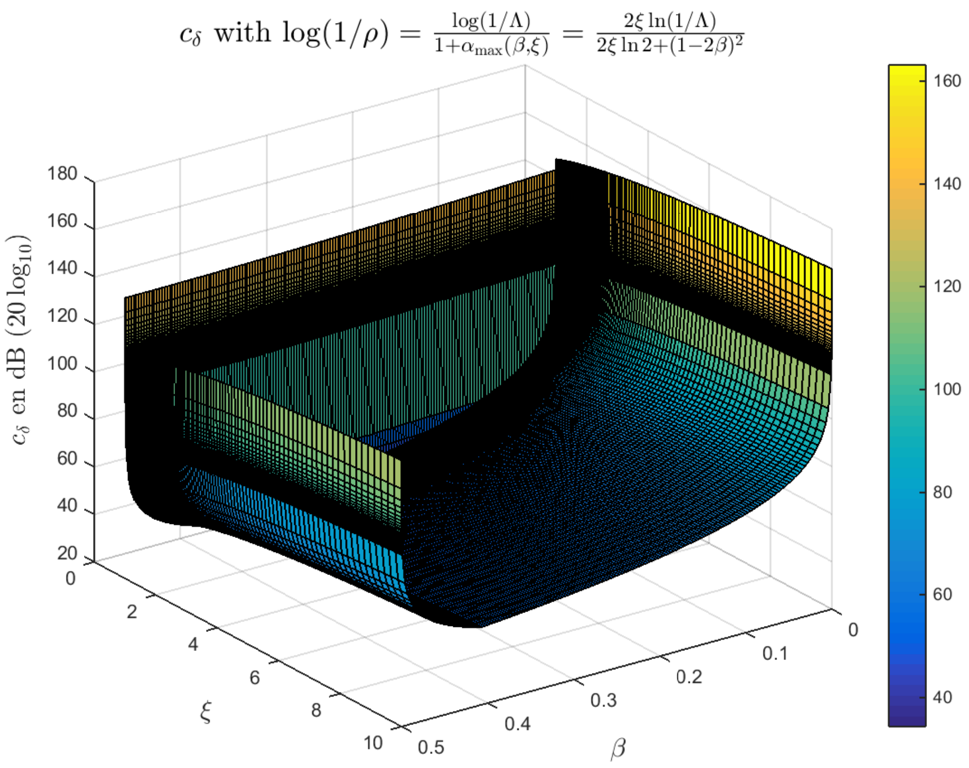


Figure 10: *Graphs of $c_\delta$ versus $\xi$ and $\beta$.*

In order to have

$$c \overset{\text{def}}{=} \left\lceil \frac{\gamma\xi}{\log(1/\rho)} \right\rceil = \frac{\gamma\xi}{\log(1/\rho)} \tag{170}$$

with $\rho$ satisfying equality (168) and $\xi > 1$ as small as possible, we set $\xi_{\min}$ as the value of $\xi$ solution of equations (168) and

$$\frac{(1-\varepsilon)\xi}{\varepsilon\log(1/\rho)} = \min\left(\mathbb{N}^* \cap \left\{\frac{(1-\varepsilon)\xi}{\varepsilon\log(1/\rho)} \ :\ \xi > 1\right\}\right). \tag{171}$$

Table 1: Some numerical values obtained by simulations.

| $\delta$ | $\varepsilon$ | $\beta$ | $\gamma$ | $c_\beta^{(2)}$ | $\frac{1}{1-2\beta}$ | $\xi_{\min}$ | $\log(\frac{1}{\rho})$ | $c'_\beta$ | $c_\delta$ | $A_{\delta,\varepsilon}$ | $C_{\delta,\varepsilon}$ | $\kappa_\epsilon = \kappa_{\delta,\varepsilon}$ | $\varepsilon\kappa_\epsilon$ |
|---|---|---|---|---|---|---|---|---|---|---|---|---|---|
| 0.10 | 0.79 | 0.48 | 0.27 | 4.3 | 21 | 1.42 | 0.1255 | 316 | 316 | 30.1 | 400 | $1.14\,10^{-4}$ | $9.05\,10^{-5}$ |
| 0.10 | 0.74 | 0.38 | 0.35 | 5.8 | 4.3 | 1.03 | 0.121 | 66 | 506 | 25.6 | 684 | $3.33\,10^{-3}$ | $2.46\,10^{-3}$ |
| 0.10 | 0.69 | 0.32 | 0.45 | 7.5 | 2.8 | 1.03 | 0.116 | 83 | 650 | 29.5 | 943 | $7.04\,10^{-3}$ | $4.86\,10^{-3}$ |
| 0.10 | 0.64 | 0.28 | 0.56 | 9.2 | 2.3 | 1.20 | 0.112 | 115 | 115 | 39.0 | 179 | $8.54\,10^{-3}$ | $5.47\,10^{-3}$ |
| 0.10 | 0.59 | 0.24 | 0.69 | 11.1 | 2.0 | 1.08 | 0.107 | 127 | 270 | 41.3 | 458 | $1.11\,10^{-2}$ | $6.53\,10^{-3}$ |
| 0.10 | 0.54 | 0.22 | 0.85 | 13.0 | 1.8 | 1.10 | 0.104 | 153 | 223 | 48.7 | 413 | $1.12\,10^{-2}$ | $6.36\,10^{-3}$ |
| 0.10 | 0.49 | 0.20 | 1.04 | 15.1 | 1.6 | 1.06 | 0.101 | 173 | 352 | 55.3 | 719 | $1.24\,10^{-2}$ | $6.05\,10^{-3}$ |
| 0.10 | 0.44 | 0.18 | 1.27 | 17.1 | 1.6 | 1.08 | 0.099 | 201 | 268 | 65.9 | 610 | $1.19\,10^{-2}$ | $5.24\,10^{-3}$ |
| 0.10 | 0.39 | 0.16 | 1.56 | 19.3 | 1.5 | 1.04 | 0.096 | 221 | 557 | 75.6 | 1428 | $1.17\,10^{-2}$ | $4.56\,10^{-3}$ |
| 0.10 | 0.34 | 0.15 | 1.94 | 21.5 | 1.4 | 1.01 | 0.093 | 243 | 2394 | 88.7 | 7042 | $1.10\,10^{-2}$ | $3.74\,10^{-3}$ |
| 0.10 | 0.29 | 0.15 | 2.45 | 23.7 | 1.4 | 1.01 | 0.092 | 271 | 1633 | 108.9 | 5631 | $9.78\,10^{-3}$ | $2.83\,10^{-3}$ |
| 0.10 | 0.24 | 0.13 | 3.17 | 26.0 | 1.4 | 1.04 | 0.091 | 304 | 629 | 139.1 | 2622 | $8.26\,10^{-3}$ | $1.98\,10^{-3}$ |
| 0.10 | 0.19 | 0.12 | 4.26 | 28.4 | 1.3 | 1.01 | 0.089 | 326 | 3834 | 178.5 | 20179 | $6.90\,10^{-3}$ | $1.31\,10^{-3}$ |
| 0.10 | 0.14 | 0.12 | 6.14 | 30.8 | 1.3 | 1.01 | 0.088 | 355 | 3168 | 251.3 | 22634 | $5.21\,10^{-3}$ | $7.31\,10^{-4}$ |
| 0.10 | 0.09 | 0.11 | 10.11 | 33.2 | 1.3 | 1.00 | 0.087 | 384 | 8302 | 403.1 | 92253 | $3.44\,10^{-3}$ | $3.10\,10^{-4}$ |
| 0.10 | 0.04 | 0.10 | 24 | 35.7 | 1.3 | 1.00 | 0.087 | 414 | 6290 | 937.4 | 157257 | $1.56\,10^{-3}$ | $6.24\,10^{-5}$ |
